# Impaired midfrontal‑motor theta phase synchronization characterizes maladaptive motivational behavior in people with obsessive‑compulsive disorder

**DOI:** 10.1371/journal.pbio.3003979

**Published:** 2026-09-01

**Authors:** Yu Pang, Dongsheng Zhou, Ziwen Peng, Wanting Liu, Ruojie Huang, Carol A. Seger, Qi Chen

**Affiliations:** 1 School of Psychology, South China Normal University, Guangzhou, China; 2 Department of Psychiatry, Zhejiang Key Laboratory of Drug Addiction & Brain Health, Affiliated Kangning Hospital of Ningbo University (Ningbo Kangning Hospital), Ningbo, China; 3 School of Psychology, Key Laboratory of Brain Cognition and Emotional Health of Guangdong Higher Education Institutes, and Shenzhen Key Laboratory of Affective and Social Cognitive Science, Shenzhen University, Shenzhen, China; 4 Department of Psychology, Colorado State University, Fort Collins, Colorado, United States of America; Institute of Psychology Chinese Academy of Sciences, CHINA

## Abstract

Obsessive‑compulsive disorder (OCD) is characterized by an insight‑action dissociation, in which people with OCD recognize that their behavior is irrational but still struggle to inhibit habitual responses. This dissociation may be related to abnormally strong motivational biases, reflected in excessive tendencies to approach reward and avoid punishment. We employed a motivational Go/NoGo learning task, combined with computational modeling and electroencephalography (EEG), to investigate how 36 people with OCD and 37 healthy controls (HC) regulate maladaptive biases during motivated action. People with OCD showed stronger Pavlovian bias and lower learning rates. Similar to HC, people with OCD also showed increased midfrontal theta power related to conflict detection and to the generation of a control demand to increase the weighting of instrumental action values during choice, suggesting that they were able to detect the mismatch between their behavior and task goals. However, in OCD, conflict‑related theta enhancement overlapped with the response window, indicating that control signals emerged or arrived too late to effectively influence choice. Midfrontal‑motor theta phase synchrony provided the strongest model evidence for the modulation of maladaptive biases in OCD, yet this pathway showed no significant conflict‑related enhancement and failed to effectively modulate motivational biases under conflict. Taken together, these findings suggest a neural mechanism underlying the insight‑action dissociation in OCD and identify midfrontal‑motor theta phase synchrony as a potential treatment target.

## Abstract 2

People with obsessive compulsive disorder recognize that their behavior is irrational but still struggle to inhibit habitual responses. This study shows that disrupted midfrontal-motor theta phase synchrony weakens effective control that could lead to abnormally strong motivational biases and excessive tendencies to approach reward and avoid punishment.

## Introduction

Obsessive‑compulsive disorder (OCD) is characterized by intrusive thoughts (obsessions) and repetitive behaviors (compulsions) [1,2], with ego‑dystonicity serving as its hallmark and distinguishing pathological feature [3]. People with OCD often recognize that their compulsive behaviors are irrational and unnecessary, yet they remain driven by intense anxiety to engage in compulsions [4–6], a characteristic known as the “insight‑action” dissociation [7,8]. Compulsive behaviors provide temporary relief from anxiety, but this relief further reinforces both the behaviors [9,10] and their core motivations (e.g., harm avoidance and feelings of incompleteness) [11–16]. The core deficit in OCD may reflect an imbalance between goal‑directed and habit systems [17–22], together with aberrant motivational processing [23]. The goal‑directed system struggles to effectively inhibit automatic habitual responses driven by strong motivation, leading to the persistence of compulsive behavior even when it is recognized as irrational.

Approaching rewards and avoiding losses constitute core features of the human motivational system [24–26], which shapes actions through Pavlovian and instrumental mechanisms. Pavlovian mechanisms consist of the elicitation of global “approach” or “avoidance” response tendencies by rewarding or aversive cues, respectively [27–29]. In contrast, instrumental learning supports flexible goal‑directed control by acquiring action‑outcome associations [30], and exhibits valence biases during learning [31,32]. More specifically, reward outcomes reinforce Go actions more effectively than NoGo responses, whereas punishment outcomes facilitate the avoidance of Go actions but show a reduced effect on NoGo behaviors [33,34]. Thus, the Pavlovian system encodes stimulus‑outcome associations, whereas the instrumental system governs action‑outcome learning [35]. With repeated training and reinforcement, initially flexible action‑outcome control can gradually become automatized and shift toward cue‑triggered stimulus–response habits [36,37]. This transition is often adaptive because it reduces computational load by providing hardwired action defaults. However, Pavlovian response tendencies become maladaptive when they conflict with instrumental requirements, requiring us to rely more on the relatively flexible yet slower instrumental system and increasing the need for cognitive control.

Recent OCD research suggests that heightened sensitivity to symptom‑relevant cues and elevated harm‑avoidance motivation promote avoidance or safety‑seeking responses [38,39], such as washing or checking. At the same time, these actions are negatively reinforced because they temporarily reduce anxiety or feelings of incompleteness [40,41]. Through repeated reinforcement, these safety behaviors gradually become automatically triggered by cues, forming habitual behaviors that are resistant to extinction [42,43]. Therefore, we hypothesized that people with OCD would exhibit enhanced Pavlovian response tendencies driven by salient cues, paired with an instrumental learning bias characterized by abnormal updating of action‑outcome contingencies. When increased Pavlovian biases conflict with task goals, they can undermine goal‑directed control, allowing automatic responses to dominate and become resistant to extinction.

Extensive research indicates that theta‑band (4–8 Hz) oscillatory activity over the midfrontal cortex (MFC) increases during conflict trials in a wide variety of tasks, reflecting conflict detection and signaling the need for cognitive control [44–49]. Successful conflict resolution is typically accompanied by increased functional connectivity between the MFC and other task‑relevant regions such as the lateral prefrontal cortex (lPFC) and motor cortex [50,51]. This enhanced synchronization is commonly interpreted as a control‑demand signal from the MFC that facilitates goal‑directed responding in the lPFC and inhibits impulsive responses in the motor cortex [52–54].

The Motivational Go/NoGo learning task [50] provides an effective experimental paradigm for testing the interaction between Pavlovian response tendencies and instrumental learning. The task operationalizes motivational conflict by manipulating the congruency between Pavlovian responses and instrumental behaviors. Computational models applied to behavioral data in this task can distinguish Pavlovian response bias from instrumental learning bias.

The current study combined computational modeling with time–frequency and intersite phase synchrony (ISPS) analyses to investigate how the midfrontal network modulates control of maladaptive biases in OCD. Our research addressed two primary questions. First, we evaluated whether local midfrontal theta activity in OCD reliably tracks conflict between the Pavlovian and instrumental systems and generates a control‑demand signal. Second, we examined whether theta phase synchrony between the MFC and lPFC and motor regions supports effective motivational behavior modulation. We hypothesized that conflict‑related midfrontal theta would be locally preserved in OCD, but its regulatory impact would be compromised by reduced synchrony with task‑relevant regions, reflecting inefficient implementation of control. Overall, we aimed to provide a computational and neurodynamic explanation of the insight‑action dissociation in OCD by linking local oscillatory signals to long‑range network coordination.

## Results

### Task performance: Motivated action was biased by cue and outcome valence in OCD

**Fig 1:**
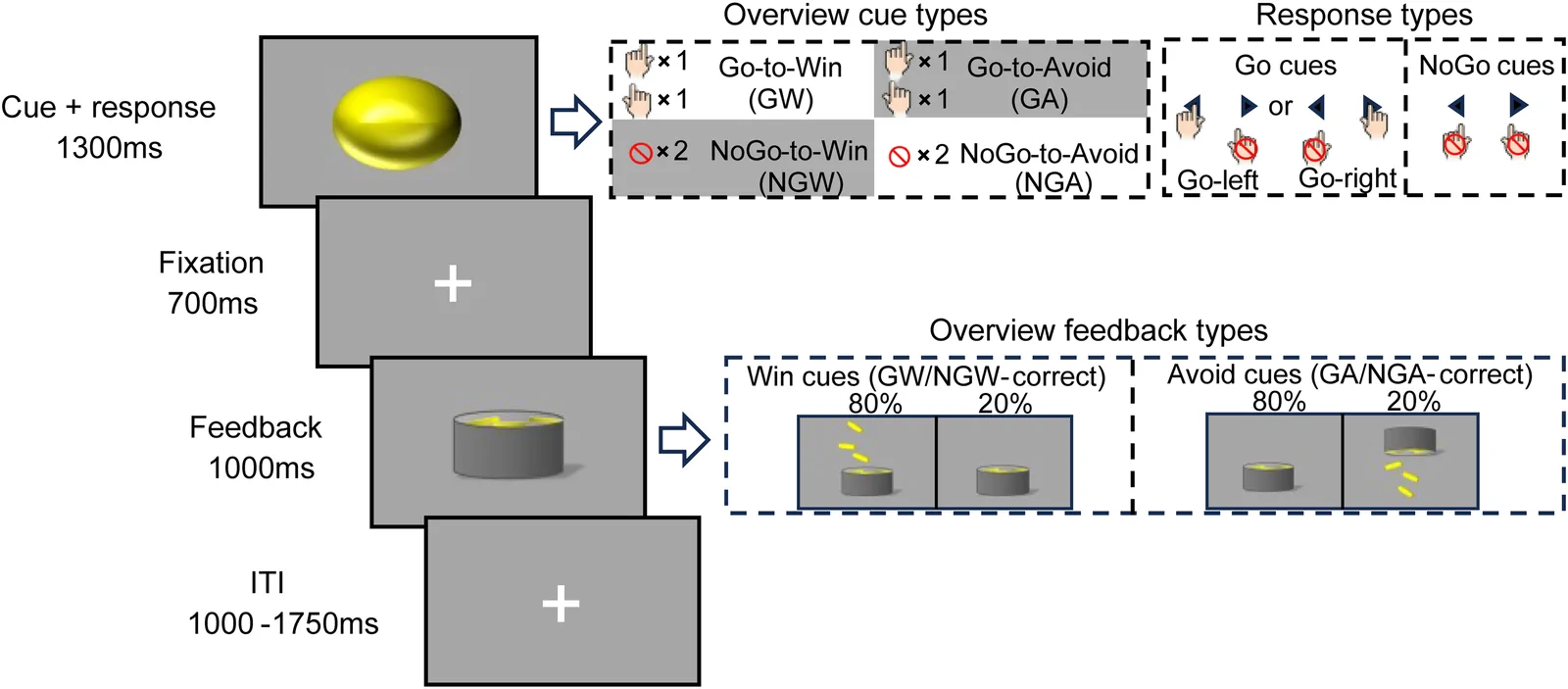
Motivational Go and NoGo learning task. The left panel displays the trial procedure for each trial. As shown in the top middle panel, on each trial one of eight different gem‑shaped cues was presented, equally divided into four types (Go‑to‑Win, GW; Go‑to‑Avoid, GA; NoGo‑to‑Win, NGW; NoGo‑to‑Avoid, NGA) based on cue valence (win vs. avoid) and required action (Go vs. NoGo). GW and NGA trials (white boxes) are motivationally congruent, as the Pavlovian response tendencies are in line with the instrumental requirements, whereas the NGW and GA trials (gray boxes) are motivationally incongruent. Of the eight cues, two were assigned to the NGW, two to NGA, one to GW‑left hand response, one GW‑right hand response, one to GA‑left hand response, and one GA‑right hand response. The top right panel outlines the available response types. Upon cue presentation, participants must choose to perform a Go‑left or Go‑right button press or withhold responding for a NoGo action. The bottom right panel details the probabilistic feedback structure for each cue type. For win cues (GW/NGW), correct responses result in a reward 80% of the time and a neutral outcome 20% of the time. For avoid cues (GA/NGA), correct responses lead to a neutral outcome 80% of the time and a loss 20% of the time. These reinforcement probabilities are reversed for incorrect responses.

We employed a motivational Go/NoGo learning task with multiple active response options (Fig 1). Participants learned via trial and error to respond to visual cues by executing a Go (left or right button press) or NoGo response to gain rewards (“win” cues) and avoid losses (“avoid” cues). Because the task included multiple Go response options, it could distinguish Pavlovian response tendencies driven by cue valence from instrumental learning bias shaped by outcome feedback.

**Fig 2:**
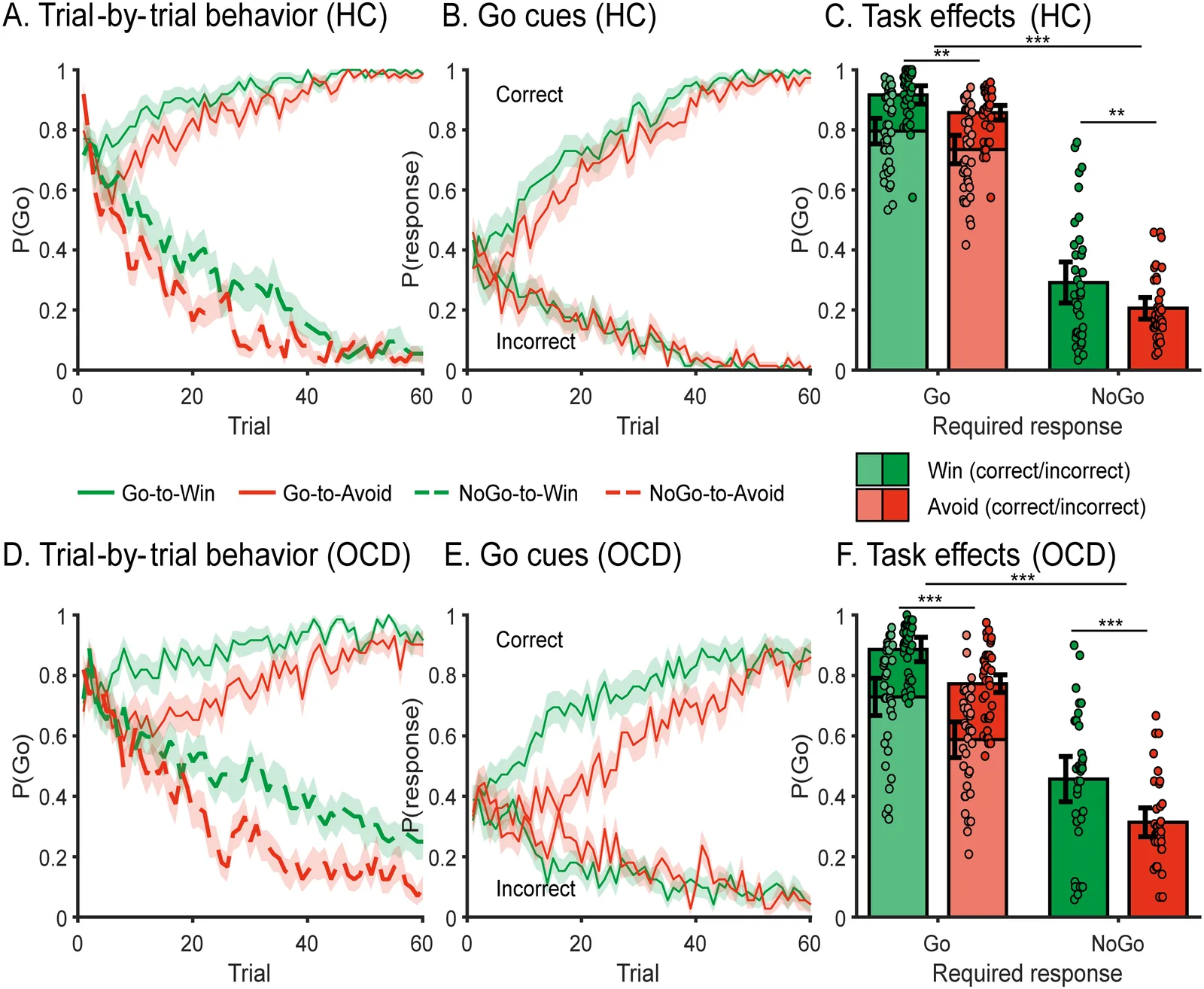
Behavioral performance and task effects in the Motivational Go/NoGo task. **(A, D)** Trial‑by‑trial behavioral performance for healthy controls **(A)** and people with OCD **(D)**. The plots illustrate the trajectories of Go response probabilities across trials for the four cue categories. Green curves represent “win” cues and red curves represent “avoid” cues. Solid lines indicate Go cues, while dashed lines indicate NoGo cues. Shaded areas represent the standard error of the mean (SEM). **(B, E)** Learning curves for Go cues in both groups. These panels display the change in correct (solid) and incorrect (dashed lines) Go responses over time specifically for Go cues. This reflects the learning process of instrumental task requirements. **(C, F)** Overall task effects. Bar plots display the mean Go response probability for each group as a function of required action (Go vs. NoGo) and cue valence (“win” vs. “avoid”). Asterisks indicate significance levels (^*^p < 0.05, ^**^p < 0.01, ^***^p < 0.001). Error bars represent the standard error of the mean. The numerical data underlying panels A–F can be found in S1 Data.

To examine whether participants learned the instrumental actions and to determine whether motivational valence biased behavioral activation, we first analyzed Go response probability. In these logistic mixed‑effects models, required action (Go versus NoGo) and valence (“win” versus “avoid” cues) were entered as within‑subject predictors, and these models were fitted separately within the healthy controls (HC) and OCD groups. Behavioral analyses showed that both groups successfully learned the task rules (Fig 2): go responses occurred more often for Go cues than NoGo cues in both groups (HC: *β* = 1.86, OR = 6.42, *χ*²(1) = 334.05, *p* < 0.001; OCD: *β* = 1.20, OR = 3.32, *χ*²(1) = 94.44, *p* < 0.001). However, relative to “avoid” cues, participants overall made more Go responses for “win” cues (HC: *β* = 0.34, OR = 1.40, *χ*²(1) = 19.76, *p* < 0.001; OCD: *β* = 0.39, OR = 1.48, *χ*²(1) = 41.44, *p* < 0.001), which indicated that motivational valence strongly biased Go responding. To test whether these effects differed between groups, we then fitted a combined logistic mixed‑effects model in which Group (HC versus OCD) was added as a between‑subject predictor, together with required action, valence, and their interactions. This model revealed a significant group × required action interaction (*β* = −0.65, OR = 0.52, *χ*²(1) = 17.07, *p* < 0.001). Compared with HC participants, the OCD group made more Go responses to NoGo cues (*β* = 0.68, OR = 1.98, *z* = 3.62, *p* < 0.001) but fewer Go responses when Go responses were required (*β* = −0.61, OR = 0.54, *z* = −2.76, *p* = 0.006). Furthermore, to determine whether reduced Go responding in OCD was accompanied by impaired performance when Go responses were required, we conducted an additional logistic mixed‑effects model restricted to Go cues. In this model, the dependent variable was Go‑cue accuracy, and valence (“win” versus “avoid” cues), group (HC versus OCD), and their interaction were entered as predictors. Consistent with the pattern above, accuracy on Go cues was lower in the OCD group than in the HC group (*β* = −0.75, OR = 0.47, *χ*²(1) = 8.89, *p* = 0.003). Finally, to assess learning dynamics under motivational conflict, we extracted condition‑specific learning slopes from the trial‑by‑trial Go response probability model described above. These slopes captured the change in Go response probability over trials. For example, for NGW cues, in which the correct response was to withhold a Go response, a more negative slope would indicate faster acquisition of response inhibition (i.e., a rapid reduction in erroneous Go responses). The OCD group showed slower acquisition of response inhibition only for NGW cues (HC_slope_ = −0.011; OCD_slope_ = −0.008; *t*(71) = −2.41, *p* = 0.019, *Cohen’s d* = −0.56), but not for GA cues.

### Compared with healthy controls, people with OCD showed heightened Pavlovian bias and reduced learning rates

Previous studies have shown that motivational biases in Go/NoGo responding come from both cue‑valence‑driven Pavlovian response bias and outcome‑feedback‑driven instrumental learning bias [50]. As shown in Table 1, we built a Behavioral Model Family (M1–M3c) to separate the roles of these two mechanisms in motivated action selection. We compared model evidence using the Watanabe‑Akaike Information Criterion (WAIC), which provides a complexity‑penalized estimate of model fit and reduces the likelihood that improved fit merely reflects over‑parameterization. M1 was a baseline reinforcement‑learning model with only learning rate (*ε*) and feedback sensitivity (*ρ*). M2 added a general Go bias (b). M3a further added a Pavlovian bias (*π*) to capture response tendencies driven by cue valence. M3b added an instrumental learning bias (*κ*) to capture asymmetric learning shaped by outcome valence and required action. M3c combined all of these parameters to test whether motivational biases could be explained by the combined effects of Pavlovian response bias and instrumental learning bias.

**Table 1:** Stepwise comparison of hierarchical computational modeling frameworks.

Label	WAIC		*R*^2^(%)		Parameter
	HC	OCD	HC	OCD	
M1	19939.26	26783.45	56.62	36.33	*ε*₀, *ρ*
M2	19638.21	25920.65	57.44	38.98	*ε*₀, *ρ*, *b*
M3a	19148.87	25323.10	58.86	40.76	*ε*₀, *ρ*, *b*, *π*
M3b	18854.64	24783.93	58.99	41.60	*ε*₀, *ρ*, *b*, *κ*
M3c	18585.78	24543.96	59.75	42.41	*ε*₀, *ρ*, *b*, *π*, *κ*
M4a	18491.71	24433.90	60.03	42.78	β*θ_*t*_, *π*
M4b	18454.40	24419.47	60.04	42.83	β*θ_*t*_, *Q*
M4c	18493.01	24443.42	59.83	42.59	β*θ_*t*_, τ
M4d	18557.02	24523.14	59.81	42.45	β*θ_*t*_, *κ*
M5a	18435.32	24423.81	60.07	42.85	β*ISPS_lPFC,t_, *Q*
M5b	18483.58	24372.24	60.08	43.05	β*ISPS_motor_contra,t_, *Q*
M5c	18484.47	24435.22	60.06	42.77	β*ISPS_lPFC,t_, *π*
M5d	18476.93	24339.93	60.07	43.13	β*ISPS_motor_contra,t_, *π*

Note: Model performance was evaluated using a hierarchical computational modeling framework (M1–M5d), with WAIC and *R^2^* as indices of model fit; lower WAIC values indicate better fit. Model development proceeded in three stages. First, the Behavioral Model Family (M1–M3c) was used to dissociate the mechanisms underlying motivational biases in action selection, including the learning rate (*ε*), feedback sensitivity (*ρ*), general Go bias (*b*), Pavlovian bias (*π*), and instrumental learning bias (*κ*). Second, the Midfrontal Theta Modulation Family (M4a–M4d) tested whether trial‑by‑trial midfrontal theta power (*θ*_*t*_) modulated different motivational biases of action selection. Third, the ISPS Modulation Family (M5a–M5d) examined whether trial‑by‑trial theta phase synchrony between the MFC and lPFC (ISPS_lPFC,t_) or between the MFC and contralateral motor cortex (ISPS_motor_contra,t_) was associated with the modulation of motivational biases.

**Fig 3:**
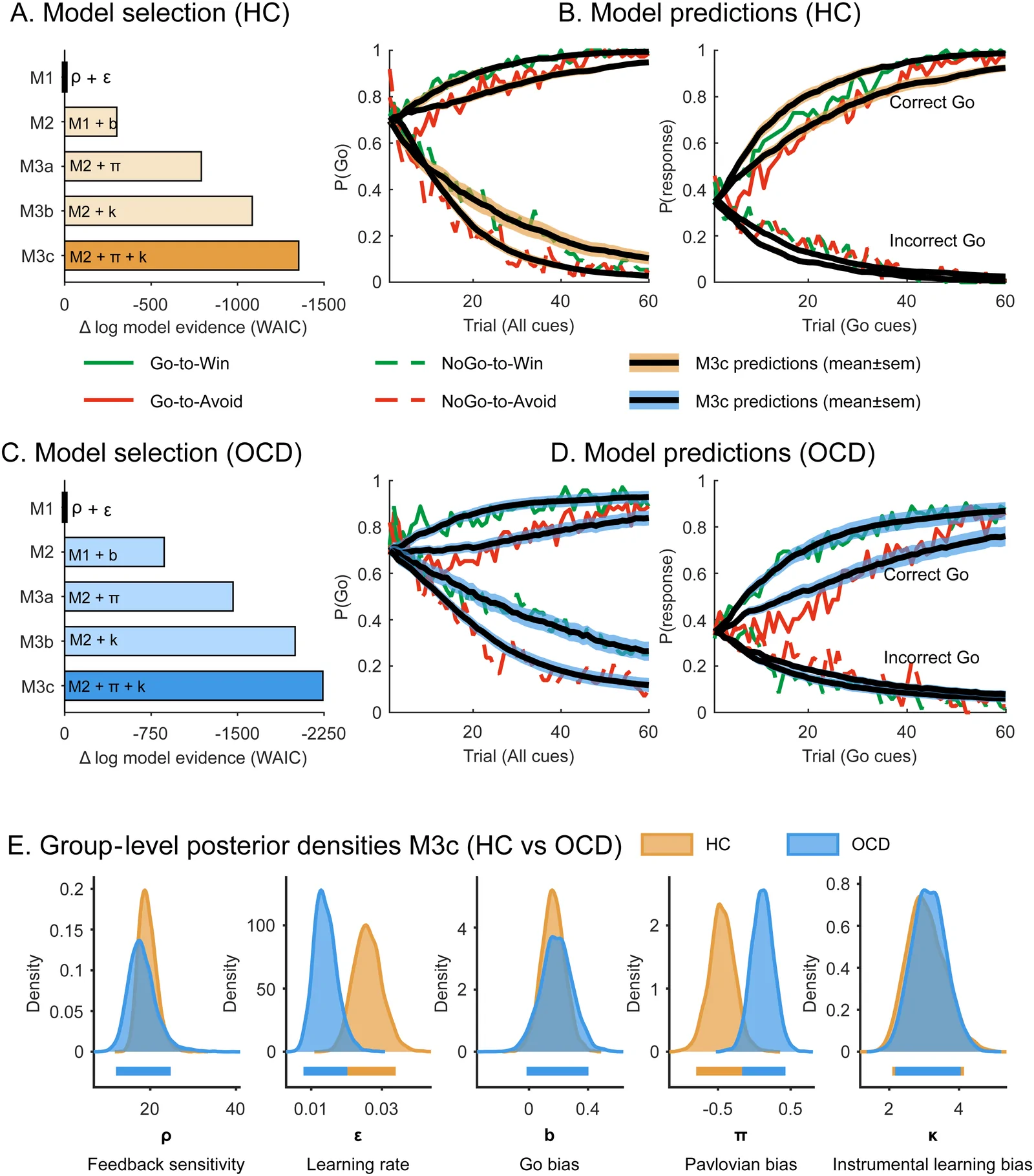
Computational modeling of Pavlovian response bias and instrumental learning bias. **(A, C)** Model selection in HC **(A)** and OCD **(C)**. Bars show model evidence relative to the simplest model, Model 1 (M1) (Δlog model evidence; WAIC‑based). M1 included only the learning rate (*ε*) and feedback sensitivity (*ρ*). M2 added a nonspecific Go bias **(b)**. M3a further added a Pavlovian bias (*π*) to capture cue‑valence–driven response tendencies, whereas M3b added an instrumental learning‑bias term (*κ*) allowing asymmetric learning as a function of outcome valence (win vs. avoid) and required action (Go vs. NoGo). M3c combined *ε*, *ρ*, *b*, *π*, and *κ* and provided the best fit in both groups (dark bars). **(B, D)** Absolute post hoc model fit in HC **(B)** and OCD **(D)**. The predictions of the winning model M3c (HC: orange; OCD: blue; shaded areas indicate the standard error of the mean) captured the key behavioral features (colored lines). **(E)** Group‑level posterior distributions (HC vs. OCD). Posterior density plots show the group‑level distributions of the five core parameters of model M3c for HC (orange) and OCD (blue). Horizontal bars indicate the 95% highest‑density interval (HDI), illustrating the degree of overlap and divergence between groups. The numerical data underlying panels A–E can be found in S2 Data.

As shown in Fig 3A and 3C, and in Table 1, in both groups, Model M3c, which incorporated both Pavlovian bias (*π*) and instrumental learning bias (*κ*), provided the best fit (HC: WAIC = 18585.78, ΔWAIC = −1353.48, *R*^2^ = 59.75%; OCD: WAIC = 24543.96, ΔWAIC = −2239.49, *R*^2^ = 42.41%) when compared with models without either or both of those parameters (M1, M2, M3a, and M3b). As shown in Fig 3B and 3D, actual average participant behavior was close to the predictions of the winning model. Altogether, our results indicated that instrumental responding was biased by both cue valence and outcome valence, consistent with previous research [50]. Moreover, as shown in Fig 3E, group‑level posterior comparisons from M3c (Δ = HC − OCD) indicated a stronger Pavlovian bias in OCD (Δ*π* ≈ −0.56, 95% highest‑density interval (HDI) [−1.04, −0.04], *P*(Δ*π* < 0) = 0.991), along with a higher learning rate in HC than OCD (Δ*ϵ* ≈ +0.012, 95% HDI [0.003, 0.022], *P*(Δ*ϵ* > 0) = 0.986).

### Midfrontal theta in OCD supports conflict detection and modulation of motivational biases

After confirming that motivational biases in people with OCD were affected by both Pavlovian bias and instrumental learning bias, we next examined the neural mechanisms that modulate these biases during motivational conflict. In previous studies, conflict detection has most often been measured using event‑related potentials, especially the N2 component [55,56]. However, the results of studies examining the N2 in OCD have been inconsistent [57–60], making it difficult to reach a clear conclusion about conflict detection in this group. In contrast, midfrontal theta is considered a more reliable time‑frequency marker of conflict detection [49,61,62] and may also play a role in the dynamic regulation of downstream cognitive control processes [54,63]. Based on this, we examined midfrontal theta activity in people with OCD during motivational conflict and tested whether midfrontal theta was involved in modulating motivational biases. We hypothesized that, compared with motivationally congruent cues (Go‑to‑Win, NoGo‑to‑Avoid), motivationally incongruent cues (Go‑to‑Avoid, NoGo‑to‑Win) would elicit stronger midfrontal theta power. We further hypothesized that this increase would be especially strong in trials where motivational conflict was correctly resolved. To test these hypotheses, we performed a time‑based permutation test on correct trials over predefined midfrontal channels.

**Fig 4:**
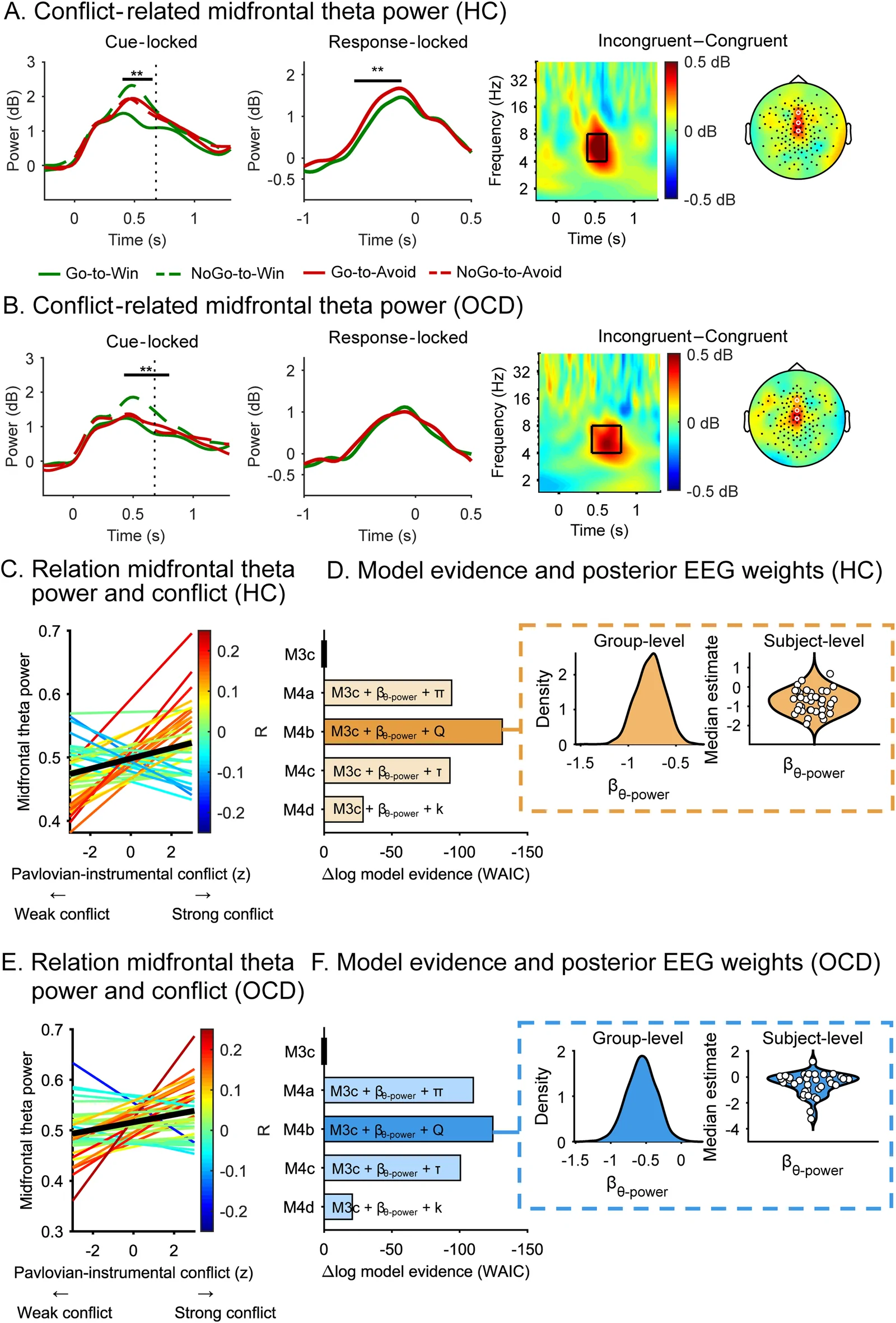
Cue‑locked midfrontal theta power and theta‑modulated computational modeling. **(A, B)** Conflict‑related midfrontal theta power in HC **(A)** and OCD **(B)**. Left: Cue‑locked midfrontal theta power (4–8 Hz) over time for motivationally congruent trials (Go‑to‑Win, NoGo‑to‑Avoid) and incongruent trials (NoGo‑to‑Win, Go‑to‑Avoid). Horizontal black bars indicate time windows showing significant effects based on permutation testing; the dashed vertical line marks the mean reaction time (RT). Middle: Response‑locked midfrontal theta power time courses (significance annotations as in the cue‑locked plots). Right: Time‑frequency maps of the incongruent minus congruent contrast within the midfrontal region. Boxes denote the theta time‑frequency window used for subsequent analyses; the scalp topography to the right shows theta power averaged within this window. **(C, E)** Trial‑wise relationship between cue‑locked theta power and conflict strength in HC **(C)** and OCD **(E)**. Conflict strength (z) was derived from the winning behavioral model M3c and used to predict trial‑by‑trial cue‑locked midfrontal theta power. Colored lines show within‑subject regression slopes (color‑coded by association strength), and the black line indicates the group‑level trend across participants. **(D, F)** Model comparison for theta‑modulated extensions and posterior distributions of *β*_*θ*‑power_ in HC **(D)** and OCD **(F)**. Panels report model evidence for theta‑modulated extensions (see Table 1) relative to the baseline behavioral model M3c (ΔWAIC). M3c contains no EEG modulator. In the extended models, M4a tests whether theta power scales Pavlovian bias (*π*), M4b tests whether theta power scales the contribution of learned instrumental action values (*Q*) to choice, M4c tests whether theta power scales the balance between Pavlovian and instrumental contribution (τ), and M4d tests whether theta power scales instrumental learning bias (*κ*). Insets show posterior distributions of *β*_*θ*‑power_ at the group‑level (posterior density) and at the subject‑level (participant‑specific median estimates). The numerical data underlying panels C–F can be found in S3 Data.

The first step in the electroencephalography (EEG) analysis was to investigate whether midfrontal theta reflects the detection of conflict as found in previous research [50]. If midfrontal theta is involved in conflict detection, it should not only distinguish motivationally incongruent from congruent cues, but also increase as Pavlovian‑instrumental conflict becomes stronger under incongruent conditions. Therefore, we first assessed whether cue‑locked midfrontal theta differentiated motivationally incongruent from congruent cues across groups. More specifically, we performed a time‑based permutation test on cue‑locked, trial‑averaged midfrontal theta power in the 4–8 Hz band to identify time windows showing significant differences between motivationally incongruent and congruent cues. This analysis was restricted to correct trials to minimize the potential contribution of error‑related theta activity. As shown in Fig 4A and 4B, cluster‑based permutation tests confirmed that both groups showed robust conflict‑related theta power increases within cue‑locked windows (HC: 400–650 ms, cluster‑corrected *p* = 0.002; OCD: 424–800 ms, cluster‑corrected *p* = 0.002). Cluster‑window averaged contrasts confirmed large effects in both the HC group (HC: *t*(36) = 6.67, *p* < 0.001, *Cohen’s d*_*z*_ = 1.10) and the OCD group (OCD: *t*(35) = 4.73, *p* < 0.001, *Cohen’s d*_*z*_ = 0.79). We nex*t* tested the relationship between midfrontal theta power and Pavlovian‑instrumental conflict strength. In this study, Pavlovian‑instrumental conflict was defined as the extent to which the instrumental value of NoGo exceeded that of Go in win trials, and the extent to which the instrumental value of Go exceeded that of NoGo in avoid trials (Equation 7). As shown in Fig 4C and 4E, trial‑by‑trial analyses further revealed that midfrontal theta power positively correlated with Pavlovian‑instrumental conflict strength in both groups (HC: *t*(36) = 2.54, *p* = 0.016, *Cohen’s d*_*z*_ = 0.42; OCD: *t*(35) = 4.80, *p* < 0.001, *Cohen’s d*_*z*_ = 0.80). Consis*t*en*t* with these findings, an exploratory event‑related potential (ERP) analysis showed significantly enhanced midfrontal N2 responses to incongruent relative to congruent cues in both groups, with no reliable group difference during incongruent trials (see Text B and Figs A**–**B in S1 File) [57, 58, 64–69]. Together, these findings suggested that midfrontal theta in the OCD group not only distinguished motivational conflict conditions, but also increased with conflict strength, with convergent N2 evidence further supporting preserved cue-level conflict detection.

To examine whether the magnitude of conflict‑related theta enhancement differed between groups, we extracted the theta contrast (incongruent–congruent) from the significant cue‑locked time window and compared this contrast between HC and OCD participants. Between‑group comparisons showed no significant difference in conflict‑related theta amplitude (incongruent–congruent contrast: *t*(71) = 1.19, *p* = 0.237, *Cohen’s d* = 0.28), but temporal profiles differed between groups. As shown in Fig 4A, in the HC group, cue‑locked theta enhancement preceded mean reaction time (679 ± 204 ms), and a subsequent response‑locked analysis identified a robust pre‑response component (−550 to −126 ms; cluster‑corrected *p* = 0.002). The cluster‑window averaged contrast further confirmed stronger theta power for Go‑to‑Avoid than Go‑to‑Win trials (*t*(36) = 4.28, *p* < 0.001, *Cohen’s d*_*z*_ = 0.70). However, in the OCD group the cue‑locked theta window partially overlapped mean reaction time (700 ± 208 ms), and no reliable pre‑response theta component was observed (Fig 4B). Notably, trial‑level linear mixed‑effects analyses showed that the between‑group difference in conflict‑related pre-response theta remained reliable after controlling for trial‑level RT, subject‑level RT variability, and trial order (see Text C and Fig C in S1 File). Furthermore, mean reaction times did not differ significantly between HC and OCD groups (*t*(71) = −0.44, *p* = 0.661, Cohen’s *d* = −0.10). Thus, al*t*hough both groups showed robust cue-locked midfrontal theta related to motivational conflict, only the HC group showed a reliable conflict-related theta enhancement before successful response execution.

We further tested whether pre‑response theta was directly related to behavioral performance. A single-trial generalized linear mixed model (GLMM) showed that pre‑response theta significantly predicted accuracy (*β* = 0.33, SE = 0.03, *z* = 11.86, *p* < 0.001, OR = 1.39, 95% CI [1.31, 1.46]). However, the slope of theta predicting accuracy was significantly lower in the OCD group than in the HC group (HC − OCD slope difference: *β* = 0.33, SE = 0.06, *z* = 6.00, *p* < 0.001, OR = 1.39, 95% CI [1.25, 1.55]). Importantly, these findings suggested that pre‑response theta activity remained behaviorally relevant in people with OCD, but unlike in HC participants, it was not selectively enhanced by motivational conflict before response execution. Full fixed‑effect estimates, confidence intervals, odds ratios, and model-fit statistics for these logistic mixed‑effects models were provided in S1 Table.

After showing that midfrontal theta in the OCD group reflected conflict detection, we next tested whether the conflict‑related signal was involved in reducing motivational biases in behavior. We extended our computational modeling framework and constructed a theta modulation model family (M4a–M4d) based on the winning behavioral model M3c [50,70]. Before incorporating trial-by-trial theta estimates into the computational models, we examined whether they were consistent with the trial‑averaged findings. Single-trial mixed‑effects analyses confirmed significant conflict‑related increases in midfrontal theta power in both groups (see Text D in S1 File) [2,44,49]. Trial‑by‑trial midfrontal theta power (*θ*_*t*_) was added to the model as a modulation signal, and the parameter *β* was used to test how theta acted on different components of the model. Specifically, these models tested whether midfrontal theta modulated the Pavlovian response tendencies (M4a), the instrumental contribution, reflecting the weighting of instrumental action values during choice (M4b), and/or the balance between the Pavlovian bias and instrumental contribution (M4c). M4d further tested whether theta modulated instrumental learning bias, that is, whether theta activity during decision-making influenced later learning updates. We then compared these models to determine how midfrontal theta modulated motivational biases in the OCD group.

As shown in Table 1 (M4a–M4d) and Fig 4D and 4F, this model family was designed to adjudicate between alternative computational mechanisms through which trial‑by‑trial midfrontal theta relates to biased action selection. In both groups, M4b, which used learned *Q* value as internal variable tracking instrumental action values and allowed theta power to scale their contribution to choice, provided the strongest model evidence (HC: WAIC = 18454.40, ΔWAIC = −131.38, *R*^2^ = 60.04%; OCD: WAIC = 24419.47, ΔWAIC = −124.49, *R*^2^ = 42.83%). Within M4b, the theta‑modulation parameter (*β*_*θ*‑power_) showed negative subject‑level posterior median estimates in both groups (HC: 89.19% of participants < 0; OCD: 72.22% < 0), with no between‑group difference (Δ_HC−OCD_ ≈ −0.21, *P*(Δ < 0) = 0.786). The winning model suggested that conflict‑related midfrontal theta signals primarily modulated motivational biases in action by increasing the weighting of instrumental action values during choice. Additionally, given that instrumental learning is updated following feedback, we examined feedback-locked midfrontal theta activity for evidence of modulation related to instrumental learning bias. Consistent with the model comparison, feedback-locked theta primarily reflected feedback outcome and showed no significant modulation associated with instrumental learning bias (see Text E and Figs D**–**E in S1 File) [71–73].

### Specific impairment of midfrontal network coupling is associated with ineffective modulation of motivational biases in OCD

In the OCD group, we found that motivationally incongruent trials induced a significant increase in midfrontal theta, and that stronger theta activity was associated with stronger instrumental contribution to choice, suggesting that conflict‑related control signals were still present. However, people with OCD still showed maladaptive behavior, suggesting that an increase in local midfrontal theta activity alone may not be sufficient for effective modulation of motivational biases. Moreover, compared with healthy controls, the increase in midfrontal theta did not mainly occur before the correct response in the OCD group, but instead overlapped more with the behavioral response. Therefore, we asked whether the core deficit in OCD lies not in generating conflict‑related control signals, but in ineffective transmission to downstream executive systems through network coupling. Previous studies have shown that increased theta phase synchrony between the midfrontal cortex and task‑relevant regions (i.e., lPFC and motor cortex) is associated with conflict processing [50,74]. We next examined whether motivational conflict affected ISPS between midfrontal channels and task‑related sites, in order to test whether delayed conflict‑related control in OCD influenced modulation of motivational biases at the network level. To prevent execution‑related motor activation from biasing synchrony estimates [44], analyses of motor targets focused on non‑executing sites (ipsilateral Go and NoGo). This approach minimized confounding from overt motor responses, allowing us to dissociate conflict‑related coupling from execution‑driven effects.

**Fig 5:**
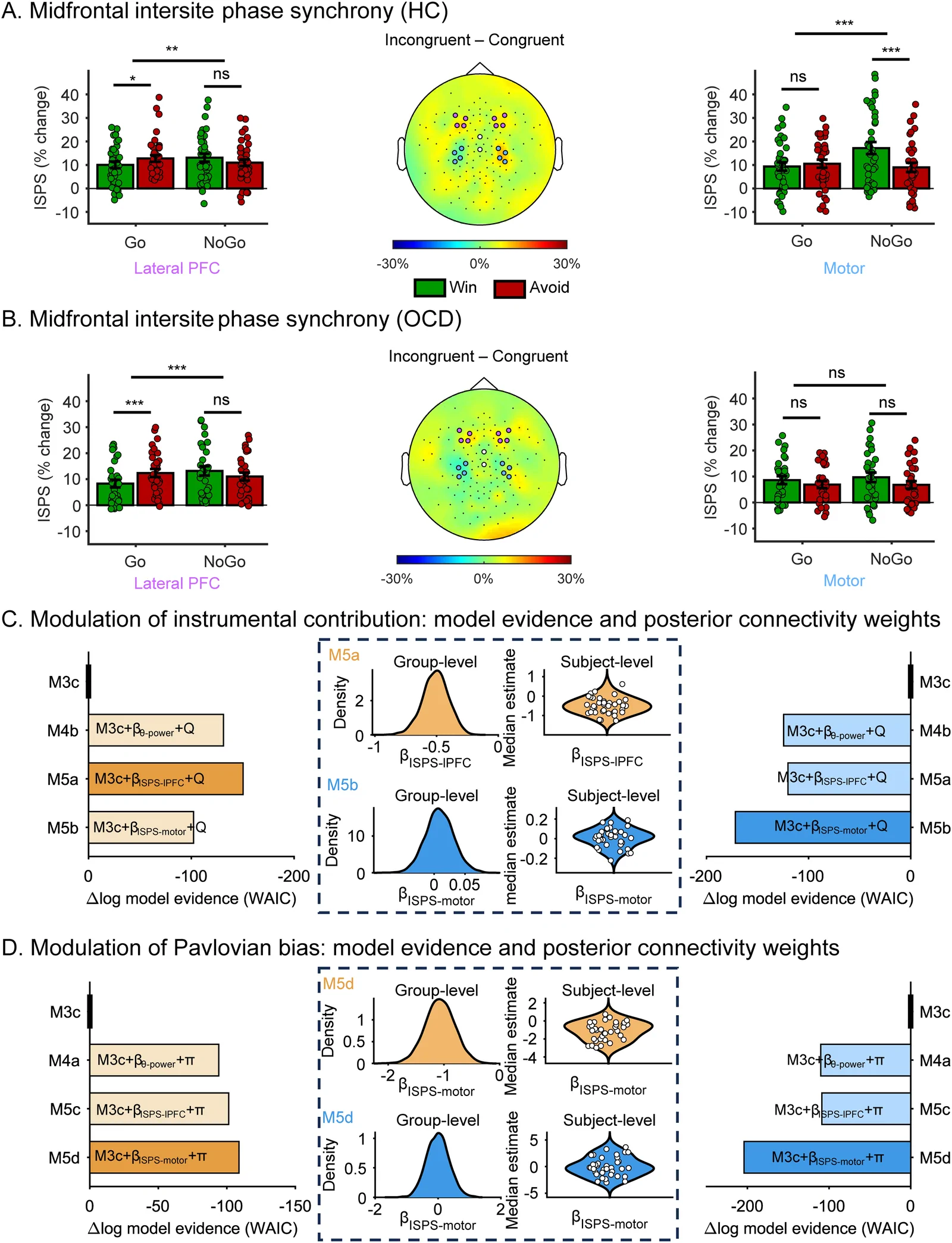
Conflict‑related modulation of midfrontal theta synchrony with lateral prefrontal and motor sites. **(A, B)** Conflict‑related midfrontal ISPS in HC **(A)** and OCD **(B)**. Left: Mean theta phase synchrony (ISPS; % change; incongruent minus congruent) between the midfrontal seed and lateral prefrontal target channels across the Go/NoGo × win/avoid conditions. Middle: Scalp topography of ISPS for incongruent relative to congruent trials (incongruent minus congruent). White discs indicate the midfrontal seed channels defined by the cue‑locked theta conflict effect, and purple and blue discs mark the lateral prefrontal and motor target channels, respectively. Right: Conflict‑related modulation of the midfrontal‑motor ISPS (here shown for the ipsilateral nonexecuting motor channels). **(C, D)** Model evidence for ISPS‑modulated parameters and posterior modulation weights in HC (left) and OCD (right). Bar plots show improvements in model evidence relative to the baseline behavioral model M3c (ΔWAIC; lower values indicate better fit). **(C)** Modulation of the instrumental contribution. Model evidence for synchrony‑based extensions is evaluated by comparing M5a (*β*_ISPS‑lPFC_) and M5b (*β*_ISPS‑motor_) against the theta‑power modulation model M4b, testing whether phase synchrony scales the contribution of instrumental action values to choice. Insets show posterior distributions of *β*_ISPS‑lPFC_ and *β*_ISPS‑motor_ from the best‑fitting model in each group (group‑level density on the left; participant‑specific medians on the right; upper = HC, lower = OCD). For the HC group, the best fit was M5a (*β*_ISPS‑lPFC_), whereas for the OCD group the best fit was M5b (*β*_ISPS‑motor_). Negative parameter estimates indicate that stronger synchrony is associated with increased weighting of instrumental action values. **(D)** Modulation of Pavlovian bias. Model evidence improves even further for models in which midfrontal‑lateral prefrontal (*β*_ISPS‑lPFC_; M5c) or midfrontal‑motor (*β*_ISPS‑motor_; M5d) phase synchrony scales the Pavlovian bias, compared with local midfrontal theta power scaling the Pavlovian bias (*β*_*θ*‑power_; M4a). Insets show posterior distributions of the corresponding synchrony modulation parameter from the best‑fitting model in each group (format as in **C)**. Parameter estimates are negative, indicating that stronger synchrony is associated with reduced Pavlovian bias. The numerical data underlying panels A–D can be found in S4 Data.

To examine conflict‑related network coordination, we extracted theta‑band ISPS values from correct trials within the significant cue‑locked theta window and used repeated-measures analyses of variance (ANOVAs) with valence (“win” versus “avoid” cues) and required action (Go versus NoGo) as within‑subject factors to test whether MFC‑lPFC and MFC‑motor phase synchrony increased for motivationally incongruent relative to congruent cues. As shown in Fig 5A and 5B, theta phase synchrony analyses showed reliable conflict‑related increases in the MFC‑lPFC phase synchrony in both groups (incongruent > congruent; HC: *F*(1,36) = 10.69, *p* = 0.002, *η*_*p*_^2^ = 0.23; OCD: *F*(1,29) = 27.32, *p* < 0.001, *η*_*p*_^2^ = 0.49). However, MFC‑motor phase synchrony differed between groups. HC participants showed increased conflict‑related phase synchrony between the MFC and nonexecuting motor sites (ipsilateral Go and bilateral NoGo responses; *F*(1,36) = 14.76, *p* < 0.001, *η*_*p*_^2^ = 0.29), consistent with inhibitory control over non‑target actions. In contrast, MFC‑nonexecuting motor phase synchrony did not differ between conflict and non‑conflict conditions in OCD (*F*(1,29) = 0.25, *p* = 0.621, *η*_*p*_^2^ = 0.01). Taken together, people with OCD showed no significant conflict‑related effect in phase synchrony between MFC and non‑executing motor regions, suggesting that network coordination related to non‑target action inhibition may be impaired during conflict.

The previous trial‑averaged results showed that the OCD group still had increased conflict‑related theta phase synchrony between MFC and lPFC, but lacked the significant conflict‑related phase synchrony effect between MFC and motor cortex that was observed in healthy controls. However, a key question was whether abnormalities in theta phase synchrony were associated with the modulation of motivational biases during conflict. Therefore, we built an ISPS Modulation Family of models (M5a–M5b) based on the theta modulation model of instrumental contribution (M4b). In these models, single‑trial theta phase synchrony replaced local theta power (*θ*_*t*_) as the modulation signal, allowing us to test whether MFC‑lPFC synchrony (ISPS_lPFC,t_) and MFC‑motor synchrony were involved in the modulation of instrumental contribution. Before fitting these models, we examined whether the trial‑by‑trial synchrony estimates reproduced the corresponding trial‑averaged effects. Single‑trial mixed‑effects analyses confirmed significant conflict‑related modulation of MFC‑lPFC synchrony in both groups, whereas the execution‑dependent conflict effect in MFC‑motor synchrony was evident in healthy controls but not in the OCD group (see Text F in S1 File) [34,44,75–78]. These supplementary results indicated that the trial‑by‑trial synchrony estimates captured the principal subject‑level patterns observed in the trial‑averaged analyses and were therefore suitable for subsequent computational modeling. In addition, the trial‑averaged statistical analyses mainly focused on ipsilateral motor regions in order to reduce motor‑related noise. In the computational models, however, we used contralateral motor signals because the contralateral motor cortex is more directly involved in behavioral output. This allowed us to test whether MFC‑motor synchrony (ISPS_motor_contra,t_) was associated with the modulation of motivational biases.

As shown in Table 1 (M5a–M5b), we tested whether theta phase synchrony provides greater explanatory power than local theta power in the modulation of motivational biases. In the models testing modulation of the instrumental contribution (Fig 5C, Table 1 M5a and M5b), adding single‑trial theta phase synchrony as the moderator improved model evidence relative to the corresponding theta‑power model. For HC participants, model comparison favored the MFC‑lPFC theta phase synchrony model (M5a; WAIC = 18435.32; ΔWAIC=−19.08; *R*^2^ = 60.07%) over M4b, and *β*_ISPS-lPFC_ was stably negative (89.19% of participants < 0), indicating that stronger MFC‑lPFC theta phase synchrony increased the weighting of instrumental action values. In contrast, in the OCD group, the best‑fitting model was the MFC‑motor theta phase synchrony model (M5b; WAIC = 24372.24; ΔWAIC = −47.23; *R*^2^ = 43.05%), but the group‑level modulation parameter was statistically negligible (*β*_ISPS‑motor_: mean = +0.009; 95% HDI [−0.038, 0.053]; Pr(*β* < 0) = 0.35), and the Bayes factor favored the absence of a directional effect (BF_10_ = 0.008). Thus, although MFC‑motor synchrony offered the best account of choice behavior in the OCD group, its group‑level influence on the weighting of instrumental action values was weak or heterogeneous rather than consistently directional.

Parameter estimates from the baseline model (M3c; Fig 3E) confirmed that people with OCD exhibited an increased Pavlovian bias. We next sought to determine whether this exaggerated Pavlovian bias was linked to abnormal modulation at the network level. Specifically, we examined whether functional coordination between midfrontal and task‑related regions was associated with the modulation of Pavlovian bias during motivational conflict, providing a more comprehensive network‑level account of OCD pathology. We fitted a parallel set of extended models in which ISPS was used as the modulation parameter. These models tested whether theta phase synchrony between MFC and lPFC (M5c) or motor cortex (M5d) modulated the Pavlovian bias parameter on a trial‑by‑trial basis as motivational conflict varied.

In the models with the Pavlovian bias parameter *π* (Fig 5D, Table 1 M5c and M5d), compared with M4a, the model in which the MFC‑motor phase synchrony scaled *π* (M5d) provided a better fit in both groups (HC: WAIC = 18476.93, ΔWAIC = −14.79, *R*^2^ = 60.07%; OCD: WAIC = 24339.93, ΔWAIC = −93.97, *R*^2^ = 43.13%). The synchrony‑scaling parameter (*β*_ISPS‑motor_) was negative (89.19% of participants < 0), indicating that stronger synchrony was associated with reduced Pavlovian bias in the HC group. Notably, the *β*_ISPS‑motor_ parameter estimate in the OCD group had a slightly negative posterior mean, but the posterior distribution showed no consistent directionality (mean = −0.005; 95% HDI [−0.708, 0.682]; Pr(*β* < 0) = 0.51; BF_10_ = 0.12). Although MFC‑motor phase synchrony improved model fit relative to theta power, it did not provide evidence for a reliable group‑level inhibitory effect on Pavlovian bias in the OCD group.

### Heterogeneous regulatory orientations of midfrontal‑motor coupling and their clinical relevance in OCD

Previous results showed that, in the OCD group, the MFC‑motor cortex theta phase synchrony model was the best‑fitting model for modulation of both the instrumental contribution (Fig 5C, right panel; M5b) and Pavlovian bias (Fig 5D, right panel; M5d). Although these two effects were estimated in separate models, they correspond to two theoretically complementary routes through which motivational conflict can be modulated by increasing the weighting of instrumental action values and suppressing Pavlovian response tendencies. However, the group‑level modulation effects did not show a consistent direction in those models. These results suggested that the underlying regulatory mechanism of phase synchrony was characterized by substantial inter‑individual heterogeneity. Consequently, we shifted from group‑averaged analyses to an exploratory individual‑level approach to examine how these two synchrony‑related modulation estimates were distributed across participants.

**Fig 6:**
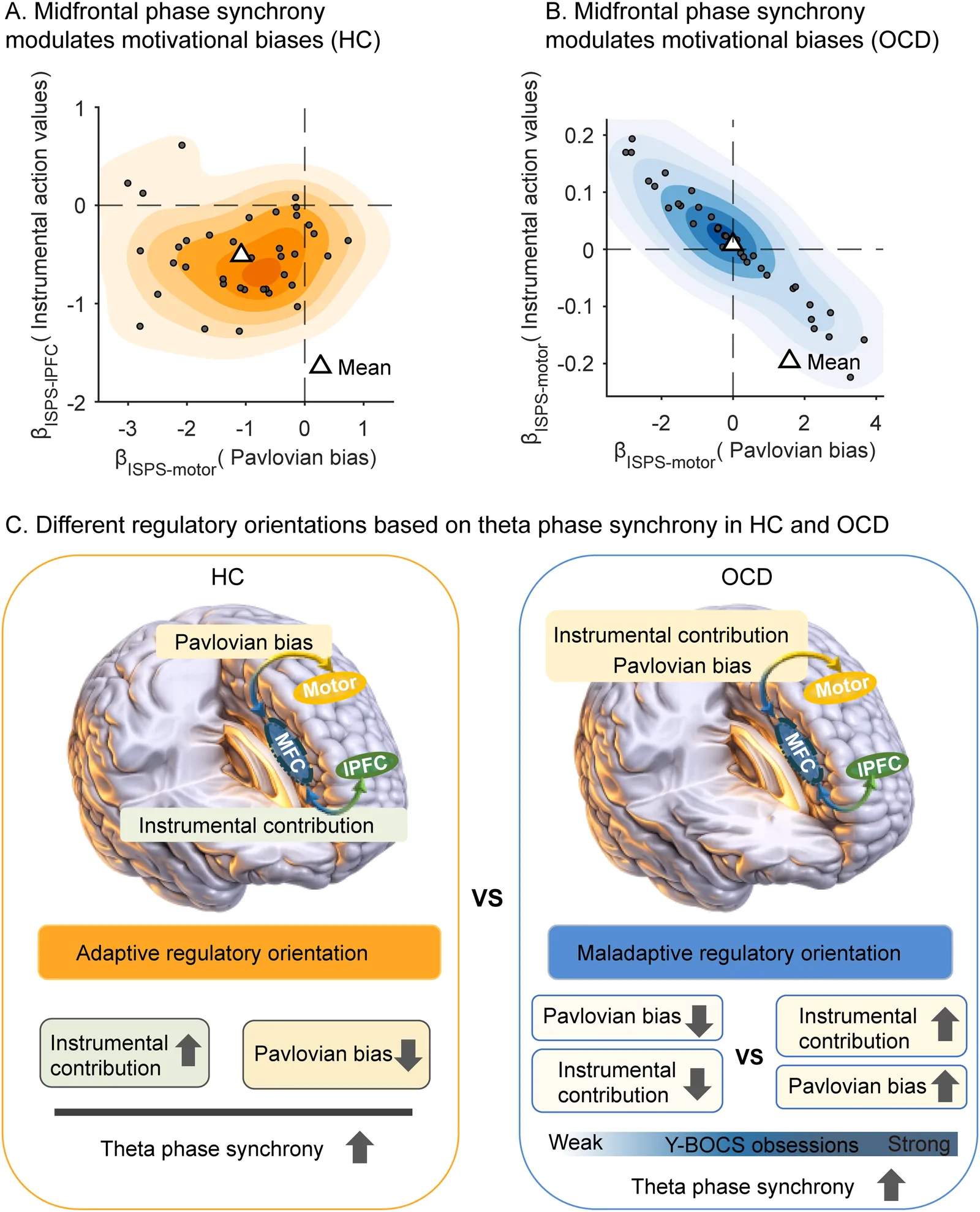
Subject‑level distributions of midfrontal theta phase‑synchrony modulatory parameters and their clinical relevance. **(A, B)** Descriptive visualization of individual regulatory orientation of modulation of motivational biases (A, HC; B, OCD). Each point represents an individual’s parameter estimates extracted from separate best‑fitting theta phase‑synchrony models (Fig 5C and 5D; instrumental contribution: M5a for HC and M5b for OCD; Pavlovian bias: M5d for both groups), plotted in a shared coordinate space to provide a descriptive visualization of individual regulatory orientation. For Pavlovian bias (x‑axis), both groups were best fit by the MFC‑motor synchrony model; the x‑axis therefore represents the MFC‑motor phase synchrony modulation parameter, *β*_ISPS‑motor_, indexing modulation of Pavlovian bias. For instrumental contribution (y‑axis), the best‑fitting model differed by group: HC were best fit by the MFC‑lPFC synchrony model (M5a), such that the y‑axis in panel A represents *β*_ISPS‑lPFC_ for instrumental contribution, whereas OCD were best fit by the MFC‑motor synchrony model (M5b), such that the y‑axis in panel B represents *β*_ISPS‑motor_ for instrumental contribution. For interpretation, *β* < 0 on the x‑axis indicates suppression of Pavlovian bias, whereas *β* < 0 on the y‑axis indicates increased weighting of instrumental action values during choice. Black dots indicate individual parameter estimates, background contours show the two‑dimensional kernel density distribution, white triangles mark the group mean, and dashed lines indicate the zero‑effect reference. **(C)** Schematic illustration of regulatory orientations and their clinical relevance. Left (HC): motivational‑bias modulation followed an adaptive complementary pattern, in which enhanced MFC‑lPFC theta phase synchrony was associated with increased weighting of instrumental action values during choice, whereas enhanced MFC‑motor theta phase synchrony was associated with suppression of Pavlovian bias. Right (OCD): modulation of motivational biases was dominated by the MFC‑motor theta phase synchrony pathway, representing a continuous trade‑off between two regulatory profiles rather than discrete subtypes. For descriptive purposes, the schematic illustrates the continuous distribution of these individual regulatory tendencies, which vary between an inhibitory prioritization profile where Pavlovian bias is suppressed at the cost of reduced instrumental weighting, and a value prioritization profile where increased instrumental weighting is accompanied by an amplified Pavlovian bias. The gradient bar at the bottom indicates increasing Yale‑Brown Obsessive Compulsive Scale (Y‑BOCS) obsessive-thought severity from left to right. Greater obsessive‑thought severity predicted a stronger tendency toward the value prioritization orientation. The numerical data underlying panels A and B can be found in S5 Data.

As shown in Fig 5C and 5D, we extracted the beta parameters from the winning synchrony models quantifying how theta phase synchrony scaled the weighting of instrumental action values and Pavlovian bias, and plotted them in a common coordinate space as a descriptive individual‑differences visualization of regulatory orientation (Fig 6A and 6B). When Pavlovian response tendencies conflicted with instrumental demands, healthy controls modulated motivational biases through a complementary pattern of theta phase synchrony between MFC and task‑relevant regions. In Fig 6A and 6C, most healthy controls fell into the lower‑left-hand quadrant, indicating stronger MFC‑lPFC theta phase synchrony associated with increased weighting of instrumental action values during choice, together with stronger MFC‑motor theta phase synchrony associated with inhibition of Pavlovian response tendencies. By contrast, in people with OCD, MFC‑motor theta phase synchrony emerged as the predominant pathway associated with modulation of motivational biases, while individual regulatory orientations showed a broader and more heterogeneous distribution within the regulatory-orientation space (Fig 6B and 6C). As shown in Fig 6B, people with OCD falling in the upper‑left quadrant tended to inhibit Pavlovian response tendencies at the cost of reduced instrumental weighting, whereas those falling in the lower‑right quadrant showed increased instrumental weighting accompanied by amplified Pavlovian response tendencies. This divergent distribution highlights marked inter‑individual heterogeneity in OCD, where opposing regulatory tendencies may have offset directional effects at the group level. To address the possibility that the separately estimated synchrony effects on instrumental weighting and Pavlovian bias might reflect overlapping variance, we additionally fitted joint models in which both modulation effects were estimated simultaneously within the same model likelihood. The jointly estimated parameters reproduced both the group‑specific regulatory‑orientation patterns and the association between regulatory orientation and obsessive‑thought severity observed in the primary analysis. These supplementary analyses supported the robustness of the individual-level regulatory‑orientation findings (see Text G and Figs F**–**G in S1 File).

To test whether this heterogeneity was clinically meaningful, we combined each OCD participant’s *β* estimates for scaling Pavlovian bias and instrumental contribution into a single angular index (Regulatory orientation angle, Φ_reg_). Importantly, Φ_reg_ was treated as a continuous measure of regulatory orientation rather than as a categorical subgroup label. The direction of the angle reflects the preference for modulating motivational biases by upregulating instrumental action values or suppressing Pavlovian bias. We found that Φ_reg_ was positively correlated with obsessive‑thought severity (Fig 6C, right), and this association remained significant after controlling for anxiety and depression (*r* = 0.38, *p* = 0.022), and after additionally controlling for trait impulsivity (*r* = 0.34, *p* = 0.042). These results indicated a clinically meaningful link between regulatory orientation and symptoms. Higher severity of obsessive thoughts was associated with a stronger tendency to prioritize increased instrumental contribution when modulating maladaptive biases under motivational conflict, but this tendency was accompanied by a simultaneous amplification of Pavlovian bias.

## Discussion

The present study delineates the computational and neural mechanisms underlying impaired modulation of motivational biases in OCD. In OCD, we found that both Pavlovian and instrumental learning mechanisms contributed to the motivational biasing of action, such that people with OCD exhibited heightened Pavlovian bias and lower learning rate than HC. On correct conflict trials, people with OCD showed increased midfrontal theta power, consistent with the pattern observed in HC, reflecting conflict detection and the generation of a control‑demand signal. We further examined theta phase synchrony between midfrontal and task‑relevant regions during motivational conflict. The results indicated a selective impairment in the engagement of task‑relevant networks by conflict‑related control signals. Specifically, MFC‑lPFC theta phase synchrony increased under conflict in OCD as well as in HC. In contrast, and unlike HC, MFC‑motor theta phase synchrony showed no reliable conflict‑related increase at the trial‑averaged level. Next, we investigated whether the abnormality in theta phase synchrony was associated with impaired modulation of motivational biases in OCD. The results showed that, although single‑trial modeling favored MFC‑motor theta phase synchrony as the best predictor of conflict‑related choice variability in OCD, its gain parameters showed no consistent direction at the group level, indicating marked heterogeneity in OCD. This heterogeneity suggested that people with OCD varied continuously in their regulatory orientation during the modulation of motivational biases, and this continuous variation was associated with the severity of obsessive thoughts. However, regardless of orientation, Pavlovian bias and instrumental contribution were modulated in parallel rather than in opposition, thereby preventing effective resolution of motivational conflict and promoting maladaptive choice. Overall, OCD appears to be characterized not by failure to recognize the need for control, but by unreliable coupling of control signals to motor implementation, contributing to the clinical manifestation of the “insight‑action dissociation”.

Behavioral modeling confirmed that motivational biases include dissociable Pavlovian and instrumental learning biases, corroborating previous research [50,79–81]. Additionally, these results showed that people with OCD exhibited elevated Pavlovian bias, but not instrumental learning bias. This pattern is broadly consistent with recent evidence that compulsivity preferentially affects the stochasticity of decision policy rather than reinforcement learning itself [82]. Heightened Pavlovian bias in OCD may reflect an excessive influence of cue valence on action selection, which becomes particularly maladaptive when Pavlovian response tendencies conflict with instrumental demands, thereby promoting suboptimal choices. This interpretation is consistent with prior evidence of ventral striatal hyperactivity [83,84] and hyper‑coupling within limbic networks [85] in OCD, which may provide a neural basis for heightened sensitivity to motivationally salient cues. Furthermore, the lower learning rate suggests that people with OCD can successfully learn the task rules, but they are still susceptible to interference when adjusting their choices based on feedback. This result reflects impaired flexible goal‑directed control [86–88], which is closely linked to reduced activation within the ventromedial prefrontal cortex, orbitofrontal cortex, and caudate nucleus [89–91]. Collectively, these results define a computational phenotype of maladaptive decision‑making, characterized by heightened cue‑driven response propensity and slower feedback‑driven learning, promoting rigid behavior under high motivational drive. The computational phenotype provides an algorithmic account of the “insight‑action dissociation” in OCD. People with OCD were able to learn and represent the basic task rules, but when Pavlovian biases conflicted with instrumental requirements, their choices remained strongly biased by rigid, cue‑driven Pavlovian tendencies.

Midfrontal theta power differentiated motivationally incongruent from congruent cues, and also tracked trial‑by‑trial conflict strength in both groups. This pattern is consistent with established roles of midfrontal theta in conflict detection [92–96], signaling cognitive control demands [61,97–99]. These findings suggest that people with OCD retain the capacity to detect conflict and to recruit a control‑demand signal that supports accurate responding. Specifically, we found that midfrontal theta modulated maladaptive motivational biases by increasing the weighting of instrumental action values during conflict. This result is consistent with prior work linking midfrontal theta to goal‑directed planning [100–102]. Therefore, preserved local conflict‑related midfrontal theta signaling may reflect a neural correlate of the preserved “insight” component of the dissociation. It demonstrates that people with OCD can detect motivational conflict and generate a control‑demand signal, consistent with the clinical observation that people with OCD possess explicit insight into the irrationality of their obsessions and compulsive behaviors.

Given that local midfrontal theta reflected preserved conflict detection, the critical question for understanding “insight‑action dissociation” shifts from whether control demands are registered to how this generated signal is communicated to downstream functional networks. We therefore examined long‑range oscillatory interactions between the MFC and task‑relevant regions to test whether impaired motivational‑bias regulation in OCD was associated with inefficient network‑level communication rather than impaired local conflict detection. According to the “Communication Through Coherence” (CTC) hypothesis [103], long‑range information exchange relies on rhythmic synchronization between neuronal populations, which can be quantified using oscillatory coherence [104–107]. Interregional theta phase synchrony plays an important role in cognitive control [108–112]. Within this framework, the MFC serves as a hub that detects control demands and achieves functional specialization by engaging theta phase synchrony with particular regions. Specifically, the lPFC theta activity preferentially maintains abstract task rules and goal representations [113,114], supporting top‑down control [115,116]. Conversely, theta activity in the motor cortex is directly involved in modulating specific motor responses [117,118]. Therefore, we propose a sequential control model mediated by theta phase synchrony. Once conflict is detected by the MFC, control signals are relayed via MFC‑lPFC theta phase synchrony to reinforce goal‑directed rules and via MFC‑motor phase synchrony to gate action execution.

Under motivational conflict, people with OCD showed significantly increased MFC‑lPFC theta phase synchrony, suggesting that conflict‑ and control‑demand signals generated in the MFC can be effectively communicated to the lPFC. However, computational model comparisons indicated that, unlike HC, the MFC‑lPFC pathway did not provide the best account of the modulation of maladaptive biases, and thus the enhanced MFC‑lPFC coupling may reflect inefficient and compensatory recruitment in OCD [119]. Although the lPFC appears to receive conflict‑related signals from the MFC in both HC and OCD, it did not effectively modulate Pavlovian bias or instrumental contribution in the OCD group. This pattern is consistent with impaired goal‑directed control in OCD [20–22] and may help explain the reduced learning rate.

Furthermore, people with OCD showed more critical and variable abnormalities in the MFC‑motor pathway than HC. They did not show a reliable conflict‑related increase in synchrony between the MFC and nonexecuting motor sites, suggesting weaker inhibitory coupling of non‑target action channels during conflict. This result implies that even when the MFC generates a conflict‑related control‑demand signal, the signal is not consistently transmitted to mechanisms that inhibit competing responses in OCD, which makes it harder to support task‑required actions [48]. In the OCD group, reduced phase synchrony between the MFC and motor areas impairs the transmission of top‑down control signals to the motor cortex for action execution [120]. Other research has found local motor‑area abnormalities in OCD, such as glutamate‑related hyperexcitability in the supplementary motor area, which may reduce sensitivity to prefrontal regulatory input [121, 122]. Together, disrupted local excitability and weakened long‑range coupling may undermine rhythmic coordination and inhibitory control, providing a network‑level account of the “insight‑action dissociation”, which explains why compulsive behaviors still persist even when cognitive capacity remains intact.

We observed a critical divergence in the temporal relationship between conflict‑related theta enhancement and response execution across the participant groups. In HC participants, conflict‑related theta increased before the response and included a clear pre‑response component, which can support task‑required responding by adjusting decision thresholds [123] and suppressing automatic tendencies [124, 125]. In OCD, theta enhancement partly overlapped the response window and showed no reliable pre‑response component. Single‑trial analyses showed that pre‑response theta significantly predicted accuracy. However, the OCD group did not show the conflict‑specific pre‑response theta enhancement observed in the HC group. This pattern suggests that people with OCD retained behaviorally relevant pre‑response theta activity, but this signal was less flexibly scaled to conflict demands before action initiation, consistent with previous accounts of weakened proactive control before responding in OCD [126, 127]. Thus, under motivational conflict, control‑related theta activity in OCD may be less effectively organized before the response and instead become more evident closer to motor execution. Consistent with this interpretation, single‑trial modeling showed that MFC‑motor theta phase synchrony provided the best explanation of choice variability under conflict in OCD, both when modulating Pavlovian bias and when modulating the weighting of instrumental action values during choice.

Although the MFC‑motor synchrony model provided the best fit in OCD, the group‑level gain parameters showed no consistent direction, suggesting marked heterogeneity in how this pathway modulates behavior across people with OCD. Individual differences analyses supported this interpretation and characterized this variance as a continuous regulatory‑orientation space rather than as indicating discrete subtypes. Within this continuous regulatory‑orientation space, people with OCD falling within the inhibitory‑prioritization region showed a tendency to suppress Pavlovian bias during conflict, but this tendency was accompanied by reduced instrumental value weighting. Conversely, people with OCD falling within the value‑prioritization region showed a stronger instrumental contribution to choice during conflict, but this tendency was accompanied by simultaneous amplification of Pavlovian bias, which may have contributed to difficulty suppressing repetitive actions in high‑conflict situations. Rather than grouping people with OCD into discrete categories, our continuous angular metric (Φ_reg_) captured clinically meaningful variation in regulatory orientation, as reflected by its significant association with obsessive‑thought severity. Greater obsessive‑thought severity was associated with a stronger tendency to recruit MFC‑motor theta phase synchrony under motivational conflict to increase instrumental contribution. However, this pattern was accompanied by a simultaneous amplification of Pavlovian bias. Recent research found that people with OCD with high levels of obsessive thoughts had greater difficulty in evidence integration and were less able to use new evidence [128]. This finding may help explain the value prioritization orientation observed in the present study. People with OCD with higher obsession severity show a reduced ability to use incoming evidence to update their choices adaptively. Consequently, when faced with motivational conflict, they may compensate by increasing the weighting of instrumental action values or relying more on cue values to guide decisions, which may lead to rigid and maladaptive behavioral patterns.

While the present findings provide new insight into the neural regulation of motivational biases in OCD, several limitations should be acknowledged and may guide future research. First, the relatively small sample size imposes certain statistical and clinical constraints. For the exploratory correlations, the limited statistical power increases the risk of Type I errors, and therefore the results need to be interpreted cautiously. Clinically, the sample size also restricted our ability to implement formal split‑sample validation or to evaluate whether these neural regulatory patterns vary across specific OCD symptom dimensions, such as checking versus washing. The regulatory‑orientation analysis was intended to be a descriptive individual‑differences analysis rather than a formal subgroup classification. We therefore did not apply clustering or mixture modeling to identify statistically separable OCD subgroups. Second, we did not acquire magnetic resonance imaging data, which restricted our ability to identify the anatomical locations of the sources and targets of the observed effects beyond the scalp‑level lPFC and motor sites. Future studies with larger and more clinically diverse samples should employ formal clustering or mixture‑modeling approaches to classify people with OCD based on multidimensional symptom profiles, such as obsessive thoughts, checking, washing, and compulsive symptoms. This would allow researchers to test whether symptom‑based subgroups differ in their regulatory tendencies during motivational conflict. Such work would further clarify the mechanisms underlying OCD heterogeneity and may help inform more personalized interventions. Furthermore, future studies should incorporate multimodal imaging to improve anatomical specificity, and evaluate neuromodulation approaches to test directional and potentially causal hypotheses linking neural regulation to symptom expression. It would also be valuable to assess neuromodulation techniques designed to restore normal MFC‑motor rhythmic synchrony. Such approaches could support the development of more personalized and effective interventions.

In sum, under motivational conflict, people with OCD as well as healthy controls were able to detect conflict in the midfrontal cortex and generate control‑demand signals, which may be a mechanism supporting insight into optimal behavior. However, in people with OCD, this conflict‑related process appeared more prolonged and extended into the response window. This temporal extension shifted the expression of conflict modulation closer to the motor stage. In parallel, MFC‑motor theta phase synchrony provided the strongest model evidence for the modulation of maladaptive biases, yet this pathway showed a specific deficit under conflict. Together, these results imply that people with OCD could not effectively gate motor output or correct cue‑driven impulses at the action level, offering a mechanistic account of the “insight‑action dissociation” in OCD, in which preserved conflict awareness is decoupled from effective action implementation. These results suggest that research into the use of neuromodulation to treat OCD might benefit from shifting away from targeting localized activity [129, 130] to targeting long‑range rhythmic synchrony across control networks [131]. MFC‑motor synchrony represents a promising candidate pathway that may contribute to the future development of circuit‑based interventions.

## Materials and methods

### Ethics statement

The study was conducted in accordance with the principles expressed in the Declaration of Helsinki and was approved by the Ethics Committee of the School of Psychology, South China Normal University (approval number: SCNU‑PSY‑2022‑343). All participants were right‑handed and provided verbal informed consent prior to participation.

### Participants

Sample size estimation was conducted using G*Power 3.1.9.7 [132]. Because exact power estimation for the mixed‑effects models, permutation-based EEG analyses, and hierarchical computational models used in the present study is not directly implemented in G*Power, power was approximated using the corresponding subject‑level repeated‑measures designs. For the primary within‑participant valence × required action effect, a repeated-measures design with four measurements indicated that 36 participants were required to achieve 95% power to detect a medium effect (*f* = 0.25, *α* = 0.05), assuming a correlation of 0.50 among repeated measures and *ε* = 1. Both groups met this requirement (37 healthy controls and 36 people with OCD). For the primary group × valence × required action interaction, a two‑group repeated‑measures within‑between interaction indicated that 62 participants were required to achieve 99% power under a more stringent *α* = 0.01, assuming the same effect size (*f* = 0.25), correlation among repeated measures (0.50), and *ε* = 1. For the secondary individual-difference analyses examining associations between regulatory orientation and Y‑BOCS obsessive-thought severity within the OCD group, a correlation‑based a priori power analysis indicated that 32 participants were required to detect a large correlation (*ρ* = 0.50) with 85% power at *α* = 0.05 using a two‑tailed test.

We recruited 40 people with OCD from the Affiliated Kangning Hospital of Ningbo University and 40 healthy controls matched to the OCD group on age, sex, and years of education. After excluding seven participants (HC = 3, OCD = 4) with excessive artifacts in EEG preprocessing, data from 36 participants with OCD and 37 healthy controls were included in subsequent analysis. All participants were right‑handed and provided verbal informed consent prior to participation. The study was approved by the Ethics Committee of the School of Psychology, South China Normal University (approval number: SCNU‑PSY‑2022‑343) and was conducted in accordance with the Declaration of Helsinki.

For the OCD group, inclusion criteria were: (1) a primary diagnosis of obsessive‑compulsive disorder confirmed by licensed psychiatrists according to the Chinese version of DSM-5; (2) a Yale-Brown Obsessive Compulsive Scale total score ≥ 16; and (3) age between 18 and 50 years. Participants with any current or lifetime psychiatric diagnosis other than OCD were excluded, including depressive disorders such as major depressive disorder, anxiety disorders such as generalized anxiety disorder, substance use disorders, bipolar disorder, post‑traumatic stress disorder, and personality disorders. Additional exclusion criteria were a history of brain injury or any neurological disorder and receipt of electroconvulsive therapy within the three months prior to enrollment. Thus, everyone included in the final OCD sample had OCD as the primary diagnosis and did not meet criteria for current or lifetime psychiatric comorbidities. Furthermore, those in the OCD group were not medication‑free at the time of testing and remained on stable psychotropic medication during the experiment. Medications included selective serotonin reuptake inhibitors (SSRIs; *n* = 23), serotonin-norepinephrine reuptake inhibitors (SNRIs; *n* = 1), atypical antipsychotics (*n* = 8), benzodiazepines (BZDs; *n* = 5), non-benzodiazepine hypnotics/anxiolytics (*n* = 2), and antimanic agents (*n* = 1). Medication categories were not mutually exclusive, and some people with OCD received more than one class of drug. HC participants had no current or lifetime psychiatric disorder and no first‑degree family history of OCD. For the HC group, additional inclusion criteria were age between 18 and 50 years, and exclusion criteria were a history of brain injury or any neurological disorder and use of any psychotropic medication within the three months prior to study participation. Demographic characteristics and clinical scores appear in Table 2.

**Table 2:** Descriptive statistics for demographic variables and clinical measures.

Variable	OCD (*n* = 36)		HC (*n* = 37)		*t*/*χ*^2^
	Mean (SD)	Range (Min–Max)	Mean (SD)	Range (Min–Max)	
**Demographics**					
Sex, male (%)	75%	—	68%	—	0.49
Age, years	25.11 (3.53)	18–43	23.62 (6.05)	20–36	1.29
Education	15.25 (2.30)	12–22	16.05 (1.49)	13–19	1.77
**OCD symptoms**					
Y‑BOCS^a^	22.39 (5.15)	16–36	1.81 (2.89)	0–11	21.12^***^
Y‑BOCS obsessions	11.03 (3.18)	4–20	0.62 (1.59)	0–7	17.74^***^
Y‑BOCS compulsions	11.36 (2.53)	5–16	1.19 (2.33)	0–11	17.87^***^
OCI‑R^b^	23.11 (10.81)	5–52	11.38 (6.36)	1–24	5.67^***^
OBQ‑44^c^					
Responsibility/ Threat Estimation, RT	61.61 (17.14)	28–88	50.78 (13.78)	27–85	2.98^**^
Perfectionism/ Certainty, PC	58.33 (13.71)	28–80	49.51 (15.41)	25–85	2.58^*^
Importance/ Control of Thoughts, ICT	35.25 (12.87)	12–59	27.84 (10.94)	12–55	2.65^*^
STAI^d^	46.96 (7.27)	28.50–63	38.62 (10.36)	22–65	3.97^***^
STAI‑State	45.42 (7.86)	26–64	36.68 (10.78)	20–61	3.95^***^
STAI‑Trait	48.50 (8.36)	30–62	40.57 (10.48)	24–69	3.57^***^
BDI‑II^e^	11.08 (9.74)	0–43	6.76 (7.33)	0–34	2.15^*^
BAI^f^	29.11 (8.98)	0–54	24.84 (3.56)	21–34	2.69^*^
BIS‑11^g^	44.05 (11.89)	13.33–65	35.00 (9.47)	9.17–50	3.60^***^

Significance markers are reproduced as in the original table: * *p* < 0.05, ** *p* < 0.01, *** *p* < 0.001.

^a^Y‑BOCS (Yale‑Brown Obsessive Compulsive Scale): a measure of the severity of obsessive–compulsive symptoms. Total scores are categorized as 1–7 subclinical, 8–15 mild, 16–23 moderate, 24–31 severe, and 32–40 extreme. The scale includes two subscales, Obsessions (Y‑BOCS‑O) and Compulsions (Y‑BOCS‑C), each ranging from 0 to 20.

^b^OCI‑R (Obsessive‑Compulsive Inventory‑Revised): a measure of obsessive–compulsive symptom dimensions. It covers six domains: washing, obsessing, hoarding, checking, neutralizing, and ordering. The total score ranges from 0 to 72.

^c^OBQ‑44 (Obsessive Beliefs Questionnaire‑44): a measure of core cognitive beliefs associated with OCD. It includes three subscales measuring beliefs about responsibility/threat estimation (16–112), perfectionism/certainty (16–112), and importance/control of thoughts (12–84).

^d^STAI (State‑Trait Anxiety Inventory): a measure of state anxiety (STAI‑S) and trait anxiety (STAI‑T). For each, scores of 20–37 indicate no or low anxiety, 38–44 indicate moderate anxiety, and 45–80 indicate high anxiety.

^e^BDI‑Ⅱ (Beck Depression Inventory): a measure of depressive symptoms. Total scores are categorized as 0–13 minimal/no depression, 14–19 mild, 20–28 moderate, and 29–63 severe depression.

^f^BAI (Beck Anxiety Inventory): a measure of anxiety symptoms. Total scores are categorized as 0–7 minimal/no anxiety, 8–15 mild, 16–25 moderate, and 26–63 severe anxiety.

^g^BIS‑11 (Barratt Impulsiveness Scale‑11): a measure of three impulsivity dimensions: attentional impulsivity, motor impulsivity, and non‑planning impulsivity. The total score ranges from 0 to 100, with higher scores indicating greater impulsivity.

### Motivational Go/NoGo learning task

To dissociate Pavlovian response tendencies from instrumental learning processes, we employed a motivational Go/NoGo learning task [50]. In this task, participants learned by trial and error to make optimal responses to different cues in order to maximize rewards for win cues and minimize punishments for avoid cues. As illustrated in Fig 1, the learning phase used eight gem‑shaped cues as stimuli, each of which was presented 60 times. Each trial began with a cue presented for 1,300 ms followed by a fixation cross for 700 ms. Feedback was then displayed for 1,000 ms based on the participant’s performance. Each trial concluded with an inter‑trial interval ranging from 1,000–1,750 ms in steps of 250 ms. After learning, participants completed a forced‑choice transfer task in which pairs of previously learned cues were presented in counterbalanced positions (upper and lower) on the screen and they selected the cue they believed was more likely to obtain reward. Cues were always paired across categories (i.e., Go‑to‑Win/Go‑to‑Avoid/NoGo‑to‑Win/NoGo‑to‑Avoid), and there were 48 trials in total. Behavioral results of the forced-choice transfer task confirmed successful learning of cue valence and revealed a systematic preference for Go over NoGo cues that could not be explained by differences in cumulative outcomes, indicating enhanced subjective valuation of Go relative to NoGo cues (see Text A in S1 File).

### EEG data acquisition

While participants performed the task, EEG data were recorded using a 128‑channel EGI system (GSN‑HydroCel‑128; Electrical Geodesics, Eugene, OR, USA) and Net Station software. The online reference was set to Cz. Continuous EEG was recorded at a sampling rate of 500 Hz, with all electrode impedances maintained below 50 kΩ.

### EEG preprocessing

All preprocessing was conducted in MATLAB (version R2022b) [133] and EEGLAB (version 2022.0) [134]. Continuous EEG data were first filtered with a 0.5 Hz high‑pass filter and a 50 Hz low‑pass filter, and were then re‑referenced offline to the weighted average of the bilateral mastoids while restoring the reference electrode signal. The data were epoched into segments starting 1.75 s before cue onset and ending 1.5 s after feedback offset, and baseline‑corrected using the 200 ms pre‑cue interval. These relatively long epochs were used to minimize edge artifacts during subsequent time‑frequency decomposition. The epoched data were then visually inspected for trial rejection. Trials were rejected if they contained electromyographic activity or other non‑neural artifacts unrelated to brain activity, whereas trials containing eye blinks were retained for subsequent independent component analysis (ICA) correction. ICA was subsequently performed using EEGLAB’s runica algorithm in extended mode, with dimensionality reduced to 64 components using principal component analysis (PCA). Components related to blinks, eye movements, muscle activity, or other artifacts clearly distinguishable from brain activity were manually identified and removed. Across participants, 4–17 components were removed, with an average of 9 components removed per participant. After ICA, bad channels were identified by visual inspection and repaired using spherical spline interpolation. The number of interpolated channels ranged from 2 to 11 per participant, with an average of 5 interpolated channels. The cleaned EEG data were then spatially filtered using surface Laplacian estimation to reduce volume‑conduction effects and emphasize local cortical activity [135,136]. This procedure reduces spurious connectivity driven by spatial smearing and thereby improves the reliability of subsequent intersite phase synchrony estimates [137].

Participants were excluded from EEG analyses if more than 35% of their trials were rejected because of excessive artifacts. Based on this criterion, seven participants were excluded, including three healthy controls and four people with OCD. Among the retained participants, the proportion of rejected trials ranged from 0.47% to 33.96%, with a mean rejection rate of 6.97%. The final EEG analyses therefore included 37 healthy controls and 36 people with OCD. The task employed a 2 × 2 factorial design crossing valence (“win” versus “avoid” cues) and required action (Go versus NoGo), yielding four experimental conditions. For the retained participants, the analyzed trial counts across the four conditions were Go‑to‑Win (85.40 ± 20.09), NoGo‑to‑Avoid (83.00 ± 17.41), NoGo‑to‑Win (70.22 ± 26.76), and Go‑to‑Avoid (74.10 ± 21.22). These trial counts indicate that EEG and computational variables were estimated from adequate numbers of observations across conditions.

### Time–frequency decomposition

Time–frequency decomposition for cue‑ and response‑locked EEG data was conducted in MATLAB with complex Morlet wavelets from the FieldTrip toolbox (version 20211209) [138]. Specifically, the time series were convolved with Morlet wavelets, which can be conceptualized as sinusoidal carriers windowed by a Gaussian function and implemented as multiplication in the frequency domain. Frequencies ranged from 1 to 50 Hz in 39 logarithmically spaced steps, with the Gaussian width fixed at 4 cycles. Wavelet convolution yielded complex‑valued signals from which power and phase were extracted at each time‑frequency point and then down‑sampled to 40 Hz. For each condition, power values were averaged across trials and converted to decibel units using a condition‑averaged baseline interval from −250 to −50 ms relative to cue onset. Based on our hypotheses, we focused on midfrontal (Fz, FCz, Cz) theta‑band activity (4–8 Hz). Cue‑locked theta enhancement was defined as the time window after cue onset in which midfrontal theta power was stronger for incongruent than congruent trials. To identify the time window, we employed a time‑based permutation test (500 permutations) on cue‑locked, trial‑averaged midfrontal theta power, with congruency (congruent versus incongruent) as the within‑subject factor. Congruent trials included Go‑to‑Win and NoGo‑to‑Avoid cues, whereas incongruent trials included NoGo‑to‑Win and Go‑to‑Avoid cues. The significant time window identified by this test was then used as the theta extraction window. The resulting group‑specific windows were 400–650 ms in the HC group and 424–800 ms in the OCD group.

For the single-trial analyses used in the computational models, Laplacian‑filtered EEG data were first broadband filtered in the theta range (4–8 Hz) and Hilbert transformed. This yielded power time series, which were *z*-transformed and then averaged within the corresponding group-specific time windows. For the channels of interest, values were t‑weighted based on the main contrast (incongruent minus congruent). Mean theta power was then extracted from single‑trial data within the corresponding group‑specific window and used as a trial‑level EEG predictor in the computational models. The resulting power values were inverse-transformed before being entered into the computational models.

ISPS was computed from theta‑band phase angles to quantify phase coupling between the midfrontal seed cluster and two target clusters. Phase angles (*φ*) were used to compute intersite phase synchrony, which is taken to reflect functional connectivity between electrode sites [103,139,140]. In the theta band (4–8 Hz), ISPS was calculated at each time‑frequency point according to the following formula:


$$\begin{array}{c} \text{ISPS}=|\frac{\text{1}}{N}\sum_{n=\text{1}}^{N}{\text{e}}^{\text{i}}\varphi_{j,n,t,f}-\varphi_{k,n,t,f}| \end{array}$$ (1)

Here, *N* is the number of trials, *i* is the imaginary unit, and *j* and *k* index the seed and target electrodes, respectively. ISPS values range from 0, indicating no phase synchrony, to 1, indicating a consistent phase difference across trials. The lateral prefrontal target cluster comprised bilateral frontal electrodes approximately corresponding to AF3/F3/F5/AF7 and AF4/F4/F6/AF8, whereas the motor target cluster comprised bilateral central electrodes approximately corresponding to C3/CP3 and C4/CP4. Because ISPS is sensitive to trial number, an equal number of trials was randomly selected from each condition before ISPS computation. This procedure was repeated 100 times, and ISPS values were averaged across repetitions. Condition cells with fewer than 20 trials were excluded, resulting in the exclusion of six participants from the OCD group for the ISPS analysis. ISPS values were baseline‑transformed using the −250 to −50 ms pre‑cue interval. Specifically, ISPS values were converted to percent signal change relative to the same condition-averaged baseline interval.

For the single-trial ISPS analyses used in the computational models, phase angles from the Hilbert-transformed theta-band signals were extracted to compute phase synchrony between the midfrontal seed electrodes and the lateral prefrontal and motor target electrodes within the same group-specific time windows as follows:


$$\begin{array}{c} \text{ISPS}=\left|\frac{\text{1}}{T}\sum_{t=\text{1}}^{T}{\text{e}}^{\text{i}}\varphi_{j,n,t}-\varphi_{k,n,t}\right| \end{array}$$ (2)

Here, *T* denotes the number of time points within the analysis window, and *t* indexes time points.

The seed electrodes were weighted in the same way as the power time series, whereas the target electrodes were *t*‑weighted according to the corresponding main ISPS contrast (lateral prefrontal: valence × required action; motor channels: congruency × motor execution). The resulting power and ISPS values were inverse‑transformed before being entered into the computational models.

In all analyses, we first defined electrode clusters of interest based on previous studies and then refined these clusters in a data‑driven manner, in a way that was orthogonal to the main contrasts of interest, because scalp potential topographies can vary substantially across participants and studies. To assess midfrontal theta power, we initially selected two central electrodes approximately corresponding to Cz and FCz in the 10–20 system, which have been shown in prior work to be particularly sensitive to conflict‑related midfrontal theta activity [136,140]. The midfrontal cluster was then refined using condition-averaged cue-locked data, which were orthogonal to the main contrast, by adding an additional frontal electrode approximately corresponding to Fz, yielding the final Fz/FCz/Cz midfrontal seed cluster. Post hoc t tests were used to determine which electrodes contributed significantly to the group‑level cluster effect, and these significant electrodes were t‑weighted according to the main contrast (incongruent minus congruent). T‑weighting was applied only after the effect of interest had been established at the group level and therefore could not bias identification of the effect. Performing t‑weighting at the group rather than individual level also reduced susceptibility to noise and improved the generalizability of the resulting trial‑wise measures.

For the connectivity analyses, a t‑weighted midfrontal seed time series was constructed from these significant midfrontal electrodes. The lateral prefrontal target cluster was selected to include dorsolateral peak electrodes observed in the condition‑averaged cue‑locked data and to extend laterally to sites near F5/F6, which are traditionally regarded as lateral prefrontal electrodes [141,142]. The motor target cluster was selected based on clear hand-related lateralization independent of cue valence.

### Statistical analyses

Behavioral data were analyzed using mixed‑effects models in R (version 4.4.3) [143] with the lme4 (version 1.1-37) [144], psych (version 2.5.6) [145], and lmerTest (version 3.1−3) [146] packages. To examine the effects of motivational valence on behavioral activation and potential group differences, generalized linear mixed models (GLMMs) were first used to analyze choice preference (Go versus NoGo), with valence (“win” versus “avoid” cues) and required action (Go versus NoGo) as within‑subject factors. Group (HC versus OCD) was added as a between‑subject factor to assess whether the influence of motivational valence on behavioral activation differed between healthy controls and people with OCD. Second, to determine whether the effects on Go responding were explained by correct or incorrect Go responses, additional analyses were restricted to Go cues. In these analyses, valence (“win” versus “avoid” cues) was the within-subject predictor, and Group (HC versus OCD) was included as a between‑subject factor to examine group differences in the observed effects. All models included the full set of relevant main effects and interactions and implemented a maximal random‑effects structure. Unless otherwise specified, all inferential statistical tests were two‑sided, with *α* = 0.05. Exact *p* values were reported for *p* ≥ 0.001, whereas smaller values were reported as *p* < 0.001.

For the EEG analysis, we employed a cue‑locked time‑based permutation test (500 permutations) to identify a time window in which midfrontal theta power (4–8 Hz) differed between congruent and incongruent trials for the OCD and HC groups, separately. Go‑to‑Win and NoGo‑to‑Avoid cues were classified as motivationally congruent because the Pavlovian response tendencies aligned with the instrumental requirements, whereas NoGo‑to‑Win and Go‑to‑Avoid cues were classified as motivationally incongruent. Post hoc *t*‑tests were subsequently conducted to determine which of the three midfrontal channels contributed significantly to the time window identified by the permutation test (Bonferroni‑corrected *α* = 0.017). Considering that (ⅰ) midfrontal theta power increases significantly after errors [147,148], and (ⅱ) errors occur more frequently on motivationally incongruent than congruent trials, we restricted this analysis to correct trials to minimize the influence of error processing on the midfrontal theta signal. Error trials were examined separately, and the corresponding results were reported in Text D in S1 File.

To assess the behavioral relevance of pre-response theta, we tested whether trial‑level theta power predicted response accuracy. Because both correct and error responses were required, response‑locked permutation analyses were repeated across all Go trials. A significant pre‑response theta window was identified in the HC group (−536 to −274 ms), whereas no significant cluster was found in the OCD group. An exploratory interval showing descriptively greater theta for Go‑to‑Avoid than Go‑to‑Win trials was therefore used for the OCD group (−655 to −324 ms). Within these intervals, midfrontal theta power (4–8 Hz) was averaged and standardized within participant. Accuracy (correct = 1, incorrect = 0) was analyzed using a binomial GLMM with pre‑response theta, group, and their interaction as fixed effects, trial-level RT and trial order as covariates, and participant as a random intercept.

Separately, to characterize interregional coordination during cue processing, we examined theta‑band phase synchronization between the MFC and target regions within the group‑specific cue‑locked time windows identified by the permutation tests (HC: 400–650 ms; OCD: 424–800 ms). Mean midfrontal theta power was extracted from these windows, and ISPS values between the MFC and target regions (lPFC and motor cortices) were calculated on correct trials. Repeated‑measures ANOVAs with valence (“win” versus “avoid” cues) and required action (Go versus NoGo) as within‑subject factors were conducted in IBM SPSS Statistics (version 22.0.0.0) [149] to evaluate conflict effects on theta power and ISPS. For the motor ISPS analysis, we further examined whether ISPS differed between the executing motor cortex (contralateral to the Go response hand) and the nonexecuting motor cortex (ipsilateral to the Go response hand). Because midfrontal functional connectivity is generally considered to be activity‑dependent [44], such that target regions become more sensitive to midfrontal signals when they are more strongly activated, Go responses were separated into contralateral and ipsilateral responses. This yielded three levels of the required response factor (Go_contra, Go_ipsi, and NoGo_bilateral). Significant valence × required response interactions were followed up with simple‑effects analyses comparing win and avoid conditions separately within each level of required response. For within‑subject factors with more than two levels, sphericity was assessed using Mauchly’s test, and Greenhouse–Geisser corrections were applied when this assumption was violated.

### Computational models

We modeled trial‑by‑trial choices in the motivational Go/NoGo learning task within a hierarchical Bayesian reinforcement‑learning framework [50]. In this framework, nested reinforcement‑learning models were used to formally test whether Pavlovian response biases and instrumental learning biases contributed to asymmetric effects of valence on behavioral activation. Individual‑level parameters characterizing learning, choice stochasticity, and motivational biases were estimated while being constrained by group‑level population distributions. This hierarchical structure allowed partial pooling across participants, thereby improving the stability of subject‑level parameter estimates while preserving inter‑individual variability. Consistent with recommended practices in computational cognitive modeling [150,151], we specified theory‑driven candidate models, compared competing model families, inspected convergence diagnostics, and evaluated model adequacy using posterior predictive simulations.

On each trial, participants selected one of three responses (Go‑left, Go‑right, or NoGo) to a given cue (win or avoid), and learned the probabilistic “cue‑response‑outcome” mappings through feedback (win/neutral/loss). The probability of each response (𝑎) was determined from the corresponding action weights (𝑤) using a softmax function (Equation 3). Action values were updated according to prediction error (Equation 4).


$$\begin{array}{c} p\left(a_{t}|s_{t}\right)=\;\left[\frac{\text{exp}(w(a_{t},s_{t}))}{\sum_{a^{\prime }}\text{exp}(w(a^{\prime },s_{t}))}\right] \end{array}$$ (3)

$$\begin{array}{c} Q_{t}\left(a_{t},s_{t}\right)=\;Q_{t-\text{1}}\left(a_{t},s_{t}\right)+\;\epsilon \left(\rho r_{t}-\;Q_{t-\text{1}}\left(a_{t},s_{t}\right)\right) \end{array}$$ (4)

where *ε* represents the learning rate, *ρ* denotes feedback sensitivity, and *r_t_* is the trial outcome. Trial outcomes were coded as 𝑟 ∈ {−1,0,1}. Because cue valence could only be inferred after the first reward or punishment outcome, initial *Q*‑values were set to 𝜌 × 0.5 for win cues and 𝜌 × −0.5 for avoid cues (i.e., the initial expected outcome is 0.5 for win cues and −0.5 for avoid cues), while these *Q*‑values influenced behavior only once cue valence was known.

To identify the mechanisms of motivational biases in choice, we fitted a baseline model containing learning rate (*ε*) and feedback sensitivity (*ρ*) (Equations 3–4; Table 1 M1). Then, Go bias parameter (𝑏) was added to the action weights of Go responses to capture individual differences in the general tendency to make Go responses, independent of cue information (Table 1 M2). M3a then additionally incorporated a Pavlovian response bias (𝜋), such that static Pavlovian values (𝑉) contributed to the action weights (Equation 5; Table 1 M3a).


$$\begin{array}{c} w\left(a_{t},s_{t}\right)=\;\left\{ \begin{array}{c} @lq\left(a_{t},s_{t}\right)+\;\pi V(s)+\;b\;\;\;\;\;\;\;\;\;\text{if}\;a=\text{Go}\\ q\left(a_{t},s_{t}\right)\;\;\;\;\;\;\;\;\;\;\;\;\;\;\;\;\;\;\;\;\;\;\;\;\;\text{else}\; \end{array}\right. \end{array}$$ (5)

In Model 3b (Table 1 M3b), we introduced an instrumental learning bias parameter (𝜅) to test whether rewards preferentially strengthen Go responses relative to NoGo responses, and whether punishments are less effective at reducing NoGo responses than Go responses. Under this formulation, 𝜅 increases the learning rate following rewarded Go responses while decreasing the learning rate following punished NoGo responses (Equation 6).


$$\begin{array}{c} \epsilon =\;\left\{ \begin{array}{c} @l\epsilon_{\text{0}}+\kappa \;\;\text{if }r_{t}=\;\text{1},\;a=\text{Go}\\ \epsilon_{\text{0}}-\kappa \;\;\text{if }r_{t}=-\text{1},\;a=\text{NoGo}\\ \epsilon_{\text{0}}\;\;\;\;\;\;\text{else} \end{array}\right. \end{array}$$ (6)

In Model 3c (Table 1 M3c), both Pavlovian response bias (*π*) and instrumental learning bias (𝜅) were included to test whether the observed motivational biases in action arise from the complementary contributions of Pavlovian response tendencies and instrumental learning bias.

We used the winning model (M3c) to compute a trial‑wise Pavlovian‑instrumental conflict index (Equation 7) and tested whether cue‑locked midfrontal theta power tracked this conflict strength on a trial‑by‑trial basis. We operationalized conflict as the difference in *Q*‑values between the Pavlovian‑congruent and incongruent response options. Specifically, conflict was calculated as *Q*_NoGo_ − mean(*Q*_Go_) for win cues and as mean(*Q*_Go_) − *Q*_NoGo_ for avoid cues.


$$\begin{array}{c} \text{Conflict}(t)=\text{2valenced}(s_{t})V(s_{t})\left[Q(a_{\text{3}},s_{t})-\frac{Q(a_{\text{1}},s_{t})+Q(a_{\text{2}},s_{t})}{\text{2}}\right] \end{array}$$ (7)

In Equation 7, *valenced*(*s*_*t*_) indicates whether the cue valence had been learned. It was initialized to 0 and set to 1 after the first reward or punishment outcome. *V*(*s*_*t*_) denotes the cue value and was fixed at +0.5 for “win” cues and −0.5 for “avoid” cues. *Q*(*a,s*) represents the instrumental action values, the expected value of choosing action *a* given cue *s*, where *a*_1_, *a*_2_, and *a*_3_ correspond to Go‑left, Go‑right, and NoGo, respectively. The factor of 2 rescales *V(s_t_)* from ±0.5 to ±1, such that the conflict index corresponds to *Q*_NoGo_ − mean(*Q*_Go_) for win cues and mean(*Q*_Go_) − *Q*_NoGo_ for avoid cues. Larger conflict values reflect a greater mismatch between Pavlovian response tendencies elicited by cue‑value and the optimal instrumental action required in the task, which in turn demands stronger cognitive control to inhibit Pavlovian tendencies and execute goal‑directed behavior.

We further extended the winning behavioral model by adding a gain parameter (*β*), which scales how trial-by-trial theta power modulates the motivational biasing of action, and compared a series of alternative theta-modulated models (M4a–d). Each of these models represented a candidate mechanism through which the midfrontal cortex may modulate motivational biases [70] (Equations 8–11).

In Model 4a, we tested whether midfrontal theta power modulated Pavlovian response tendencies. In this model, theta power scaled the Pavlovian bias (*π*) on incongruent trials. Given the form of the equation, a negative *β* value corresponds to theta‑related adaptive suppression of Pavlovian response tendencies during motivational conflict.


$$\begin{array}{c} w\left({\text{Go}}_{t}^{\prime },s_{t}\right)=\;\left\{ \begin{array}{c} @lQ\left({\text{Go}}_{t}^{\prime },s_{t}\right)+\left(\pi +\;\beta *\theta_{t}\right)*V(s)+\;b\;\;\;\;\text{if}\;\text{conflict}\\ Q\left({\text{Go}}_{t}^{\prime },s_{t}\right)+\;\pi V(s)+\;b\;\;\;\;\;\;\;\;\;\;\;\;\;\;\;\;\;\;\;\;\;\text{else}\;\;\;\;\;\;\;\; \end{array}\right. \end{array}$$ (8)

In Model 4b, we tested whether midfrontal theta power modulated the contribution of learned instrumental action values to choice. In this model, theta power acted as a multiplicative gain on the model‑derived *Q*‑value term, such that the instrumental contribution was scaled by (1 − *β* × *θ*_*t*_). Under this specification, a negative *β* indicates that higher theta power increases the contribution of instrumental action values to choice.


$$\begin{array}{c} w\left({\text{Go}}_{t}^{\prime },s_{t}\right)=\;\left\{ \begin{array}{c} \;\;\;\left(\text{1}-\;\beta *\theta_{t}\right)*Q\left({\text{Go}}_{t}^{\prime },s_{t}\right)+\pi V(s)+\;b\;\;\;\;\text{if}\;\text{conflict}\\ Q\left({\text{Go}}_{t}^{\prime },s_{t}\right)+\;\pi V(s)+\;b\;\;\;\;\;\;\;\;\;\;\;\;\;\;\;\;\;\;\;\;\;\;\text{else}\;\;\;\;\;\;\;\; \end{array}\right. \end{array}$$

$$\begin{array}{c} w\left(\text{NoG}{\text{o}}_{\text{t}},s_{t}\right)=\;\left\{ \begin{array}{c} \left(\text{1}-\;\beta *\theta_{t}\right)*Q\left(\text{NoG}o_{t},s_{t}\right)\;\;\;\;\;\;\text{if}\;\text{conflict}\\ Q\left(\text{NoG}{\text{o}}_{t},s_{t}\right)\;\;\;\;\;\;\;\;\;\;\;\;\;\;\;\;\;\;\;\;\;\;\;\;\;\text{else}\;\;\;\;\;\;\; \end{array}\right. \end{array}$$ (9)

In Model 4c, we developed an exploratory arbitration model to test whether midfrontal theta power shifted the balance between Pavlovian and instrumental control, rather than selectively modulating either Pavlovian response tendencies or the instrumental contribution. This model was not intended as an extension of a parameter already selected in the M3 behavioral family; instead, it served as an alternative comparison model to evaluate whether theta acted through a more global Pavlovian‑instrumental trade‑off mechanism. Specifically, M4c used a Pavlovian‑instrumental trade‑off parameter *τ*, and *β* captured whether theta power shifted decision weighting toward Pavlovian or instrumental control on motivationally incongruent trials. In this model, a positive *β* indicates a shift toward stronger Pavlovian tendencies and weaker instrumental contribution, whereas a negative *β* indicates a shift toward stronger instrumental contribution and weaker Pavlovian tendencies.


$$\begin{array}{c} w\left({\text{Go}}_{t}^{\prime },s_{t}\right)=\;\left\{ \begin{array}{c} @l\left(\text{1}-(\tau +\;\beta *\theta_{t}\right))*Q\left({\text{Go}}_{t}^{\prime },s_{t}\right)+\left(\tau +\;\beta *\theta_{t}\right)*V(s)+\;b\;\;\text{if}\;\text{conflict}\\ (\text{1}-\tau )*Q\left(\text{G}{\text{o}}_{t}^{\prime },s_{t}\right)+\;\tau *V(s)+\;b\;\;\;\;\;\;\;\;\;\;\;\;\;\;\;\;\;\;\;\;\;\;\;\;\;\;\;\;\;\text{else}\;\;\;\;\;\;\; \end{array}\right. \end{array}$$

$$\begin{array}{c} w\left(\text{NoG}{\text{o}}_{t},s_{t}\right)=\;\left\{ \begin{array}{c} @l\left(\text{1}-(\tau +\;\beta *\theta_{t}\right))*Q\left(\text{NoG}{\text{o}}_{t},s_{t}\right)\;\;\;\;\text{if}\;\text{conflict}\\ (\text{1}-\tau )*Q\left(\text{NoG}o_{t},s_{t}\right)\;\;\;\;\;\;\;\;\;\;\;\;\;\;\;\;\;\text{else}\;\;\;\;\;\;\;\;\; \end{array}\right. \end{array}$$ (10)

In Model 4d, we tested whether midfrontal theta power modulated instrumental learning bias. In this model, *β* scaled the biased learning‑rate terms for rewarded Go responses and punished NoGo responses, allowing them to move toward the unbiased learning rate *ε*_0_. Thus, a larger positive *β* indicates that theta power reduces the instrumental learning bias and makes updating more similar to the unbiased learning rate.


$$\begin{array}{c} \varepsilon_{\text{rewarded}\;\text{Go}}=\;\left\{ \begin{array}{c} @l\left(\text{1}-\beta *\theta_{t}\right)*\left(\varepsilon_{\text{0}}+\kappa \right)+\left(\beta *\theta_{t}\right)*\epsilon_{\text{0}}\;\;\;\text{if}\;\text{conflict}\;\;\;\\ \epsilon_{\text{0}}+\kappa \;\;\;\;\;\;\;\;\;\;\;\;\;\;\;\;\;\;\;\;\;\;\;\;\;\;\;\;\;\;\;\;\;\;\;\;\;\;\;\;\;\;\text{else}\;\;\;\;\;\;\;\;\; \end{array}\right. \end{array}$$

$$\begin{array}{c} \varepsilon_{\text{punished}\;\text{NoGo}}=\;\left\{ \begin{array}{c} @l\left(\text{1}-\beta *\theta_{t}\right)*(\epsilon_{\text{0}}-\kappa )+(\beta *\theta_{t})*\epsilon_{\text{0}}\;\;\;\text{if}\;\text{conflict}\;\;\;\\ \epsilon_{\text{0}}-\kappa \;\;\;\;\;\;\;\;\;\;\;\;\;\;\;\;\;\;\;\;\;\;\;\;\;\;\;\;\;\;\;\;\;\;\;\;\;\;\;\;\;\;\;\;\;\text{else}\;\;\;\;\;\;\;\;\; \end{array}\right. \end{array}$$ (11)

Existing theories suggest that an important function of the midfrontal cortex is to detect motivational conflict and to “alert” task‑relevant regions to implement control by synchronizing their activity with that of the midfrontal cortex [54]. This view implies that the degree of synchronization with task‑relevant regions, rather than local power alone, may be a better predictor of an individual’s ability to reduce motivational biases. Finally, to assess whether theta‑related modulation of motivational biases depended on long‑range network coordination, we extended the same modeling framework to an intersite phase synchrony modulation family (M5a–M5d). In these models, single‑trial theta‑band phase synchrony estimates replaced local midfrontal theta power (*θ*_*t*_) as the modulation signal, while the mathematical architecture, parameter constraints, and sign conventions were inherited from the corresponding M4 power‑based models. Specifically, we tested whether trial‑by‑trial MFC‑lPFC theta phase synchrony (ISPS_lPFC,t_; M5a, Equation 12) and MFC‑motor theta phase synchrony (ISPS_motor‑contra,t_; M5b, Equation 13) modulated the instrumental contribution during choice.


$$\begin{array}{c} w\left({\text{Go}}_{t}^{\prime },s_{t}\right)=\;\left\{ \begin{array}{c} \left(\text{1}-\beta *\text{ISP}{\text{S}}_{\text{lPFC},t}\right)*Q\left({\text{Go}}_{t}^{\prime },s_{t}\right)+\pi V(s)+b\;\;\;\;\;\;\text{if}\;\text{conflict}\\ Q\left({\text{Go}}_{t}^{\prime },s_{t}\right)+\pi V(s)+b\;\;\;\;\;\;\;\;\;\;\;\;\;\;\;\;\;\;\;\;\;\;\;\;\;\;\;\;\;\;\;\;\;\text{ else}\;\;\;\;\;\;\;\; \end{array}\right. \end{array}$$

$$\begin{array}{c} w\left(\text{NoG}{\text{o}}_{t},s_{t}\right)=\;\left\{ \begin{array}{c} @l\left(\text{1}-\beta *\text{ISP}{\text{S}}_{\text{lPFC},t}\right)*Q\left(\text{NoG}{\text{o}}_{t},s_{t}\right)\;\;\;\;\;\;\text{if}\;\text{conflict}\\ Q\left(\text{NoG}{\text{o}}_{t},s_{t}\right)\;\;\;\;\;\;\;\;\;\;\;\;\;\;\;\;\;\;\;\;\;\;\;\;\;\;\;\;\;\;\;\;\;\;\;\text{else}\;\;\;\;\;\;\;\; \end{array}\right. \end{array}$$ (12)

$$\begin{array}{c} w\left({\text{Go}}_{t}^{\prime },s_{t}\right)=\;\left\{ \begin{array}{c} @l\left(\text{1}-\beta *\text{ISP}{\text{S}}_{\text{motor}-\text{contra},t}\right)*Q\left({\text{Go}}_{t}^{\prime },s_{t}\right)+\pi V(s)+b\;\;\;\text{if}\;\text{conflict}\\ Q\left({\text{Go}}_{t}^{\prime },s_{t}\right)+\pi V(s)+b\;\;\;\;\;\;\;\;\;\;\;\;\;\;\;\;\;\;\;\;\;\;\;\;\;\;\;\;\;\;\;\;\;\;\;\;\;\;\;\text{else}\;\;\;\;\;\;\;\; \end{array}\right. \end{array}$$

$$\begin{array}{c} w\left(\text{NoG}{\text{o}}_{t},s_{t}\right)=\;\left\{ \begin{array}{c} @l\left(\text{1}-\beta *\text{ISP}{\text{S}}_{\text{motor}-\text{contra},t}\right)*Q\left(\text{NoG}{\text{o}}_{t},s_{t}\right)\;\;\;\;\text{if}\;\text{conflict}\\ Q\left(\text{NoG}{\text{o}}_{t},s_{t}\right)\;\;\;\;\;\;\;\;\;\;\;\;\;\;\;\;\;\;\;\;\;\;\;\;\;\;\;\;\;\;\;\;\;\;\;\;\;\;\;\;\text{else}\;\;\;\;\;\;\; \end{array}\right. \end{array}$$ (13)

We further tested whether MFC‑lPFC theta phase synchrony (ISPS_lPFC,t_; M5c, Equation 14) and MFC‑motor theta phase synchrony (ISPS_motor‑contra,t_; M5d, Equation 15) modulated Pavlovian bias.


$$\begin{array}{c} w\left({\text{Go}}_{t}^{\prime },s_{t}\right)=\;\left\{ \begin{array}{c} Q\left({\text{Go}}_{t}^{\prime },s_{t}\right)+\left(\pi +\beta *\text{ISP}{\text{S}}_{\text{lPFC},t}\right)*V(s)+b\;\;\;\text{if}\;\text{conflict}\\ Q\left({\text{Go}}_{t}^{\prime },s_{t}\right)+\pi V(s)+b\;\;\;\;\;\;\;\;\;\;\;\;\;\;\;\;\;\;\;\;\;\;\;\;\;\;\;\;\;\;\text{else}\;\;\;\;\;\;\; \end{array}\right. \end{array}$$ (14)

$$\begin{array}{c} w\left({\text{Go}}_{t}^{\prime },s_{t}\right)=\;\left\{ \begin{array}{c} @lQ\left({\text{Go}}_{t}^{\prime },s_{t}\right)+\left(\pi +\beta *\text{ISP}{\text{S}}_{\text{motor}-\text{contra},t}\right)*V(s)+b\;\;\text{if}\;\text{conflict}\\ Q\left({\text{Go}}_{t}^{\prime },s_{t}\right)+\pi V(s)+b\;\;\;\;\;\;\;\;\;\;\;\;\;\;\;\;\;\;\;\;\;\;\;\;\;\;\;\;\;\;\;\;\;\;\;\;\;\;\;\text{else}\;\;\;\;\;\;\;\; \end{array}\right. \end{array}$$ (15)

We used hierarchical Bayesian sampling to estimate parameters at both the group and subject levels. The group‑level parameters 𝑋 served as priors for the individual‑level parameters 𝑥, such that 𝑥∼𝒩(𝑋,𝜎). Hyperpriors on 𝜎 followed a half‑Cauchy distribution with scale 2. The group‑level means 𝑋 were given weakly informative, zero‑centered priors: 𝑋_ε,𝜅_∼𝒩(0,2) and 𝑋_𝜌,𝑏,𝜋,𝛽_∼𝒩(0,3). All parameters were defined on the real line, except for *ρ*, which was constrained to be positive via an exponential transform, and *ε*, which was constrained to [0,1] via an inverse‑logit transform. To ensure that the effect of 𝜅 on ε was symmetric in model space (i.e., after inverse‑logit transformation and restriction to [0,1]), ε was computed as specified in Equation 16.


$$\begin{array}{c} \epsilon =\;\left\{ \begin{array}{c} @l\epsilon_{\text{0}}=\text{inv}.\text{logit}(\epsilon )\;\;\;\;\;\;\;\;\;\;\;\;\;\;\;\;\;\;\;\;\;\;\;\;\;\;\;\;\;\;\;\;\;\;\;\;\;\;\;\;\;\;\;\;\;\;\;\;\;\;\;\;\;\;\;\;\;\;\;\;\;\;\;\;\;\;\;\;\;\;\;\;\;\;\\ \epsilon_{\text{punished}\;\text{NoGo}}=\text{inv}.\text{logit}(\epsilon -\kappa )\;\;\;\;\;\;\;\;\;\;\;\text{if}\;\epsilon_{\text{0}}<\text{0}.\text{5}\;\;\;\;\;\;\;\;\;\;\;\;\;\;\;\;\;\;\;\;\;\;\;\\ \epsilon_{\text{rewarded}\;\text{Go}}=\;\epsilon_{\text{0}}+\left(\epsilon_{\text{0}}-\epsilon_{\text{punished}\;\text{NoGo}}\right)\;\;\;\text{if}\;\epsilon_{\text{0}}<\text{0}.\text{5}\;\;\;\;\;\;\;\;\;\;\;\;\;\;\;\;\;\;\;\;\;\;\;\;\;\; \end{array}\right. \end{array}$$

$$\begin{array}{c} \epsilon =\;\left\{ \begin{array}{c} \epsilon_{\text{rewarded}\;\text{Go}}=\;\text{inv}.\text{logit}(\epsilon +\kappa )\;\;\;\;\;\;\;\;\;\;\;\;\;\;\;\;\;\;\text{if}\;\epsilon_{\text{0}}>\text{0}.\text{5}\;\;\;\;\;\;\;\;\;\;\;\\ \epsilon_{\text{punished}\;\text{NoGo}}=\epsilon_{\text{0}}+\left(\epsilon_{\text{0}}-\epsilon_{\text{rewarded}\;\text{Go}}\right)\;\;\;\;\;\;\;\text{if}\;\epsilon_{\text{0}}>\text{0}.\text{5}\;\;\;\;\;\;\;\;\;\;\; \end{array}\right. \end{array}$$ (16)

Model estimation was performed in R using RStan (version 2.32.6; Stan version 2.32.2) with Markov chain Monte Carlo (MCMC) sampling. Four chains were run for each model, with 200 warm‑up iterations followed by 1,000 sampling iterations per chain, yielding 4,000 posterior samples in total. Convergence was considered satisfactory when R‑hat < 1.1 for all parameters, which was achieved for all fitted models. Model comparison was based on the WAIC, with lower WAIC values indicating better expected out-of-sample predictive performance after accounting for model complexity. Differences in WAIC of 2–6, 6–10, and >10 were interpreted as positive, strong, and very strong evidence, respectively, in favor of the model with the lower WAIC. For completeness, we also report the proportion of explained variance (*R*^2^) for each model. However, WAIC was used for formal model selection because it explicitly penalizes additional model complexity and accommodates differences in the amount of behavioral variance that individual parameters can potentially explain.

### Individual regulatory orientation

To characterize individual differences in theta‑synchrony-dependent regulatory orientation under motivational conflict, we extracted participant‑level posterior estimates of two model‑derived modulation parameters. The first parameter (𝛽_*π*_) quantified the modulation of the Pavlovian bias and was obtained from M5d. The second parameter (𝛽_*Q*_) quantified the modulation of the instrumental action‑value contribution and was obtained from M5a for healthy control participants and M5b for people with OCD. For each participant, the posterior mean of the corresponding individual‑level parameter was used in subsequent analyses. We then represented these two complementary modulation components within a two‑dimensional regulatory strategy space, such that each participant was characterized by a vector jointly determined by the Pavlovian‑bias and instrumental action‑value contribution modulation parameters. This two‑dimensional representation provided the basis for quantifying both the relative orientation and the overall magnitude of each participant’s regulatory profile.

To convert this two‑dimensional regulatory profile into a single continuous index suitable for symptom‑correlation analyses, we defined a regulatory orientation angle, Φ_reg_. For interpretability, we re‑expressed the modulation estimates as control indices by setting PCI = −𝛽_*π*_ and QCI = −𝛽_*Q*_, such that higher PCI values reflected stronger suppression of Pavlovian response tendencies whereas higher QCI values reflected greater contribution of instrumental action values. PCI and QCI were then separately *z*‑standardized within each group to obtain PCI_z_ and QCI_z_. We subsequently quantified regulatory orientation as the angular direction of each participant’s vector in the standardized two-dimensional control space defined by PCIz and QCIz, using the four‑quadrant inverse tangent function (Equation 17) [152].


$$\begin{array}{c} \Phi_{reg}=\text{atan2}\left(\text{QC}{\text{I}}_{z},\text{PC}{\text{I}}_{z}\right)\times \frac{\text{1}\text{8}\text{0}}{\pi } \end{array}$$ (17)

The rationale for this index is that angular measures can effectively capture trade‑offs between two competing regulatory targets. Shifts in Φ_reg_ toward different regions of the two‑dimensional control space reflect individual differences in the relative weighting of regulatory modulation across Pavlovian response tendencies and instrumental action‑value contribution. Thus, Φ_reg_ provides a continuous measure of regulatory orientation, thereby enabling the potential clinical relevance of these individual differences to be examined in relation to OCD symptom severity.

Finally, we examined the association between Φ_reg_ and OCD symptom severity, indexed by the Y-BOCS total score and its subscales (Obsessions and Compulsions). Because the observed Φ_reg_ values were well separated from the circular discontinuity at ±180° and did not exhibit wrap‑around across this boundary, we treated Φ_reg_ as an approximately continuous orientation measure for inferential analyses. Symptom associations were assessed using a residual‑based partial Spearman approach, in which both Φ_reg_ and the symptom score of interest were residualized with respect to covariates before their residuals were correlated nonparametrically. Analyses controlled for Beck Depression Inventory (BDI) and Beck Anxiety Inventory (BAI) scores, with Barratt Impulsiveness Scale (BIS-11) scores additionally included in a more stringent model. To assess whether the associations depended on the arbitrary angular zero point, we further performed a rotation‑robustness analysis by uniformly shifting Φ_reg_ over a range that did not introduce wrap-around (0°–30°) and repeating the partial Spearman analyses.

## Supporting information

S1 File Supplementary analyses and results, together with the corresponding figures, are included in this file to support the robustness and interpretation of the main findings. (DOCX)

S1 Table Full results of the logistic mixed-effects models examining the association between pre-response theta and trial-level response accuracy. (XLSX)

S1 Data Underlying numerical data for graphs in Fig 2A–2F. (XLSX)

S2 Data Underlying numerical data for graphs in Fig 3A–3E. (XLSX)

S3 Data Underlying numerical data for graphs in Fig 4C–4F. (XLSX)

S4 Data Underlying numerical data for graphs in Fig 5A–5D. (XLSX)

S5 Data Underlying numerical data for graphs in Fig 6A and 6B. (XLSX)

S6 Data Underlying numerical data for panel B of Fig B in Text B, panel B of Fig C in Text C, and Figs F–G in Text G of S1 File. (XLSX)

## Abbreviations

ANOVA: analysis of variance
BAI: Beck Anxiety Inventory
BDI: Beck Depression Inventory
EEG: electroencephalography
ERP: event‑related potential
GLMM: generalized linear mixed model
HC: healthy controls
HDI: highest‑density interval
ICA: independent component analysis
ISPS: intersite phase synchrony
lPFC: lateral prefrontal cortex
MCMC: Markov chain Monte Carlo
MFC: midfrontal cortex
OCD: obsessive‑compulsive disorder
PCA: principal component analysis
RT: reaction time
SEM: standard error of the mean
WAIC: Watanabe‑Akaike Information Criterion
