# Supplementary material for: Impaired midfrontal‑motor theta phase synchronization characterizes maladaptive motivational behavior in people with obsessive‑compulsive disorder
Source: PLoS Biol. 2026 Sep 1;24(9):e3003979. doi: 10.1371/journal.pbio.3003979 (PMC13568527; doi:10.1371/journal.pbio.3003979)
Supplement: Supplementary file 1 [file pbio.3003979.s001.docx]

**Supporting Information for**

Impaired midfrontal‑motor theta phase synchronization characterizes maladaptive motivational behavior in people with obsessive‑compulsive disorder

Yu Pang, Dongsheng Zhou, Ziwen Peng, Wanting Liu, Ruojie Huang, Carol A Seger, Qi Chen

Corresponding author:

E-mail: chenqi@szu.edu.cn (Q.C)

**Supporting Information**

The supplementary materials provide additional analyses and results related to the main findings.

- **Text A. Behavioral results of the forced-choice transfer phase.** This section described analyses of valence learning and action preference in the transfer task, including whether the observed Go bias could be distinguished from differences in cumulative outcomes.
- **Text B. ERP analysis of cue‑locked midfrontal N2.** This section presented a supplementary ERP analysis of cue‑locked midfrontal N2 activity to examine conflict‑related N2 modulation within each group and between‑group differences during cue processing.
- **Text C. Pre‑response midfrontal theta analysis.** This section examined conflict‑related pre‑response midfrontal theta during correct Go trials and used trial‑level linear mixed‑effects models to test whether the group difference remained after controlling for trial‑level reaction time (RT), subject‑level RT variability, and trial order.
- **Text D. Single‑trial midfrontal theta power.** This section examined whether single‑trial midfrontal theta estimates reproduced the conflict‑related pattern observed in the trial‑averaged analyses and assessed whether this effect could be explained by error‑related activity using mixed‑effects models and complementary correct‑ and error‑trial analyses.
- **Text E. Feedback‑locked midfrontal theta power.** This section examined how feedback‑locked midfrontal theta power was modulated by feedback outcome, cue valence, and response, and used repeated‑measures analyses to test whether theta activity reflected instrumental learning bias and differed between healthy controls (HC) and people with obsessive-compulsive disorder (OCD).
- **Text F. Single‑trial midfrontal**‑**lateral prefrontal and midfrontal**‑**motor theta phase synchrony.** This section examined whether conflict‑related midfrontal‑lateral prefrontal and midfrontal‑motor theta phase synchrony observed at the trial‑averaged level was reproduced in single‑trial analyses, and further tested whether midfrontal‑motor synchrony depended on the functional state of the motor region before these neural measures were entered into the computational models.
- **Text G. Evaluation of midfrontal theta phase‑synchrony modulation of instrumental contribution and Pavlovian bias within a single model.** This section jointly estimated phase‑synchrony modulation of instrumental contribution and Pavlovian bias within the same model likelihood to test whether the original subject‑level β estimates, regulatory‑orientation patterns, and their clinical associations remained robust when both synchrony effects were modeled simultaneously.

**Text A. Behavioral results of the forced-choice transfer phase**

Both groups selected win cues as more rewarding than avoid cues (HC: *t*(36) = 33.47, *p* < 0.001, Cohen’s *d_z_*= 5.50; OCD: *t*(35) = 24.65, *p* < 0.001, Cohen’s *d_z_*= 4.11), confirming robust learning of cue valence. In addition, participants showed a systematic preference for Go cues over NoGo cues (HC: *t*(36) = 2.94, *p* = 0.006, Cohen’s *d_z_*= 0.48; OCD: *t*(35) = 2.57, *p* = 0.015, Cohen’s *d_z_*= 0.43). Importantly, this Go preference was not attributable to objectively better outcomes for Go cues, as the cumulative outcomes did not differ significantly between Go and NoGo cues in either group (HC: *t*(36) = 1.09, *p* = 0.29, Cohen’s *d_z_*= 0.18; OCD: *t*(35) = 1.41, *p* = 0.17, Cohen’s *d_z_*= 0.24). Together, these results suggest an inflation of subjective value for Go relative to NoGo cues that cannot be explained by differential reinforcement history.

**Text B. ERP analysis of cue-locked midfrontal N2**

To supplement the time‑frequency findings reported in the main text, we conducted an exploratory analysis of cue‑locked event‑related potential (ERP) activity using the same preprocessed electroencephalography (EEG) dataset. Based on the existing ERP literature on conflict monitoring, we focused on the N2 component over the midfrontal region. Previous studies have shown that the frontocentral or midfrontal N2 is associated with conflict detection, interference control, and response competition, with conflict or incongruent conditions typically eliciting larger and more negative N2 amplitudes [1–5]. Therefore, we defined a midfrontal region of interest (ROI) comprising electrodes Fz, Cz, and FCz. This electrode selection is consistent with recent ERP studies examining N2‑related conflict monitoring using frontocentral or midfrontal ROIs [1,3,4]. In the present task, incongruent trials included the NoGo‑to‑Win and Go‑to‑Avoid conditions, in which Pavlovian response tendencies conflicted with the required instrumental response. Congruent trials included the Go‑to‑Win and NoGo‑to‑Avoid conditions. A time‑resolved comparison between incongruent and congruent trials identified a cue‑locked N2 time window from 288ms to 350ms, during which the difference between the two conditions was significant. Mean N2 amplitudes were therefore extracted from the 288–350ms time window over the midfrontal ROI. These extracted amplitudes were then entered into subsequent statistical analyses. Specifically, paired‑samples *t*‑tests were conducted separately within the HC and OCD groups to compare N2 amplitudes between incongruent and congruent trials. In addition, an independent‑samples *t*‑test was conducted to compare N2 amplitudes between the HC and OCD groups under the incongruent condition.

As shown in Fig A, incongruent trials elicited significantly more negative N2 amplitudes than congruent trials (*t*(36) = −5.55, *p* < 0.001, Cohen’s *d*_z_ = −0.91) in the HC group. The result was consistent with previous ERP studies showing that conflict, interference, or competing response tendencies typically elicit enhanced N2 activity, reflecting early conflict detection and interference monitoring [2,4,5].

**Fig A. N2 congruency effects in the HC and OCD groups.** N2 amplitudes were extracted from a 288–350ms time window over the midfrontal region of interest, corresponding to Fz/Cz/FCz. The left panels show grand‑average ERP waveforms for congruent and incongruent trials in the HC group (A) and OCD group (B). Shaded areas indicate the N2 analysis window, and shaded error bands represent the standard error of the mean (SEM). The right panels show scalp topographies of the N2 congruency effect, calculated as incongruent minus congruent amplitudes during the same time window. Color values indicate amplitude differences between conditions in μV.

A similar congruency effect was observed in the OCD group. Incongruent trials elicited significantly more negative N2 amplitudes than congruent trials (*t*(35) = −5.16, *p* < 0.001, Cohen’s *d_z_* = −0.86) (Fig A). This result suggested that people with OCD showed reliable ERP sensitivity to motivational conflict, as indexed by enhanced N2 amplitudes for incongruent relative to congruent trials. Although previous findings on N2 abnormalities in clinical populations have been mixed, recent ERP studies indicate that people with OCD may still show detectable conflict‑related N2 modulation under interference or conflict conditions [6–8].

We further compared N2 amplitudes between the HC and OCD groups during incongruent trials. As shown in Fig B, the comparison between groups did not reach significance (*t*(71) = −1.00, *p* = 0.318, Cohen’s *d* = −0.23), indicating that incongruent‑trial N2 amplitude did not reliably differ between groups. This finding is in line with the cue‑locked midfrontal theta power results reported in the main text, where both groups showed conflict‑sensitive midfrontal neural responses during cue processing. Taken together, these supplementary ERP findings suggest that basic cue‑level conflict sensitivity was evident in both groups. Given that the central aim of the present study was to examine dynamic time‑frequency mechanisms of motivational conflict processing, these N2 results are reported as supplementary analyses and do not alter the primary focus on cue‑locked midfrontal theta activity and downstream phase synchrony.

**Fig B. N2 responses to incongruent trials in HC and OCD groups.** **(A)** Grand‑average ERP waveforms in incongruent trials. ERP waveforms were averaged over the midfrontal ROI electrodes (Fz/FCz/Cz). Solid lines indicate group‑averaged waveforms, and shaded areas indicate ±1 SEM. The gray shaded window marks the N2 time window from 288 to 350ms. **(B)** Group comparison of N2 amplitude. Mean N2 amplitudes were extracted from the 288–350ms time window over the same midfrontal ROI. Each dot represents one participant, white circles indicate group means, and error bars indicate ±1 SEM. HC is shown in orange and OCD in blue. The *p* value was obtained from an independent‑samples *t*-test comparing the HC and OCD groups. The numerical data underlying panel B can be found in S6 Data.

**Text C. Pre-response midfrontal theta analysis**

In healthy controls, theta power increased before the correct response, whereas in people with OCD, theta enhancement was more temporally overlapped with the response. However, overlap between theta activity and the response does not by itself establish impaired control efficacy. It may also reflect motor-related activity, RT differences, or RT variability. We therefore conducted additional analyses to clarify whether the pre‑response theta effect remained after accounting for RT‑related factors.

For subsequent trial‑level analyses, midfrontal theta power was extracted from the response‑preceding windows used in the response‑locked analyses. In HC participants, theta power was extracted from −550ms to −126ms before the response. In the OCD group, no clearly significant response‑preceding theta cluster was identified. Therefore, for the OCD group, theta power was extracted from an exploratory, descriptively defined interval from −676ms to −250ms before the response, corresponding to the period in which theta power was descriptively higher for Go‑to‑Avoid than for Go‑to‑Win trials. For each trial, theta power was averaged across the midfrontal channels and the 4–8Hz frequency range.

To examine whether the pre‑response theta effect could be explained by differences in reaction time, response‑time variability, or trial progression, we restricted the analysis to correct Go trials only. Single‑trial pre‑response midfrontal theta power was entered as the dependent variable in three linear mixed‑effects models (LMMs). In all models, Group indicated diagnostic group, HC versus OCD, and Conflict indicated motivational congruency among Go trials, with Go‑to‑Win as the congruent condition and Go‑to‑Avoid as the incongruent conflict condition. A random intercept was included for participant to account for repeated observations within subjects.

First, LMM1 controlled for trial‑level RT:

$\text{Theta}_{ij}=\beta_{0}+\beta_{1}\text{Group}_{i}+\beta_{2}\text{Conflict}_{ij}+\beta_{3}\text{Group}_{i}\times\text{Conflict}_{ij}+\beta_{4}\text{RT}_{(z,ij)}+u_{i}+\varepsilon_{ij}$ (1)

Second, LMM2 additionally controlled for subject‑level RT variability:

$\begin{matrix} \text{Theta}_{ij}=\beta_{0}+\beta_{1}\text{Group}_{i}+\beta_{2}\text{Conflict}_{ij}+\beta_{3}\text{Group}_{i}\times\text{Conflict}_{ij}+\beta_{4}\text{RT}_{(z,ij)}+ \\ \text{ }\beta_{5}\text{RT Variability}_{(z,i)}+u_{i}+\varepsilon_{ij} \end{matrix}$ (2)

Third, LMM3 further controlled for trial order:

$\begin{matrix} \text{Theta}_{ij}=\beta_{0}+\beta_{1}\text{Group}_{i}+\beta_{2}\text{Conflict}_{ij}+\beta_{3}\text{Group}_{i}\times\text{Conflict}_{ij}+\beta_{4}\text{RT}_{(z,ij)}+ \\ \text{ }\beta_{5}\text{RT} \text{Variability}_{(z,i)}+\beta_{6}\text{Trial}_{(z,ij)}+u_{i}+\varepsilon_{ij} \end{matrix}$ (3)

Here, Theta_ij_ denotes pre-response midfrontal theta power on trial j for participant i. Groupi indicates diagnostic group, HC versus OCD. Conflict_ij_ indicates motivational congruency on trial j, with Go‑to‑Win as the congruent condition and Go‑to‑Avoid as the incongruent conflict condition. RT_(z,ij)_ denotes the within‑subject standardized reaction time for each trial. RT variability_(z,i)_ denotes standardized subject-level RT variability, computed as the standard deviation of RT across trials for each participant. Trial_(z,ij)_ denotes the within‑subject standardized trial number. The random intercept u_i_ captured participant‑specific baseline differences in pre‑response theta power, and the residual term ε_ij_ represented trial‑level unexplained variance. The critical term in all three models was the Group × Conflict interaction, which tested whether the Go‑to‑Avoid versus Go‑to‑Win difference in pre‑response theta power differed between HC and people with OCD after accounting for RT‑related factors and trial‑order‑related effects.

As shown in Fig C, the main effect of Group was significant in all three models, indicating an overall group difference in pre-response theta power (LMM1, β = −0.42, *SE* = 0.08, 95% CI [−0.58, −0.25], *t*(71.81) = −5.03, *p* < 0.001; LMM2, β = −0.39, *SE* = 0.09, 95% CI [−0.56, −0.22], *t*(71.81)= −4.59, *p* < 0.001; LMM3, β = −0.39, *SE* = 0.09, 95% CI [−0.56, −0.22], *t*(71.63)= −4.59, *p* < 0.001). The main effect of Conflict was significant in LMM1 (β = 0.06, *SE* = 0.02, 95% CI [0.01, 0.10], *t*(10160.44)= 2.53, *p* = 0.011), and LMM2 (β = 0.06, *SE* = 0.02, 95% CI [0.01, 0.10], *t*(10159.52)= 2.52, *p* = 0.012), but was no longer significant after trial order was included in LMM3 (β = 0.03, *SE* = 0.02, 95% CI [−0.01, 0.08], *t*(10159.69) = 1.42, *p* = 0.154). This suggested that the overall Go‑to‑Avoid versus Go‑to‑Win theta difference was partly accounted for by trial‑order‑related variance.

**Fig C. Pre‑response midfrontal theta activity during motivational conflict after controlling for RT‑related and trial‑order effects.** **(A)** Fixed‑effect estimates of predictors of pre‑response theta. Trial‑level linear mixed‑effects models were used to examine whether conflict‑related pre‑response theta modulation was associated with group differences after accounting for RT‑related factors and trial‑order effects. LMM1 controlled for trial‑level RT; LMM2 additionally controlled for subject‑level RT variability; LMM3 further controlled for trial order. Bars indicate fixed‑effect beta estimates, and error bars indicate 95% confidence intervals. **(B)** Group‑specific Go‑to‑Win versus Go‑to‑Avoid differences. The linear mixed‑effects models were fitted at the single‑trial level, whereas the figure displays participant‑level model‑adjusted estimates for visualization. Pre‑response theta estimates are shown separately for Go‑to‑Win and Go‑to‑Avoid trials within each group and model. Each dot represents one participant’s model‑adjusted mean estimate for a given condition. White circles indicate group‑level estimated marginal means, and vertical error bars indicate 95% confidence intervals. Horizontal brackets indicate within‑group Go‑to‑Win versus Go‑to‑Avoid contrasts derived from the corresponding trial‑level mixed‑effects model. ^*^ indicates *p* < 0.05, ^**^ indicates *p* < 0.01, and ^***^ indicates *p* < 0.001; n.s., not significant. The numerical data underlying panel B can be found in S6 Data.

Trial-level RT was positively associated with pre-response theta power in all models (LMM1, β = 0.14, *SE* = 0.02, 95% CI [0.10, 0.19], *t*(10137.94) = 6.34, *p* < 0.001; LMM2, β = 0.14, *SE* = 0.02, 95% CI [0.10, 0.19], *t*(10138.17) = 6.34, *p* < 0.001; LMM3, β = 0.21, *SE* = 0.02, 95% CI [0.17, 0.26], *t*(10138.51) = 8.92, *p* < 0.001). Subject‑level RT variability was not significantly associated with pre‑response theta power in LMM2 (β = −0.12, *SE* = 0.09, 95% CI [−0.29, 0.05], *t*(72.75) = −1.44, *p* = 0.155), or LMM3 (β = −0.12, *SE* = 0.09, 95% CI [−0.29, 0.05], *t*(72.75) = −1.45, *p* = 0.152). In contrast, trial order was significantly associated with pre‑response theta power in LMM3 (β = 0.20, *SE* = 0.02, 95% CI [0.16, 0.25], *t*(10137.43) = 8.59, *p* < 0.001), indicating systematic trial‑order‑related changes in theta power.

Notably, the interaction between Group and Conflict remained significant across all three models. In LMM1, the interaction was significant after controlling for trial‑level RT (β = −0.05, *SE* = 0.02, 95% CI [−0.09, 0.00], *t*(10159.43) = −2.04, *p* = 0.042). In LMM2, this interaction remained significant after additionally controlling for subject‑level RT variability (β = −0.05, *SE* = 0.02, 95% CI [−0.09, 0.00], *t*(10160.06) = −2.03, *p* = 0.042). In LMM3, the interaction also remained significant after further controlling for trial order (β = −0.05, *SE* = 0.02, 95% CI [−0.09, 0.00], *t*(10159.90) = −2.12, *p* = 0.034). Thus, the group difference in conflict‑related pre‑response theta modulation was not explained by trial‑level RT, subject‑level RT variability, or trial‑order‑related effects.

As shown in Fig C, simple‑effect analyses further clarified the Group × Conflict interaction. In the HC group, Go‑to‑Avoid trials elicited significantly stronger pre‑response theta than Go‑to‑Win trials across all three models (LMM1, estimated difference = 0.21, *SE* = 0.06, 95% CI [0.09, 0.33], *t*(10159.53) = 3.36, *p* < 0.001; LMM2, estimated difference = 0.21, *SE* = 0.06, 95% CI [0.09, 0.33], *t*(10158.82) = 3.35, *p* < 0.001; LMM3, estimated difference = 0.16, *SE* = 0.06, 95% CI [0.04, 0.28], *t*(10158.81) = 2.60, *p* = 0.009). In contrast, in the OCD group, the Go‑to‑Avoid versus Go‑to‑Win difference was not significant in any model (LMM1, estimated difference = 0.02, *SE* = 0.07, 95% CI [−0.10, 0.15], *t*(10160.31) = 0.37, *p* = 0.708; LMM2, estimated difference = 0.02, *SE* = 0.07, 95% CI [−0.11, 0.15], *t*(10160.61) = 0.37, *p* = 0.713; LMM3, estimated difference = −0.03, *SE* = 0.07, 95% CI [−0.16, 0.10], *t*(10160.63) = −0.44, *p* = 0.663).

Together, these results indicated that healthy controls showed a robust conflict‑related pre‑response theta enhancement, with stronger theta power for Go‑to‑Avoid than Go‑to‑Win trials. This effect remained significant after controlling for trial‑level RT, subject‑level RT variability, and trial order. In contrast, people with OCD did not show a reliable Go‑to‑Avoid versus Go‑to‑Win pre‑response theta difference in any model. Therefore, the reduced pre‑response theta differentiation in OCD cannot be fully explained by slower responses, greater RT variability, or trial‑order‑related changes, but instead reflects an altered or attenuated recruitment of midfrontal theta during motivational conflict.

**Text D. Single‑trial midfrontal theta power**

The main text showed that motivational conflict modulated midfrontal theta power at the trial‑averaged level. Here, we asked whether the same pattern could also be observed in trial‑by‑trial theta estimates. This step was important because these single‑trial neural measures were later included in the computational models. To address this question, we fitted mixed‑effects models with valence (Win vs. Avoid) and required action (Go vs. NoGo) as within‑subject factors. All models included a full random‑effects structure. Before model fitting, theta power values were mean‑corrected and inverse transformed to improve the distribution of the data.

We first focused on correct trials. This allowed us to examine conflict‑related theta signals under conditions in which performance was successful. In healthy controls, the single‑trial analysis showed a significant valence × action interaction (β = −0.007, 95% CI [−0.011, −0.003], χ²(1) = 11.25, *p* < 0.001), whereas the main effect of action (β = −0.004, 95% CI [−0.009, 0.002], χ²(1) = 1.94, *p* = 0.16), and the main effect of valence (β = 0.002, 95% CI [−0.001, 0.005], χ²(1) = 2.17, *p* = 0.14), were not significant. Follow‑up tests showed that the valence effect was significant in Go trials (β = −0.005, 95% CI [−0.009, −0.0004], χ²(1) = 4.63, *p* = 0.03), and in NoGo trials (β = 0.009, 95% CI [0.004, 0.014], χ²(1) = 11.54, *p* < 0.001), with the stronger effect in NoGo trials. When the data were examined from the action side, theta power was higher for NoGo than for Go responses under Win cues (β = −0.011, 95% CI [−0.018, −0.003], χ²(1) = 7.82, *p* = 0.005). Together, these results show that, in healthy controls, single‑trial midfrontal theta power increased under motivational conflict, especially in NoGo conditions.

The OCD group showed a similar overall pattern. On correct trials, the valence × action interaction was significant (β = −0.006, 95% CI [−0.009, −0.003], χ²(1) = 19.24, *p* < 0.001), while the main effect of action (β = −0.002, 95% CI [−0.005, 0.002], χ²(1) = 0.84, *p* = 0.36), and the main effect of valence (β = 0.002, 95% CI [−0.001, 0.006], χ²(1) = 1.34, *p* = 0.25), were not significant. The simple‑effect analyses showed that the valence effect did not reach significance in Go trials (β = −0.004, 95% CI [−0.008, 0.0002], χ²(1) = 3.42, *p* = 0.06), but was significant in NoGo trials (β = 0.008, 95% CI [0.003, 0.013], χ²(1) = 11.80, *p* < 0.001). In addition, theta power was higher for NoGo than for Go responses under Win cues (β = −0.008, 95% CI [−0.012, −0.003], χ²(1) = 9.96, *p* = 0.002). Thus, the OCD group also showed a reliable conflict‑related modulation of single‑trial midfrontal theta power.

We then carried out additional analyses to examine whether the single‑trial theta estimates could have been affected by error‑related activity. This was important because both conflict detection and error processing have been linked to stronger midfrontal theta activity [9–11]. These two processes are also expected to differ in time, with conflict‑related signals usually peaking before the response and error‑related signals emerging after the response. In healthy controls, the time window of interest (400–650ms) fell before the mean response time (679ms). However, some overlap with post‑response activity could still occur because time‑frequency measures are temporally smoothed. In the OCD group, this issue was even more relevant, because the time window of interest (424–800ms) overlapped with the mean response time (700ms). We therefore examined this question in two follow‑up analyses in each group. First, we tested the main effect of accuracy in a model that included both correct and incorrect trials. Second, we tested whether the valence × action interaction was still present when the analysis was restricted to error trials only.

In healthy controls, theta power was higher on correct than on incorrect trials, as shown by a significant main effect of accuracy (β = 0.004, 95% CI [0.001, 0.007], χ²(1) = 7.27, *p* = 0.007). When the analysis was restricted to error trials, the valence × action interaction was not significant (β = −0.002, 95% CI [−0.007, 0.002], χ²(1) = 0.94, *p* = 0.33). Thus, in the healthy control group, there was no evidence that motivational conflict modulated theta power on error trials. In the OCD group, the main effect of accuracy did not reach significance (β = 0.002, 95% CI [−0.0008, 0.005], χ²(1) = 1.96, *p* = 0.16), indicating that theta power was not reliably different between correct and incorrect trials. However, when the analysis was restricted to error trials, the valence × action interaction was not significant (β = −0.002, 95% CI [−0.005, 0.002], χ²(1) = 0.87, *p* = 0.35). Thus, in the OCD group, the error‑trial analyses also did not show a reliable conflict‑related modulation of theta power.

To further evaluate this issue, we also examined the trial‑averaged data with repeated‑measures analysis of variance (ANOVA). In healthy controls, the valence × action interaction was significant on correct trials (*F*(1,36) = 20.16, *p* < 0.001, *η_p_²* = 0.36), but not on error trials (*F*(1,36) = 0.91, *p* = 0.35, *η_p_²* = 0.02). The same pattern was observed in the OCD group: the interaction was significant on correct trials (*F*(1,35) = 33.72, *p* < 0.001, *η_p_²* = 0.49), but not on error trials (*F*(1,35) = 1.64, *p* = 0.21, *η_p_²* = 0.04). Taken together, these results indicate that the conflict‑related theta effect was mainly expressed on correct trials rather than on error trials.

Overall, the single‑trial theta results reproduced the main conflict‑related pattern observed at the trial‑averaged level. In both the HC and OCD groups, midfrontal theta power increased under motivational conflict, and this effect was most clearly observed when participants responded correctly. By contrast, error‑trial analyses did not reveal a reliable conflict‑related modulation of theta power in either group. This pattern suggests that midfrontal theta was more closely linked to successful conflict resolution than to error‑related processing alone. Based on this reasoning, we retained single‑trial theta estimates from both correct and incorrect trials for the computational modeling, so that the relation between trial‑by‑trial theta activity and behavior could be characterized more fully.

**Text E. Feedback‑locked midfrontal theta power**

Previous studies have shown that feedback‑locked midfrontal theta activity is closely linked to reinforcement learning [12–14]. To test whether midfrontal theta power was also related to the modulation of instrumental learning bias, we further examined feedback‑locked midfrontal theta power. In line with the cue‑locked analysis, we selected the same midfrontal cluster and frequency range (4–8Hz) and restricted the analysis to the period of feedback presentation. To identify the time window for extracting feedback-locked data, we employed a time-based permutation test (500 permutations) using feedback-locked, trial-averaged midfrontal theta power as the dependent variable and outcome (preferred versus non-preferred) as the within-subject factor. Neutral feedback following win cues and punishment following avoid cues were defined as non‑preferred outcomes. Reward following win cues and neutral feedback following avoid cues were defined as preferred outcomes. The time window showing a significant difference between non‑preferred and preferred outcomes was taken as the feedback‑locked theta window of interest. Within this window (HC:150–576ms, *p* = 0.004; OCD:200–450ms, *p* =0.010), trial‑by‑trial theta power was extracted for each feedback condition and then averaged at the subject level for subsequent statistical analysis.

To characterize the sensitivity of feedback‑related theta activity to feedback outcome, cue valence, and response, we first conducted separate 2 × 2 × 2 repeated‑measures ANOVAs in the HC and OCD groups. The within‑subject factors were outcome (non‑preferred vs. preferred), valence (win cues vs. avoid cues), and response (Go vs. NoGo). As shown in Fig D, in the HC group, the main effect of outcome was significant (*F*(1, 36) = 13.39, *p* = 0.001, *η_p_²* = 0.27), indicating that non‑preferred outcomes elicited stronger theta activity than preferred outcomes. The main effect of valence was also significant (*F*(1, 36) = 24.71, *p* < 0.001, *η_p_²* = 0.41), with higher theta activity following avoid cues than following win cues. However, the main effect of response did not reach significance (*F*(1, 36) = 3.06, *p* = 0.089, *η_p_²* = 0.08). None of the interaction effects were significant (outcome × valence: *F*(1, 36) = 0.28, *p* = 0.603, *η_p_²* = 0.01; outcome × response: *F*(1, 36) = 0.01, *p* = 0.926, *η_p_²* = 0.001; valence × response: *F*(1, 36) = 3.16, *p* = 0.084, *η_p_²* = 0.08; outcome × valence × response: *F*(1, 36) = 0.14, *p* = 0.712, *η_p_²* = 0.004). Together, these results indicated that feedback‑locked theta activity in the HC group was modulated by both outcome and cue valence. Compared with preferred outcomes, non‑preferred outcomes elicited stronger theta activity, and feedback following avoid cues elicited stronger theta activity than feedback following win cues. Importantly, the outcome effect did not significantly depend on cue valence. In the OCD group, the main effect of outcome was also significant (*F*(1, 35) = 9.13, *p* = 0.005, *η_p_²* = 0.21), indicating that non‑preferred outcomes elicited stronger theta activity than preferred outcomes. The main effect of valence showed a marginal trend (*F*(1, 35) = 4.04, *p* = 0.052, *η_p_²* = 0.10), while the main effect of response was not significant (*F*(1, 35) = 1.98, *p* = 0.168, *η_p_²* = 0.05). Importantly, the interaction between outcome and valence was significant (*F*(1, 35) = 11.89, *p* = 0.001, *η_p_²* = 0.25), indicating that the outcome effect was modulated by cue valence. The remaining interaction effects (outcome × response: *F*(1, 35) = 0.55, *p* = 0.461, *η_p_²* = 0.02; valence × response: *F*(1, 35) = 0.07, *p* = 0.786, *η_p_²* = 0.002; outcome × valence × response: *F*(1, 35) = 0.06, *p* = 0.814, *η_p_²* = 0.002) were not significant.

**Fig D. Feedback‑locked midfrontal theta power in the HC group (A) and in the OCD group (B).** Left panel: Time‑wise permutation testing revealed a significant feedback‑locked time window (150–576ms in the HC group; 200–450ms in the OCD group), during which midfrontal theta power was increased for non‑preferred relative to preferred outcomes (^**^*p* < 0.01). Non‑preferred outcomes included punishment following avoid cues and neutral feedback following win cues, whereas preferred outcomes included reward following win cues and neutral feedback following avoid cues. Within this time window, theta power was also higher for avoid‑cue than for win‑cue contexts. Right panel: Time‑frequency representation and topographical distribution of the contrast between non‑preferred and preferred outcomes. White discs indicate a priori midfrontal channels that remained significant after post hoc Bonferroni correction (α = 0.017).

To clarify how the outcome effect varied across cue valence contexts and to further explain the source of the significant interaction between outcome and valence observed specifically in the OCD group, we next conducted separate 2 × 2 repeated measures ANOVAs for win cues and avoid cues. In the HC group, the main effect of outcome was significant for win cues (*F*(1, 36) = 9.26, *p* = 0.004, *η_p_²* = 0.21), with higher theta activity for neutral than for win outcomes. For avoid cues, the main effect of outcome was also significant (*F*(1, 36) = 10.94, *p* = 0.002, *η_p_²* = 0.23), with higher theta activity for punishment than for neutral outcomes. These results suggested that the HC group showed enhanced theta activity for non‑preferred outcomes for both win cues and avoid cues. Moreover, for win cues, the main effect of response (*F*(1, 36) = 0.01, *p* = 0.914, *η_p_²* = 0.001) and the interaction between outcome and response (*F*(1, 36) = 0.06, *p* = 0.801, *η_p_²* = 0.002) were not significant. For avoid cues, the interaction between outcome and response was not significant (*F*(1, 36) = 0.12, *p* = 0.730, *η_p_²* = 0.003). However, the main effect of response was significant (*F*(1, 36) = 6.92, *p* = 0.012, *η_p_²* = 0.16), indicating that feedback following Go responses elicited stronger theta activity than feedback following NoGo responses in the avoid cue condition.

In contrast to the HC group, the outcome effect in the OCD group was mainly observed for avoid cues. For win cues, the main effect of outcome was not significant (*F*(1, 35) = 0.00, *p* = 0.996, *η_p_²* = 0.001), indicating that the OCD group did not show a significant theta difference between neutral and reward outcomes. The main effect of response was not significant (*F*(1, 35) = 0.85, *p* = 0.362, *η_p_²* = 0.02), and the interaction between outcome and response was also not significant (*F*(1, 35) = 0.14, *p* = 0.713, *η_p_²* = 0.004). However, for avoid cues, the main effect of outcome was significant (*F*(1, 35) = 23.86, *p* < 0.001, *η_p_²* = 0.41), with punishment eliciting stronger theta activity than neutral outcomes. The main effect of response was not significant (*F*(1, 35) = 2.46, *p* = 0.126, *η_p_²* = 0.07), and the interaction between outcome and response was also not significant (*F*(1, 35) = 0.62, *p* = 0.437, *η_p_²* = 0.02).

To examine whether feedback‑locked theta power elicited by neutral feedback differed as a function of cue context, we conducted separate 2 × 2 repeated‑measures ANOVAs for the HC and OCD groups. The within‑subject factors were cue valence (win vs. avoid) and response (Go vs. NoGo), with theta power for neutral outcomes as the dependent variable. In the HC group, the main effect of cue valence was not significant (*F*(1, 36) = 1.03, *p* = 0.316, *η_p_²* = 0.03), indicating that theta power elicited by neutral outcomes did not differ between win cues and avoid cues. The main effect of response (*F*(1, 36) = 0.91, *p* = 0.345, *η_p_²* = 0.03) and the interaction between cue valence and response (*F*(1, 36) = 1.90, *p* = 0.177, *η_p_²* = 0.05) were also not significant. Consistent with the HC group, the main effect of cue valence was not significant in the OCD group (*F*(1, 35) = 1.24, *p* = 0.272, *η_p_²* = 0.03), indicating that theta power elicited by neutral outcomes did not differ between win cues and avoid cues. Neither the main effect of response (*F*(1, 35) = 0.93, *p* = 0.341, *η_p_²* = 0.03) nor the interaction between cue valence and response (*F*(1, 35) = 0.22, *p* = 0.644, *η_p_²* = 0.006) was significant. Together, these results indicated that feedback‑locked theta power for neutral outcomes did not differ as a function of cue valence or response in either group.

Next, we examined whether feedback‑locked theta activity was associated with instrumental learning bias. We focused on two complementary contrasts. First, we contrasted biased learning versus unbiased learning. Biased learning refers to feedback conditions that align with the behavioral learning bias, namely rewarded Go and punished NoGo. Rewarded Go reflects enhanced learning following reward (ε_0_ + κ), whereas punished NoGo reflects reduced learning following punishment (ε_0_– κ). Unbiased learning comprises rewarded NoGo and punished Go, both associated with a neutral learning rate (ε_0_). These unbiased conditions served as the comparison baseline. This contrast tested whether theta activity was greater for feedback conditions consistent with the instrumental learning bias than for their bias‑inconsistent counterparts.

To examine whether the interaction between outcome and response reflected the contrast between biased and unbiased learning, we performed a 2 × 2 repeated‑measures ANOVA, with outcome (reward vs. punishment) and response (Go vs. NoGo) as within‑subject factors and theta power as the dependent variable. As shown in Fig E, in the HC group, the main effect of outcome was significant (*F*(1, 36) = 28.72, *p* < 0.001, *η_p_²* = 0.44), with punishment eliciting stronger theta activity than reward. However, the interaction between outcome and response, which reflects the contrast between biased and unbiased learning conditions, was not significant (*F*(1, 36) = 2.39, *p* = 0.131, *η_p_²* = 0.06, BF₀₁ ≈ 1.05). In the OCD group, the main effect of outcome was also significant (*F*(1, 35) = 9.40, *p* = 0.004, *η_p_²* = 0.21), with punishment eliciting stronger theta activity than reward. However, the interaction between outcome and response was not significant (*F*(1, 35) = 0.53, *p* = 0.472, *η_p_²* = 0.02, BF₀₁ = 1.65). Thus, neither the HC group nor the OCD group exhibited a significant enhancement of feedback‑locked theta activity for feedback conditions aligned with the instrumental learning bias.

**Fig E. Feedback‑locked midfrontal theta power and instrumental learning‑bias contrasts in HC and OCD groups.** **(A)** Biased learning effects on feedback‑locked midfrontal theta power in the HC group. The left panel shows the contrast between bias‑consistent and bias‑inconsistent feedback conditions. Bias‑consistent conditions included rewarded Go and punished NoGo trials, corresponding to enhanced Go learning after reward and reduced NoGo updating after punishment. Bias‑inconsistent conditions included rewarded NoGo and punished Go trials. Feedback‑locked midfrontal theta power did not significantly differ between bias‑consistent and bias‑inconsistent conditions in the HC group. The right panel shows the contrast between Go‑related and NoGo‑related feedback conditions, collapsed across reward and punishment outcomes, reflecting relatively enhanced versus reduced learning. This response‑related contrast was also not significant in the HC group. **(B)** The same contrasts are shown for the OCD group. Feedback‑locked midfrontal theta power did not significantly distinguish bias‑consistent from bias‑inconsistent feedback conditions. The contrast between Go‑related and NoGo‑related feedback conditions was also not significant. Black rectangles indicate the feedback‑locked theta time‑frequency windows used for statistical analysis, identified from the independent non‑preferred versus preferred outcome contrast. Scalp maps show the topographical distribution of each contrast averaged within the corresponding theta time‑frequency window. Color values indicate condition differences in power in dB. FB denotes feedback onset.

The second learning‑related effect was relatively enhanced versus reduced learning. This analysis examined whether feedback‑locked theta activity differed between feedback following Go responses and feedback following NoGo responses. Specifically, relatively enhanced learning corresponded to Go feedback (rewarded Go and punished Go), whereas relatively reduced learning corresponded to NoGo feedback (rewarded NoGo and punished NoGo). In the same 2 × 2 model (outcome × response), this contrast was captured by the main effect of response. The main effect of response was not significant in either the HC group (*F*(1, 36) = 2.14, *p* = 0.152, *η_p_²* = 0.06, BF₀₁ = 2.80) or the OCD group (*F*(1, 35) = 1.61, *p* = 0.213, *η_p_²* = 0.04, BF₀₁ ≈ 3.29), indicating that feedback‑locked theta activity likewise did not distinguish relatively enhanced from relatively reduced learning conditions.

Overall, feedback‑locked theta activity distinguished punishment from reward feedback in both groups, with stronger theta activity for punishment. However, neither group showed a significant interaction between outcome and response nor a significant main effect of response. This suggests that feedback‑locked theta activity was not significantly modulated by instrumental learning biases.

In the group‑level analyses, we first tested whether the two groups differed in the contrast between non‑preferred and preferred outcomes. A 2 (group: HC vs. OCD) × 2 (outcome: non‑preferred vs. preferred) mixed repeated‑measures ANOVA was conducted. The analysis revealed a significant main effect of outcome (*F*(1, 71) = 22.36, *p* < 0.001, *η_p_²* = 0.24), indicating that non‑preferred outcomes elicited stronger theta activity than preferred outcomes. The main effect of group was not significant (*F*(1, 71) = 0.06, *p* = 0.805, *η_p_²* = 0.001), and the interaction between group and outcome was also not significant (*F*(1, 71) = 1.00, *p* = 0.321, *η_p_²* = 0.01). An independent samples t‑test on the non‑preferred minus preferred difference score also showed no significant group difference (*t*(71) = 1.00, *p* = 0.321, Cohen’s *d* = 0.23). We further tested whether the two groups differed in theta activity across four outcomes: punishment, neutral following win cues, neutral following avoid cues, and reward. To examine group differences in response to these feedback types, we conducted a 2 × 4 mixed repeated‑measures ANOVA with group (HC vs. OCD) as the between‑subjects factor and outcome (punishment, neutral_win_, neutral_avoid_, reward) as the within‑subjects factor. A significant main effect of outcome emerged after Greenhouse–Geisser correction (*F*(2.55, 181.03) = 18.63, *p* < 0.001, *η_p_²* = 0.21), indicating that theta activity varied across the four outcomes. The main effect of group was not significant (*F*(1, 71) = 0.06, *p* = 0.805, *η_p_²* = 0.001), and the interaction between group and outcome also did not reach significance (*F*(2.55, 181.03) = 2.25, *p* = 0.095, *η_p_²* = 0.03). These results suggested that the two groups did not differ significantly in their theta power patterns across outcomes. Nevertheless, to explore potential trends within each group, we conducted Bonferroni‑corrected post hoc comparisons separately for the HC and OCD groups. Bonferroni‑corrected post hoc comparisons within each group showed that, in the HC group, punishment was significantly higher than neutral_win_ (*p* = 0.006), neutral_avoid_ (*p* = 0.013), and reward (*p* < 0.001). Neutral_win_ (*p* = 0.026) and neutral_avoid_ (*p* = 0.027) were also significantly higher than reward. In the OCD group, punishment was significantly higher than neutral_win_ (*p* = 0.003), neutral_avoid_ (*p* < 0.001), and reward (*p* = 0.025). However, neutral_win_, neutral_avoid_, and reward did not differ from each other (all *p*s = 0.999). However, these within‑group patterns should be interpreted with caution given the absence of a significant interaction between group and outcome.

Finally, we directly tested whether the two groups differed in theta modulation related to instrumental learning bias. Group was entered as a between‑subjects factor in a 2 (group: HC vs. OCD) × 2 (outcome: reward vs. punishment) × 2 (response: Go vs. NoGo) mixed repeated‑measures ANOVA. The main effect of outcome was significant (*F*(1, 71) = 34.81, *p* < 0.001, *η_p_²* = 0.33), indicating that punishment elicited stronger theta activity than reward. The main effect of response showed a marginal trend (*F*(1, 71) = 3.71, *p* = 0.058, *η_p_²* = 0.05), whereas the main effect of group was not significant (*F*(1, 71) = 0.84, *p* = 0.361, *η_p_²* = 0.01). The critical interaction between outcome and response, which captures the contrast between biased and unbiased learning, was not significant (*F*(1, 71) = 2.51, *p* = 0.117, *η_p_²* = 0.03), indicating no significant theta modulation related to biased versus unbiased learning. The three‑way interaction between outcome, response, and group was also not significant (*F*(1, 71) = 0.27, *p* = 0.608, *η_p_²* = 0.004), suggesting that the two groups did not differ in this bias‑related theta modulation. Furthermore, the interaction between response and group was not significant (*F*(1, 71) = 0.01, *p* = 0.932, *η_p_²* = 0.001), indicating that the groups did not differ in theta modulation related to relatively enhanced versus reduced learning. Analyses based on difference scores yielded the same pattern. The group difference in the biased‑minus‑unbiased difference score was not significant (*t*(71) = −0.52, *p* = 0.608, Cohen’s *d* = −0.12), nor was the group difference in the relatively‑enhanced‑minus‑reduced difference score (*t*(71) = 0.09, *p* = 0.932, Cohen’s *d* = 0.02).

Overall, the feedback‑locked midfrontal theta results suggested that theta activity during feedback processing was primarily sensitive to feedback outcome, with non‑preferred outcomes, particularly punishment, eliciting stronger theta activity. In the HC group, non‑preferred outcomes elicited enhanced theta activity in both win‑cue and avoid‑cue contexts, suggesting that healthy individuals consistently differentiated unfavorable outcomes, including reward omission and punishment. In the OCD group, non‑preferred outcomes also elicited stronger theta activity overall. However, this effect was mainly driven by increased theta activity to punishment in the avoid‑cue context. In contrast, for win cues, the OCD group did not significantly differentiate reward from neutral outcomes. This pattern suggested that the sensitivity in OCD to outcome differences in reward contexts may be relatively weaker. Nevertheless, further group‑level analyses revealed no significant group differences, indicating that the present findings do not provide sufficient evidence for reliable between‑group differences in feedback‑related theta activity or in theta representations related to instrumental learning biases. Taken together, these findings suggested that feedback‑locked theta activity primarily reflects the processing of feedback, especially non‑preferred outcomes, rather than neural modulation specifically associated with instrumental learning bias.

**Text F.** **Single‑trial midfrontal‑lateral prefrontal and midfrontal‑motor theta phase synchrony**

In the main text, we showed that motivational conflict modulated trial‑averaged phase synchrony between the midfrontal cortex (MFC) and lateral prefrontal cortex (lPFC) regions. Here, we tested whether the same pattern could also be observed at the single‑trial level before these neural measures were entered into the computational models. Single‑trial intersite phase synchrony (ISPS) was analyzed with mixed‑effects regression models. For the midfrontal‑lateral prefrontal cortex analysis, the model included valence (win versus avoid) and required action (Go versus NoGo) as within‑subject factors. All models included a full random‑effects structure. Before model fitting, the phase synchrony values were mean‑corrected and inverse transformed to improve normality.

In the healthy control group, the single‑trial midfrontal‑lPFC analysis showed a significant valence × required action interaction (β = −0.0009, 95% CI [−0.001, −0.0003], χ²(1) = 8.20, *p* = 0.004). The main effect of required action was not significant (β = −0.0004, 95% CI [−0.001, 0.0002], χ²(1) = 1.83, *p* = 0.18), and the main effect of valence was also not significant (β = 0.0005, 95% CI [−0.0001, 0.001], χ²(1) = 2.63, *p* = 0.11). Follow‑up analyses showed that the valence effect was not significant in Go trials (β = −0.0003, 95% CI [−0.001, 0.0005], χ²(1) = 0.60, *p* = 0.44), but was significant in NoGo trials (β = 0.001, 95% CI [0.0005, 0.002], χ²(1) = 9.70, *p* = 0.002). From the action perspective, phase synchrony was higher for NoGo than for Go responses under win cues (β = −0.001, 95% CI [−0.002, −0.0004], χ²(1) = 8.49, *p* = 0.004), whereas the action effect was not significant under avoid cues (β = 0.0004, 95% CI [−0.0004, 0.001], χ²(1) = 0.96, *p* = 0.33). These results indicate that, in healthy controls, single‑trial midfrontal‑lPFC synchrony was increased under motivational conflict, with the clearest effect in NoGo trials.

A similar overall pattern was found in the OCD group. The valence × required action interaction was significant (β = −0.0008, 95% CI [−0.001, −0.0002], χ²(1) = 6.25, *p* = 0.01), while neither the main effect of required action (β = −0.0003, 95% CI [−0.001, 0.0003], χ²(1) = 0.79, *p* = 0.37), nor the main effect of valence (β = 0.0004, 95% CI [−0.0003, 0.001], χ²(1) = 1.17, *p* = 0.28), reached significance. The simple‑effect analyses showed that the valence effect was not significant in Go trials (β = −0.0004, 95% CI [−0.001, 0.0004], χ²(1) = 0.94, *p* = 0.33), but was significant in NoGo trials (β = 0.001, 95% CI [0.0002, 0.002], χ²(1) = 5.83, *p* = 0.02). When the data were examined from the action side, the action effect under win cues was significant (β = −0.001, 95% CI [−0.002, −0.0002], χ²(1) = 5.69, *p* = 0.02), and the action effect under avoid cues was not significant (β = 0.0005, 95% CI [−0.0004, 0.001], χ²(1) = 1.31, *p* = 0.25). Thus, the OCD group also showed a reliable conflict‑related modulation of single‑trial midfrontal‑lPFC synchrony, again driven mainly by NoGo trials.

Overall, the single‑trial midfrontal‑lPFC phase synchrony results reproduced the main conflict‑related pattern observed at the trial‑averaged level. In both groups, the critical effect was a significant valence × required action interaction, and the simple‑effect analyses showed that this pattern was primarily driven by NoGo trials. These findings suggested that the trial‑by‑trial synchrony estimates captured meaningful variation in conflict‑related prefrontal coordination and were therefore suitable for the subsequent computational modeling analyses.

In addition to the MFC‑motor ISPS effects at non‑executing motor sites reported in the main text, we examined whether conflict‑related synchrony depended on the functional state of the motor region. The contralateral motor cortex during Go responses was defined as the executing site, whereas the ipsilateral motor cortex during Go responses and the bilateral motor cortex during NoGo responses were treated as non‑executing sites. This distinction was motivated by the framework of activity‑dependent functional connectivity [10], according to which the strength of midfrontal coupling with downstream regions may depend on the current task‑related activation state of the target region.

In the HC group, a 2 (valence) × 3 (response type: contra‑Go, ipsi‑Go, NoGo) repeated‑measures ANOVA revealed a significant valence × response type interaction (*F*(2,35) = 4.32, *p* = 0.021, *η_p_²*=0.20). When the data were recoded into a 2 (congruency) × 2 (motor execution) design, the interaction remained significant (*F*(1,36) = 4.82, *p* = 0.035, *η_p_²*=0.12), indicating that the conflict‑related synchrony effect differed between executing and non‑executing motor sites. As reported in the main text, this pattern was characterized by a reliable conflict‑related increase at non‑executing sites. In contrast, neither the valence × response type interaction (*F*(2,28) = 1.02, *p* = 0.38, *η_p_²*=0.07) nor the congruency × motor execution interaction (*F*(1,29) = 0.03, *p* = 0.86, *η_p_²*=0.001) was significant in the OCD group. Thus, the supplementary analyses indicated that healthy controls showed an execution‑dependent organization of conflict‑related MFC‑motor synchrony, whereas this functional differentiation was absent in the OCD group.

We next asked whether this execution‑dependent pattern could also be detected at the single‑trial level before these neural measures were entered into the computational models. Single‑trial ISPS was analyzed using mixed‑effects regression models. For the midfrontal–motor analysis, synchrony was modeled across valence and motor‑site conditions, and planned contrasts were used to quantify the effects of motivational congruency as a function of motor execution. Here, the congruency contrast corresponds to the valence × required action interaction used in the other analyses. All models included a full random‑effects structure.

In the HC group, the single‑trial analysis showed an execution‑dependent conflict effect. The congruency × motor execution interaction was significant (β = 0.014, 95% CI [0.0002, 0.029], χ²(1) = 3.96, *p* = 0.047), indicating that conflict‑related modulation differed between executing and non‑executing motor sites. Follow‑up contrasts showed a significant conflict‑related increase in synchrony at non‑executing sites (β = 0.020, 95% CI [0.008, 0.032], χ²(1) = 10.20, *p* < 0.01), whereas the congruency effect at executing sites was not significant (β = −0.005, 95% CI [−0.014, 0.003], χ²(1) = 1.46, *p* = 0.23). The overall main effect of valence showed only a trend (β = 0.014, 95% CI [−0.0006, 0.029], χ²(1) = 3.56, *p* = 0.06). Together, these results indicated that conflict‑related MFC‑motor synchrony was preferentially expressed at non‑executing motor sites in healthy controls, reproducing the execution‑dependent pattern observed in the trial‑averaged analyses.

However, in the OCD group, the congruency × motor execution interaction was not significant (β = 0.032, 95% CI [−0.044, 0.108], χ²(1) = 0.67, *p* = 0.41). The simple congruency effect was not significant at executing sites (β = 0.009, 95% CI [−0.034, 0.053], χ²(1) = 0.18, *p* = 0.67), or at non‑executing sites (β = 0.023, 95% CI [−0.041, 0.086], χ²(1) = 0.49, *p* = 0.48). The main effect of valence was also not significant (β = 0.013, 95% CI [−0.063, 0.088], χ²(1) = 0.11, *p* = 0.74). Thus, people with OCD showed no reliable single‑trial conflict‑related modulation of MFC‑motor synchrony and no evidence that such modulation differed as a function of motor execution.

Taken together, the trial‑averaged and single‑trial analyses converged on an execution‑dependent pattern of MFC‑motor synchrony. In healthy controls, conflict‑related synchrony was most clearly expressed at non‑executing motor sites, whereas no reliable conflict‑related enhancement was observed at executing sites. This functional differentiation was absent in the OCD group, suggesting that the coordination of midfrontal control signals with motor regions depends on their task‑related functional state, consistent with the framework of activity‑dependent functional connectivity [10]. One possible account is that reward cues promote action readiness by engaging basal ganglia Go pathways [15–17] and increasing motor‑cortical activation [18], thereby altering the responsiveness of executing motor regions to midfrontal theta input; this interpretation is also consistent with evidence that reward facilitates behavioral activation [19].

Importantly, these analyses and the subsequent computational modeling addressed complementary questions. The trial‑averaged and single‑trial analyses characterized where conflict‑related MFC‑motor synchrony was most clearly expressed, whereas the computational models tested whether trial‑by‑trial contralateral MFC‑motor synchrony, which is most directly linked to action implementation, tracked variation in motivational biases. Thus, the stronger average conflict‑related effect at non‑executing sites does not conflict with the use of contralateral motor synchrony as the behaviorally relevant modulation signal in the computational models.

**Text G. Evaluation of midfrontal theta phase‑synchrony modulation of instrumental contribution and Pavlovian bias within a single model**

In the main analysis, phase‑synchrony effects on instrumental contribution and Pavlovian bias were initially evaluated using separate, best‑fitting synchrony models. This approach allowed us to identify the dominant synchrony pathway associated with each computational component. However, because the two subject‑level beta parameters were subsequently plotted in a common coordinate space, a key concern is whether these separately estimated effects capture overlapping variance. To address this issue, we fitted additional joint theta phase‑synchrony models, in which the synchrony effects on both instrumental contribution and Pavlovian bias were estimated simultaneously within the same model likelihood.

The present supplementary models were built on the same reinforcement-learning architecture described in the main Materials and Methods section. Therefore, we do not repeat the full softmax choice rule and learning‑update equations here. In these models, midfrontal theta phase‑synchrony effects on instrumental contribution and Pavlovian bias were evaluated simultaneously within a single model likelihood, rather than being estimated in separate models. The synchrony‑modulated terms were applied only to motivationally conflicting trials. In the HC model (M6), MFC‑lPFC phase synchrony scaled the instrumental action values, whereas MFC‑motor phase synchrony scaled Pavlovian bias. In the OCD model (M7), MFC‑motor phase synchrony scaled both the instrumental action values and Pavlovian bias.

In the HC model (M6), MFC‑lPFC phase synchrony scaled the instrumental contribution for both Go and NoGo responses on conflict trials. For Go responses, Pavlovian bias was additionally modulated by contralateral MFC‑motor phase synchrony. In contrast, NoGo responses were affected only by the synchrony‑modulated instrumental Q‑value term, without the Go bias or Pavlovian bias terms. On non‑conflict trials, the model reduced to its standard form, with no trial‑level synchrony modulation.

| $\begin{matrix} w_{i}(Go,s_{t})=\left\{\begin{matrix} \left(1-\beta_{\text{Q},\text{lPFC},i}*ISPS_{\text{lPFC},i,t}\right)\text{Q}_{i}(Go,s_{t})+\left(\pi_{i}+\beta_{\pi,\text{motor},i}*ISPS_{\text{motor-contra},i,t}\right)V(s_{t})+b_{i}, & if conflict \\ \text{Q}_{i}(Go,s_{t})+\pi_{i}V(s_{t})+b_{i}, & else \end{matrix}\right. \\ w_{i}(NoGo,s_{t})=\left\{\begin{matrix} \left(1-\beta_{\text{Q},\text{lPFC},i}*ISPS_{\text{lPFC},i,t}\right)\text{Q}_{i}(NoGo,s_{t}), & if conflict \\ \text{Q}_{i}(NoGo,s_{t}), & else \end{matrix}\right. \end{matrix}$ | (4) |
| --- | --- |

In this model, β_Q,lPFC_ < 0 indicates that stronger MFC‑lPFC phase synchrony increases the instrumental contribution during choice, whereas β_Q,lPFC_ > 0 indicates reduced instrumental contribution. Similarly, β_π,motor_ < 0 indicates that stronger MFC‑motor phase synchrony suppresses Pavlovian bias, whereas β_π,motor_ > 0 indicates amplification of Pavlovian bias. Therefore, negative values of both parameters would reflect an adaptive regulatory pattern characterized by enhanced instrumental control and reduced Pavlovian response tendencies, whereas positive values would indicate weaker instrumental contribution or stronger Pavlovian response tendencies.

In the OCD model (M7), MFC‑motor phase synchrony scaled the instrumental contribution for Go responses on conflict trials. Specifically, the contralateral MFC‑motor synchrony term modulated the instrumental contribution for left and right Go responses. For Go responses, Pavlovian bias was additionally modulated by the same contralateral MFC‑motor phase‑synchrony pathway. In contrast, for NoGo responses, the model retained the standard instrumental contribution without adding a phase‑synchrony modulation term, because the MFC‑motor synchrony effect on Q was implemented in a side‑specific manner for left and right Go responses.

| $\begin{matrix} w_{i}(Go,s_{t})=\left\{\begin{matrix} \left(1-\beta_{\text{Q},\text{motor},i}*ISPS_{\text{motor-contra},i,t}\right)\text{Q}_{i}(Go,s_{t})+\left(\pi_{i}+\beta_{\pi,\text{motor},i}*ISPS_{\text{motor-contra},i,t}\right)V(s_{t})+b_{i}, & if conflict \\ \text{Q}_{i}(Go,s_{t})+\pi_{i}V(s_{t})+b_{i}, & else \end{matrix}\right. \\ w_{i}(NoGo,s_{t})=\left\{\begin{matrix} \text{Q}_{i}(NoGo,s_{t}), & if conflict \\ \text{Q}_{i}(NoGo,s_{t}), & else \end{matrix}\right. \end{matrix}$ | (5) |
| --- | --- |

In this model, β_Q,motor_ < 0 indicates that stronger MFC‑motor phase synchrony increases the effective instrumental contribution for Go responses during choice, whereas β_Q,motor_ > 0 indicates reduced instrumental contribution. Similarly, β_π,motor_ < 0 indicates that stronger MFC‑motor phase synchrony suppresses Pavlovian bias, whereas β_π,motor_ > 0 indicates amplification of Pavlovian bias. Thus, negative values of both parameters would reflect an adaptive regulatory pattern characterized by enhanced instrumental control and reduced Pavlovian response tendencies, whereas positive values would indicate weaker instrumental contribution or stronger Pavlovian influence.

To evaluate whether the individual regulatory‑orientation parameters remained stable when the two synchrony‑related effects were estimated within the same model likelihood, we correlated subject‑level β estimates from the original single‑effect models with the corresponding estimates from the joint-model analyses. Across all four comparisons, the correlations were strong and positive (*r=*0.80–0.99*,* all *p<*0.001). As shown in Fig F, in the HC group, β estimates indexing MFC‑lPFC modulation of the weighting of instrumental contribution were highly consistent between the original M5a and the M6 model, and β estimates indexing MFC‑motor synchrony modulation of Pavlovian bias were highly consistent between the original M5d and the M6 model. Similarly, in the OCD group, β estimates indexing MFC‑motor synchrony modulation of the weighting of instrumental contribution and Pavlovian bias were strongly correlated between the original single‑effect models and the M7 model.

**Fig F. Consistency of subject‑level synchrony‑modulation β estimates between single‑effect and joint models.** To address whether synchrony‑related modulation of instrumental action‑value weighting and Pavlovian bias remained stable when estimated within a single model, we correlated subject‑level β estimates from the original single‑effect models with the corresponding β estimates from the joint models. **(A)** In healthy controls, instrumental action‑value β estimates for MFC‑lPFC synchrony‑related Q modulation were strongly correlated between the original M5a model and the joint M6 model. **(B)** Pavlovian‑bias β estimates for MFC‑motor synchrony‑related π modulation were strongly correlated between the original M5d model and the joint M6 model. **(C)** In the OCD group, instrumental action‑value β estimates for MFC‑motor synchrony‑related Q modulation were positively correlated between the original M5b model and the joint M7 model. **(D)** Pavlovian‑bias β estimates for MFC‑motor synchrony‑related π modulation were strongly correlated between the original M5d model and the joint M7 model. Each dot represents one participant. Lines indicate linear fits, shaded areas indicate 95% confidence intervals, and Pearson’s *r* and *p* values are shown above each panel. These results indicate that the individual differences captured by the original single‑effect synchrony models were largely preserved when the effects on Q weighting and Pavlovian bias were estimated simultaneously within the joint models. The numerical data underlying panels A–D can be found in S6 Data.

These results provide an important robustness check for the regulatory‑orientation analysis. If the two synchrony‑modulation terms had captured largely redundant variance, estimating them simultaneously within a single model likelihood would be expected to alter the subject‑level estimates, for instance by attenuating effects, reversing their signs, or substantially reordering individuals. Instead, the high cross‑model consistency indicates that the individual differences captured by the original single‑effect models were largely preserved under simultaneous estimation.

To further validate the regulatory‑orientation analysis, we extracted the subject‑level β estimates from the jointly estimated model. In these analyses, the synchrony effects on instrumental contribution and Pavlovian bias were estimated simultaneously within the same model likelihood. These jointly estimated parameters were then projected into the same two‑dimensional regulatory‑orientation space. As shown in Fig G, the resulting spatial distributions closely resembled the original visualization derived from the single‑effect models. For healthy controls, the joint‑model estimates preserved the complementary regulatory pattern. Individuals were distributed toward the orientation reflecting stronger instrumental contribution together with stronger suppression of Pavlovian bias. For people with OCD, the same analysis preserved the more motor‑dominant and heterogeneous distribution observed in the original regulatory‑orientation space. This consistency suggests that the spatial organization of individual regulatory orientations was unlikely to be driven solely by plotting parameters obtained from separate models. The group‑specific distributional pattern remained stable when the two synchrony‑related modulation effects were estimated within the same model likelihood. We further examined whether the clinical association between regulatory orientation and obsessive‑thought severity was preserved when Φ_reg_ was derived from the jointly estimated synchrony‑modulation parameters. Consistent with the original analysis, Φ_reg_ remained positively associated with Yale-Brown Obsessive Compulsive Scale (Y-BOCS) obsession severity after controlling for anxiety and depression (*r* = 0.40, *p* = 0.017). This association also remained significant after additionally controlling for trait impulsivity (*r* = 0.35, *p* = 0.037). Thus, the symptom‑related association observed in the original regulatory‑orientation analysis was retained when the two synchrony‑related modulation effects were estimated within the same model likelihood. These findings provide an additional robustness check, suggesting that the clinically meaningful link between regulatory orientation and obsessive‑thought severity was not driven solely by extracting parameters from separate single‑effect models.

**Fig G. Subject‑level regulatory orientation of jointly estimated midfrontal theta phase‑synchrony modulation parameters.** Individual regulatory orientation of motivational‑bias modulation in healthy controls **(A)** and people with OCD **(B)**, based on subject‑level β estimates extracted from the joint theta phase‑synchrony models. In contrast to the original single‑effect models, the present visualization uses parameters jointly estimated within the same model likelihood: M6 for healthy controls and M7 for people with OCD. Each point represents one participant’s jointly estimated synchrony‑modulation parameters. For Pavlovian bias, the x‑axis represents the MFC‑motor phase synchrony modulation parameter, β_ISPS‑motor_, indexing modulation of Pavlovian bias. The y‑axis represents instrumental action‑values weighting, but the specific synchrony pathway differed by group according to the group‑specific joint model structure. In healthy controls, the y‑axis represents β_ISPS‑lPFC_, indexing MFC‑lPFC phase synchrony modulation of instrumental action‑values weighting. In the OCD group, the y‑axis represents β_ISPS‑motor_, indexing MFC‑motor phase synchrony modulation of instrumental action‑values weighting. For interpretation, β < 0 on the x‑axis indicates stronger suppression of Pavlovian bias, whereas β < 0 on the y‑axis indicates stronger weighting of instrumental action values during choice. Black dots indicate individual participants, background contours show the two‑dimensional kernel density distribution, white triangles mark the group mean, and dashed lines indicate zero‑effect reference axes. The numerical data underlying panels A–B can be found in S6 Data.

Overall, the joint‑model simultaneous analyses provide an important sensitivity check that supports the robustness of the original β‑based regulatory‑orientation visualization. Although the primary coordinate space was constructed using parameters extracted from separate single‑effect synchrony models, the group‑specific distributional pattern was largely preserved when the relevant β terms were re‑estimated concurrently within a shared model likelihood and then projected into the same space. This topographic consistency suggests that individual regulatory tendencies remained stable across model specifications and were not substantially distorted by parameter trade‑offs or dilution in the joint-model analysis. Accordingly, we treat this verification as a supplementary methodological validation that complements rather than replaces the main model specifications. These results support the robustness of the individual‑difference patterns underlying midfrontal theta phase‑synchrony modulation of motivational biases.

**SI References**

1. Guo Z, Yun S, Xie Z, Jia S, Liu C, Ma X, et al. The effect of an 8-week cognitively engaging exercise intervention on conflict inhibition in college students with depressive symptoms: a randomized controlled trial. *Scand J Med Sci Sports*. 2026;36(5):e70295. doi: [10.1111/sms.70295](https://doi.org/10.1111/sms.70295).
2. Bertram FE, Leue A. The neurocognitive basis of deception: individual differences of N2‑related conflict monitoring in a social deception task. *Behav Brain Res*. 2025;494:115738. doi: [10.1016/j.bbr.2025.115738](https://doi.org/10.1016/j.bbr.2025.115738).
3. Wang K, Cai S, Huang T, Deng Z, Qian J, Chen Y, et al. Unveiling the neural mechanisms of acute aerobic exercise on inhibitory control among young adults with obesity: insights from an ERP study. *Acta Psychol (Amst)*. 2024;250:104506. doi: [10.1016/j.actpsy.2024.104506](https://doi.org/10.1016/j.actpsy.2024.104506).
4. Knežević M. Temporal course of interference control from early to late young adulthood: an ERP study. *Brain Sci*. 2024;14(6):536. doi: [10.3390/brainsci14060536](https://doi.org/10.3390/brainsci14060536).
5. Dumsky J, Maier ME, Steinhauser M. Effects of the number of competing responses on neural signatures of pre‑ and post‑response conflict. *Biol Psychol*. 2023;182:108643. doi: [10.1016/j.biopsycho.2023.108643](https://doi.org/10.1016/j.biopsycho.2023.108643).
6. Riesel A, Klawohn J, Kathmann N, Endrass T. Conflict monitoring and adaptation as reflected by N2 amplitude in obsessive‑compulsive disorder. *Psychol Med*. 2017;47(8):1379–1388. doi: [10.1017/S0033291716003597](https://doi.org/10.1017/S0033291716003597).
7. Koorenhof LJ, Dommett EJ. An investigation into response inhibition in distinct clinical groups within obsessive‑compulsive disorder. *J Neuropsychiatry Clin Neurosci*. 2019;31(3):228–238. doi: [10.1176/appi.neuropsych.18070166](https://doi.org/10.1176/appi.neuropsych.18070166).
8. Ruchsow M, Reuter K, Hermle L, Ebert D, Kiefer M, Falkenstein M. Executive control in obsessive‑compulsive disorder: event‑related potentials in a Go/Nogo task. *J Neural Transm (Vienna)*. 2007;114(12):1595–1601. doi: [10.1007/s00702-007-0779-4](https://doi.org/10.1007/s00702-007-0779-4).
9. Suzuki T, Gu P, Grove TB, Hammond T, Collins KM, Pamidighantam P, et al. Abnormally enhanced midfrontal theta activity during response monitoring in youths with obsessive‑compulsive disorder. *Biol Psychiatry*. 2023;93(11):1031–1040. doi: [10.1016/j.biopsych.2022.10.020](https://doi.org/10.1016/j.biopsych.2022.10.020).
10. Cohen MX. A neural microcircuit for cognitive conflict detection and signaling. *Trends Neurosci.* 2014;37(9):480–490. doi: [10.1016/j.tins.2014.06.004](https://doi.org/10.1016/j.tins.2014.06.004).
11. Pandey S, Osinsky R. Beyond uniformity: individual sensitivities to reward and punishment shape midfrontal‑theta responses to approach avoidance conflict. *Soc Cogn Affect Neurosci*. 2025;20(1):nsaf114. doi: [10.1093/scan/nsaf114](https://doi.org/10.1093/scan/nsaf114).
12. van de Vijver I, Ridderinkhof KR, Cohen MX. Frontal oscillatory dynamics predict feedback learning and action adjustment. *J Cogn Neurosci*. 2011;23(12):4106–4121. doi: [10.1162/jocn_a_00110](https://doi.org/10.1162/jocn_a_00110).
13. Liu M, Dong W, Qin S, Verguts T, Chen Q. Electrophysiological signatures of hierarchical learning. *Cereb Cortex*. 2022;32(3):626–639. doi: [10.1093/cercor/bhab245](https://doi.org/10.1093/cercor/bhab245).
14. Zhang Y, Deng F, Liao J, Verguts T, Chen Q. Medial frontal theta reduction impairs rule switching via prediction error. *J Neurosci*. 2026;46(1):e1236252025. doi: [10.1523/JNEUROSCI.1236-25.2025](https://doi.org/10.1523/JNEUROSCI.1236-25.2025).
15. Collins AG, Frank MJ. Opponent actor learning (OpAL): modeling interactive effects of striatal dopamine on reinforcement learning and choice incentive. Psychol Rev. 2014;121(3):337–366. doi: [10.1037/a0037015](https://doi.org/10.1037/a0037015).
16. Hernández-López S, Bargas J, Surmeier DJ, Reyes A, Galarraga E. D1 receptor activation enhances evoked discharge in neostriatal medium spiny neurons by modulating an L‑type Ca2+ conductance. *J Neurosci*. 1997;17(9):3334–3342. doi: [10.1523/JNEUROSCI.17-09-03334.1997](https://doi.org/10.1523/JNEUROSCI.17-09-03334.1997).
17. Hernández-López S, Tkatch T, Perez‑Garci E, Galarraga E, Bargas J, Hamm H, et al. D2 dopamine receptors in striatal medium spiny neurons reduce L‑type Ca2+ currents and excitability via a novel PLCβ1–IP3–calcineurin-signaling cascade. *J Neurosci*. 2000;20(24):8987–8995. doi: [10.1523/JNEUROSCI.20-24-08987.2000](https://doi.org/10.1523/JNEUROSCI.20-24-08987.2000).
18. Chiu YC, Cools R, Aron AR. Opposing effects of appetitive and aversive cues on go/no‑go behavior and motor excitability. *J Cogn Neurosci*. 2014;26(8):1851–1860. doi: [10.1162/jocn_a_00585](https://doi.org/10.1162/jocn_a_00585).
19. Guitart‑Masip M, Duzel E, Dolan R, Dayan P. Action versus valence in decision making. *Trends Cogn Sci*. 2014;18(4):194–202. doi: [10.1016/j.tics.2014.01.003](https://doi.org/10.1016/j.tics.2014.01.003).
