# Supplementary material for: Impaired midfrontal‑motor theta phase synchronization characterizes maladaptive motivational behavior in people with obsessive‑compulsive disorder
Source: PLoS Biol. 2026 Sep 1;24(9):e3003979. doi: 10.1371/journal.pbio.3003979 (PMC13568527; doi:10.1371/journal.pbio.3003979)
Supplement: Supplementary file 2 [file pbio.3003979.s010.docx]

**Summary**

This manuscript investigates the neural and computational mechanisms underlying maladaptive motivational biases in OCD. Using a motivational Go/NoGo task combined with computational modelling and EEG, the authors compare 36 OCD patients with 37 HC. They report that OCD is characterized by increased Pavlovian bias and reduced learning rates. While midfrontal theta power (associated with conflict detection) appears preserved, its temporal dynamics are altered, overlapping with response execution. Critically, the authors identify impaired midfrontal–motor theta phase synchrony as a key mechanism underlying ineffective modulation of motivational biases, proposing this as a neural substrate of the “insight–action dissociation” in OCD.

The study combines computational psychiatry and electrophysiology in a sophisticated way and addresses an important theoretical question. However, several conceptual, methodological, and interpretational issues limit the strength of the conclusions.

**Major Comments**

**1. Causal claims vs correlational design**

The manuscript frequently makes mechanistic and quasi-causal claims (e.g., that impaired MFC–motor synchrony drives maladaptive behaviour and constitutes a treatment target). However, all analyses are correlational.
- The data show associations between ISPS and behaviour/model parameters—not causation. The wording “drives maladaptive motivational behaviour” (including in the title) is too strong.

Recommendation:

- Temper claims throughout (title, abstract, discussion). Consider framing as:
- “is associated with”
- “may contribute to”
- “is consistent with a mechanism involving…”

**2. Interpretation of delayed theta = impaired control**

The key claim is that theta timing overlap with response reflects delayed control in OCD.

Concerns:

- Reaction time differences and variability may confound this interpretation.
- Overlap with response does not necessarily imply functional inefficacy. It could reflect prolonged processing, motor contamination, or different strategy.
- No direct test linking trial-level theta timing → behavioural success is presented.

Recommendation:

- Provide trial-level analyses linking pre-response theta vs performance.
- Control explicitly for RT variability.
- Consider alternative interpretations

**3. Interpretation of heterogeneity**

The identification of two regulatory subtypes in OCD is interesting but currently underdeveloped.

Concerns:

- Subgroups appear post hoc and descriptive.
- No clustering method or statistical validation is reported.
- The “two orientations” may reflect a continuous distribution.

Recommendation:

- Apply formal clustering or mixture modelling.
- Test whether subgroups are statistically separable.
- Clarify whether the angular metric (Φreg) captures a continuum rather than discrete types.

**4. Sample size and analytical complexity**

While the sample size is reasonable, the study includes:

- hierarchical modelling
- time-frequency analyses
- connectivity analyses
- individual-difference modelling

This raises risk of overfitting and inflated false positives.

Recommendation:

- Discuss statistical power and robustness.
- Consider cross-validation or split-sample validation.

**Minor Concerns**

- Clarify medication status of OCD participants (potential confound).
- Report comorbidities more explicitly.
- Some terminology shifts between “instrumental learning bias” and “instrumental contribution”—define clearly and consistently.
- The introduction could better distinguish Pavlovian vs instrumental vs habit frameworks

**Strengths**

- Strong integration of computational modelling + EEG
- Addresses an important and clinically relevant question
- Innovative attempt to link network synchrony to computational parameters
- Data and code availability (GitHub) is excellent
- The heterogeneity angle is promising and worth further development

**Recommendation: Major revision**

The study is potentially impactful but currently overstates its conclusions and requires more cautious interpretation.
