# Supplementary material for: Impaired midfrontal‑motor theta phase synchronization characterizes maladaptive motivational behavior in people with obsessive‑compulsive disorder
Source: PLoS Biol. 2026 Sep 1;24(9):e3003979. doi: 10.1371/journal.pbio.3003979 (PMC13568527; doi:10.1371/journal.pbio.3003979)
Supplement: Supplementary file 3 [file pbio.3003979.s011.docx]

**To the Editor,**

We are very grateful to you for the careful evaluation of our manuscript and to all reviewers for their constructive and detailed comments. Below, we provide a point‑by‑point response to all comments. We have revised the manuscript accordingly and believe that these changes have strengthened the work.

**⮚Reviewer #1**

**1. Major:**

(1) It is very helpful to introduce the “insight‑action dissociation”, but it remains less frequently mentioned in the remaining of the manuscript. It'd be ideal to refer to back to it in the Discussion section to reconcile all the findings on a bigger picture.

**⚫ Reply:**

We agree that the insight‑action dissociation provides a powerful framework to unify our findings across behavioral, computational, and neurophysiological levels. Following this guidance, we have revised the discussion section to systematically contextualize our results within this clinical paradox. Instead of treating it as a descriptive label, we now utilize our multi‑level findings to offer a comprehensive, mechanistic explanation of how the cognitive awareness of an obsession becomes decoupled from motor execution. Specifically, we have woven this conceptual thread through the major subsections of the Discussion to reconcile our findings into a larger cohesive picture along three distinct dimensions.

First, at the behavioral and computational level, we introduce the algorithmic foundation of this dissociation. Our behavioral modeling shows that although individuals with obsessive‑compulsive disorder successfully learn and represent fundamental task rules, their final decisions remain heavily hijacked by rigid, cue‑driven Pavlovian biases when motivational conflict arises. This specific configuration of computational parameters provides a formal definition of the dissociation, demonstrating that intact value representation is systematically overridden by a heightened Pavlovian bias parameter at the choice policy level. In the main text, we have added the following sentence (**Page 33 line 669–673**):

*“The computational phenotype provides an algorithmic account of the “insight‑action dissociation” in OCD. OCD patients were able to learn and represent the basic task rules, but when Pavlovian biases conflicted with instrumental requirements, their choices remained strongly biased by rigid, cue‑driven Pavlovian tendencies.”*

Second, at the local neurophysiological level, we map the intact cognitive awareness or insight component to its neural substrates. Our finding of preserved conflict*‑*related midfrontal theta power in patients establishes that the midfrontal cortex remains fully capable of monitoring motivational conflict and generating a robust control‑demand signal. This intact local signaling serves as a direct neural correlate of the clinical observation that patients retain explicit cognitive insight into the irrationality of their symptoms, meaning the breakdown does not originate from a failure to detect the need for control. We made the following revisions to the main text to clearly convey this point:

*“Therefore, preserved local conflict‑related midfrontal theta signaling may reflect a neural correlate of the preserved “insight” component of the dissociation. It demonstrates that OCD patients can detect motivational conflict and generate a control‑demand signal, consistent with the clinical observation that patients possess explicit insight into the irrationality of their obsessions and compulsive behaviors.” (****Page 34 line 682‑687****)*

*“Given that local midfrontal theta reflected preserved conflict detection, the critical question for understanding “insight‑action dissociation” shifts from whether control demands are registered to how this generated signal is communicated to downstream functional networks.” (****Page 34 line 688‑691****)*

Third, at the long‑range network level, we delineate the network transmission failure that prevents intact insight from gating behavior. We discuss how the critical question shifts from local detection to interregional communication efficacy. Our results show that while conflict signals are relayed to the lateral prefrontal cortex via an inefficient compensatory pathway, the key transmission deficit resides in the pathway connecting midfrontal and motor structures. The combined effects of altered local motor excitability and weakened midfrontal‑motor phase synchrony prevent the top‑down control blueprint from being successfully implemented at the execution level, leaving automated Pavlovian impulses uninhibited. See the following additions to the manuscript conveying this point:

*“Together, disrupted local excitability and weakened long‑range coupling may undermine rhythmic coordination and inhibitory control, providing a network‑level account of the “insight‑action dissociation”, which explains why compulsive behaviors still persist even when cognitive capacity remains intact.” (****Page 36 line 730–734****)*

*“Together, these results imply that OCD patients could not effectively gate motor output or correct cue‑driven impulses at the action level, and offers a mechanistic account of the “insight‑action dissociation” in OCD, in which preserved conflict awareness is decoupled from effective action implementation.” (****Page 40 line 813–817****)*

We believe that these extensive additions convey to the reader how our computational and neural findings together provide a comprehensive and mechanistic account of the insight‑access dissociation in OCD.

(2) I am not entirely sure why the cortico‑striato‑thalamo‑cortical (CSTC) circuits are mentioned in the Intro. The current method is EEG, and the CSTC is not directly examined. Perhaps it could be discussed in the wider literature in the Discussion rather than the Intro (or, it can be mentioned in the Intro, but it needs to be better motivated/justified.)

⚫ **Reply:**

We thank the reviewer for this important and constructive suggestion. In the original Introduction, our rationale for mentioning cortico‑striato‑thalamo‑cortical (CSTC) circuits was to provide a neurobiological background for abnormal motivational biases in OCD. Specifically, previous studies have shown that OCD is associated with structural and functional connectivity abnormalities in CSTC circuits, and these circuits are closely involved in reward and punishment sensitivity, motivational salience, habit formation, and goal‑directed control. Therefore, we originally used this literature to support the possibility that Pavlovian response bias and instrumental learning bias may be altered in OCD.

However, we fully agree with the reviewer that the present study did not directly examine CSTC circuitry, and that an extended discussion of CSTC circuits in the Introduction could overstate the neural mechanisms tested in the current EEG study. To avoid this potential misunderstanding, we substantially revised the Introduction by removing the detailed CSTC‑related paragraph and replacing it with a more behaviorally grounded theoretical rationale. The revised Introduction now focuses on heightened sensitivity to symptom‑relevant cues, elevated harm‑avoidance motivation, negative reinforcement of safety‑seeking behaviors, and the gradual transition from goal‑directed safety behaviors to cue‑triggered habitual responses. Based on this framework, we hypothesized that OCD patients would exhibit enhanced Pavlovian response tendencies driven by salient cues, together with an instrumental learning bias characterized by abnormal updating of action‑outcome contingencies.

The revised Introduction now reads as follows:

*“Recent OCD research suggests that heightened sensitivity to symptom‑relevant cues and elevated harm‑avoidance motivation promote avoidance or safety‑seeking responses [37,38], such as washing or checking. At the same time, these actions are negatively reinforced because they temporarily reduce anxiety or feelings of incompleteness [39,40]. Through repeated reinforcement, these safety behaviors gradually become automatically triggered by cues, forming habitual behaviors that are resistant to extinction [41,42].* *Therefore, we hypothesized that OCD patients would exhibit enhanced Pavlovian response tendencies driven by salient cues, paired with an instrumental learning bias characterized by abnormal updating of action‑outcome contingencies. When increased Pavlovian biases conflict with task goals, they can undermine goal‑directed control, allowing automatic responses to dominate and become resistant to extinction.” (****Pages 4–5 line 71–82****)*

At the same time, we agree that CSTC‑related findings remain relevant as part of the broader neurobiological literature. Therefore, we moved the CSTC‑related discussion to the Discussion section, where it is now used more cautiously to contextualize the behavioral modeling results rather than to motivate the main hypotheses directly. In the revised Discussion, we note that the heightened Pavlovian bias observed in OCD is consistent with previous evidence of ventral striatal hyperactivity and altered limbic‑network coupling, which may contribute to heightened sensitivity to motivationally salient cues. We also discuss the lower learning rate in relation to impaired flexible goal‑directed control, which has been linked in prior work to reduced activation in the ventromedial prefrontal cortex, orbitofrontal cortex, and caudate nucleus.

The revised Discussion now reads as follows:

*“This interpretation is consistent with prior evidence of ventral striatal hyperactivity [69,70] and hyper‑coupling within limbic networks [71] in OCD, which may provide a neural basis for heightened sensitivity to motivationally salient cues. Furthermore, the lower learning rate suggest that OCD patients can successfully learn the task rules, but they are still susceptible to interference when adjusting their choices based on feedback. This result reflects impaired flexible goal‑directed control [72–74], which is closely linked to reduced activation within the ventromedial prefrontal cortex, orbitofrontal cortex, and caudate nucleus [75–77].” (****Page 32–33 line 658–666****)*

In summary, we have revised the manuscript accordingly to ensure that CSTC circuitry is discussed as supporting background literature in the Discussion, rather than as a neural mechanism directly tested in the present study. This revision helps avoid the potential misunderstanding that CSTC abnormalities were directly examined in our EEG analyses.

(3) Regarding the behavioral analysis, by reading SI S4, it seems the analyses were quite thoroughly done, but it would be helpful to include key information in the text. By reading the Results (from Line 140), it seems that a few separate tests were used, but actually all analyses were linear mixed effect models. It would be helpful to briefly include these details (e.g., Linear mixed effect models with 2 within‑subject predictors (action, valence)). This would make sure that readers will not misunderstand the analyses.

⚫ **Reply:**

We agree that explicitly integrating brief methodological descriptions and model specifications within the Results section substantially elevates text transparency and prevents readers from misunderstanding the analytical pipeline as a series of isolated, ad‑hoc statistical tests. Instead of clustering statistical parameters at the beginning of sections, we adopted a stepwise narrative approach, embedding the exact analytical purpose, dependent variables, and model designs right before presenting each specific finding. Importantly, we have extended this rigorous reporting principle across the entire Results section, including the behavioral, time*‑*frequency (theta power), and inter‑site phase clustering (ISPS) network results.

The detailed revisions are below:

- In the behavioral results section

*“To examine whether participants learned the instrumental actions and to determine whether motivational valence biased behavioral activation, we first analyzed Go response probability. In these logistic mixed‑effects models, Required Action (Go vs. NoGo) and Valence (“win” vs. “avoid” cues) were entered as within‑subject predictors, and these models were fitted separately within the HC and OCD groups.” (****Pages 7–8 line 140–145****)*

*“To test whether these effects differed between groups, we then fitted a combined logistic mixed‑effects model in which Group (HC vs. OCD) was added as a between‑subject predictor, together with Required Action, Valence, and their interactions.” (****Page 8 line 151–154****)*

*“Furthermore, to determine whether reduced Go responding in OCD was accompanied by impaired performance when Go responses were required, we conducted an additional logistic mixed‑effects model restricted to Go cues. In this model, the dependent variable was Go‑cue accuracy, and Valence (“win” vs. “avoid” cues), Group (HC vs. OCD), and their interaction were entered as predictors.” (****Page 8 line 158–163****)*

*“Finally, to assess learning dynamics under motivational conflict, we extracted condition‑specific learning slopes from the trial‑by‑trial Go response probability model described above. These slopes captured the change in Go response probability over trials. For example, for NGW cues, in which the correct response was to withhold a Go response, a more negative slope would indicate faster acquisition of response inhibition (i.e., a rapid reduction in erroneous Go responses).” (****Page 9 line 165–171****)*

- In the Time‑Frequency (midfrontal theta power) results section

We added a direct declaration of the permutation test framework and the subsequent between‑group extraction strategy to ensure clarity:

*“More specifically, we performed a time‑based permutation test on cue‑locked, trial‑averaged midfrontal theta power in the 4–8 Hz band to identify time windows showing significant differences between motivationally incongruent and congruent cues. This analysis was restricted to correct trials to minimize the potential contribution of error‑related theta activity.” (****Page 14–15 line 278–282****)*

*“To examine whether the magnitude of conflict‑related theta enhancement differed between groups, we extracted the theta contrast (incongruent–congruent) from the significant cue‑locked time window and compared this contrast between HC and OCD participants.” (****Page 17 line 327–330****)*

- In the ISPS results section

We explicitly introduced the repeated‑measures ANOVA structure used for the single‑trial extracted phase‑synchrony metrics to maintain a unified reporting standard:

*“To examine conflict‑related network coordination, we extracted theta‑band ISPS values from correct trials within the significant cue‑locked theta window and used repeated‑measures ANOVAs with Valence (“win” vs. “avoid” cues) and Required Action (Go vs. NoGo) as within‑subject factors to test whether MFC‑lPFC and MFC‑motor phase synchrony increased for motivationally incongruent relative to congruent cues.” (****Page 21 line 418–423****)*

Together, these revisions make the analytical framework clearer in the main text while retaining the full model specifications in Supporting information S4 Text.

(4) Regarding ERP, though it is acknowledged that the N2 component has mixed findings, it would still be helpful to conduct the analysis which can be a valuable addition to the literature (it can be included in the SI).

⚫ **Reply:**

Following this recommendation, we conducted an additional exploratory analysis of cue‑locked ERP activity focusing on the midfrontal N2 component and report the full results in the **Supporting information S9 Text**.

Specifically, using the same preprocessed EEG dataset as in the main time‑frequency analyses, we extracted mean N2 amplitudes from a 288−350ms post‑cue time window over a midfrontal region of interest corresponding to Fz/Cz/FCz. Incongruent trials included the NoGo‑to‑Win and Go‑to‑Avoid conditions, in which Pavlovian response tendencies conflicted with the required instrumental response. Congruent trials included the Go‑to‑Win and NoGo‑to‑Avoid conditions. We then conducted paired‑samples t‑tests separately within the HC and OCD groups to examine N2 congruency effects, and an independent‑samples t‑test to compare HC and OCD participants under the incongruent condition.

The results showed that incongruent trials elicited significantly more negative N2 amplitudes than congruent trials in both groups. This effect was significant in the HC group (*t*(36) = −5.55, *p* < .001, Cohen’s *d_z_* = −0.91), and also in the OCD group (*t*(35) = −5.16, *p* < .001, Cohen’s *d_z_* = −0.86). However, the between‑group comparison of N2 amplitudes during incongruent trials was not significant (*t*(71) = −1.00, *p* = .318, Cohen’s *d* = −0.23).

These supplementary ERP findings indicate that both HC and OCD participants showed reliable N2 congruency effects, whereas the N2 response to incongruent trials did not significantly differ between groups. This pattern is in line with the cue‑locked midfrontal theta power results reported in the main text, where both groups showed conflict‑sensitive midfrontal neural responses during cue processing. Given that the central aim of the present study was to examine dynamic time‑frequency mechanisms of motivational conflict processing, we report this N2 analysis in the Supporting information as an additional ERP‑based characterization rather than as part of the main results. In the revised Supporting information, we have added this analysis as S9 Text, Cue‑locked midfrontal N2 amplitude, together with the corresponding ERP waveform, scalp topography, and group‑comparison figures.

(5) For model M4(a‑d), it is a good idea to see whether midfrontal theta power can further explain the data. For M4b, and the key equation (7), I wonder why it is a minus sign in (1‑beta*theta)*Q? The main text did not have the equation, so it is not straightforward to interpret a negative beta parameter with increased Q value. Also, Q is not a parameter (Line 327) but a model internal variable (or decision variable). And for M4c, I am not sure if it is needed. It is not included in the M3 model family, and it is not winning anyway. I think it can add more confusion than value. Last, it would be helpful to more explicitly map Equations 6*–*9 to the models M4a*–*d. Last, it seems counterintuitive to have M4 models in the methods but M5 models in the SI.

⚫ **Reply:**

We agree that the original description of computational models could be made more explicit. To address the reviewer's concerns, we systematically revised the main Methods section, the Results section, and Supporting information S5 Text. These updates improved our parameterization sign conventions, rectified loose terminology regarding Q values, clarified the role of M4c, established a direct mapping between equations and model labels, and harmonized the structural placement of the M5 family. Our specific point‑by‑point responses and corresponding revisions are detailed below:

- For M4b, and the key equation (7), I wonder why it is a minus sign in (1‑beta*theta)*Q? The main text did not have the equation, so it is not straightforward to interpret a negative beta parameter with increased Q value.

⚫ **Reply:**

We agree with the reviewer that greater transparency regarding our parameter sign conventions is needed. To address this, we have explicitly inserted the formal piecewise equations of the winning M4b model into the main text and added a dedicated explanatory narrative.

In Model M4b (Equation 7), trial‑by‑trial midfrontal theta power (θ_t_) acts as a multiplicative gain on the contribution of model‑derived instrumental Q values to action weights, implemented via a subtraction formulation: (1 – β×θ_t_) Q. Crucially, this minus sign is a deliberate parameterization strategy designed to achieve functional and semantic alignment of the β parameter across the entire model family (M4a–c). We calibrated the signs so that a negative parameter estimate (β < 0) uniformly indexes adaptive, goal‑directed conflict regulation. We updated the manuscript to make this global alignment explicit to the reader.

In M4a (Equation 6), β < 0 reduces the maladaptive Pavlovian bias term (π + β×θ_t_, becoming π ‑ β×θ_t_), signaling the adaptive suppression of Pavlovian bias. (**Page 52 line 1029*–*1030**)

In M4b (Equation 7), due to the minus sign within the multiplier, an empirical negative β mathematically cancels out the negative sign, yielding a positive scaling factor: (1 – (–β) ×θ_t_) = (1 + |β|×θ_t_). This effectively amplifies the weighting of the instrumental system as conflict‑related theta power increases, indicating an adaptive increase in instrumental contribution. (**Pages 52*–*53 line 1032*–*1037**)

- Also Q is not a parameter (Line 327) but a model internal variable (or decision variable).

⚫ **Reply:**

We thank the reviewer for pointing this out. We agree that Q should not be described as a model parameter, because it is a model‑derived internal decision variable that tracks learned instrumental action values. We have therefore revised the relevant wording in the main text and checked the manuscript to ensure that Q is described accurately. Specifically, the original wording around M4b has been revised to clarify that theta power scaled the contribution of model‑derived Q values to choice, rather than scaling a “Q parameter.” The revised text now states:

*“In both groups, M4b, which used learned Q value as internal variable tracking instrumental action values and allowed theta power to scale their contribution to choice, provided the strongest model evidence.” (****Pages 19–20 line 385–388****)*

We also revised related wording throughout the manuscript to refer to “model*‑*derived Q values”, “instrumental action values” or “instrumental contribution during choice”, rather than treating Q as a free parameter.

- And for M4c, I am not sure if it is needed. It is not included in the M3 model family, and it is not winning anyway. I think it can add more confusion than value.

⚫ **Reply:**

We acknowledge that the description of Model M4c in our original manuscript was brief, which understandably led to the reviewer’s lack of clarity regarding its motivation, especially given that the trade‑off parameter τ was not part of the baseline M3 behavioral family and M4c did not emerge as the winning architecture. To reduce confusion, we have revised the text to explicitly state that M4c was included as an exploratory arbitration model rather than as a core extension of the M3 behavioral family. Its purpose was to test an alternative possibility: that midfrontal theta might shift the relative balance between Pavlovian and instrumental control, rather than selectively modulating Pavlovian response tendencies or instrumental contribution.

We have revised the relevant statements in the main text accordingly *(****Page 53 line 1039–1050****)*:

*“In M4c, we developed an exploratory arbitration model to test whether midfrontal theta power shifted the balance between Pavlovian and instrumental control, rather than selectively modulating either Pavlovian response tendencies or the instrumental contribution. This model was not intended as an extension of a parameter already selected in the M3 behavioral family; instead, it served as an alternative comparison model to evaluate whether theta acted through a more global Pavlovian‑instrumental trade‑off mechanism. Specifically, M4c used a Pavlovian‑instrumental trade‑off parameter τ, and β captured whether theta power shifted decision weighting toward Pavlovian or instrumental control on motivationally incongruent trials. In this model, a positive β indicates a shift toward stronger Pavlovian tendencies and weaker instrumental contribution, whereas a negative β indicates a shift toward stronger instrumental contribution and weaker Pavlovian tendencies.”*

- Last, it would be helpful to more explicitly map Equations 6‑9 to the models M4a‑d.

⚫ **Reply:**

We have revised the main Methods section to explicitly map Equations 6–9 onto the corresponding M4 models. The revised text now states that Equations 6–9 correspond to M4a–M4d, respectively, and each equation is now introduced by a brief explanation of the model mechanism and the interpretation of β:

• Equation 6: M4a, theta modulation of Pavlovian response tendencies, is now described here:

*“In M4a, we tested whether midfrontal theta power modulated Pavlovian response tendencies. In this model, theta power scaled the Pavlovian bias (π) on incongruent trials. Given the form of the equation, a negative β value corresponds to theta‑related adaptive suppression of Pavlovian response tendencies during motivational conflict.” (****Page 52 line 1027–1030****)*

• Equation 7: M4b, theta modulation of instrumental contribution during choice is now described here:

*“In M4b, we tested whether midfrontal theta power modulated the contribution of learned instrumental action values to choice. In this model, theta power acted as a multiplicative gain on the model‑derived Q‑value term, such that the instrumental contribution was scaled by (1 − β×θ_t_). Under this specification, a negative β indicates that higher theta power increases the contribution of instrumental action values to choice.” (****Pages 52–53 line 1032–1037****)*

• Equation 8: M4c, exploratory arbitration model of Pavlovian‑instrumental balance, is described here:

*“In M4c, we developed an exploratory arbitration model to test whether midfrontal theta power shifted the balance between Pavlovian and instrumental control, rather than selectively modulating either Pavlovian response tendencies or the instrumental contribution. This model was not intended as an extension of a parameter already selected in the M3 behavioral family; instead, it served as an alternative comparison model to evaluate whether theta acted through a more global Pavlovian‑instrumental trade‑off mechanism. Specifically, M4c used a Pavlovian‑instrumental trade‑off parameter τ, and β captured whether theta power shifted decision weighting toward Pavlovian or instrumental control on motivationally incongruent trials. In this model, a positive β indicates a shift toward stronger Pavlovian tendencies and weaker instrumental contribution, whereas a negative β indicates a shift toward stronger instrumental contribution and weaker Pavlovian tendencies.” (****Page 53 line 1039–1050****)*

• Equation 9: M4d, theta modulation of instrumental learning bias is described here:

*“In M4d, we tested whether midfrontal theta power modulated instrumental learning bias. In this model, β scaled the biased learning‑rate terms for rewarded Go responses and punished NoGo responses, allowing them to move toward the unbiased learning rate ε_0_. Thus, a larger positive β indicates that theta power reduces the instrumental learning bias and makes updating more similar to the unbiased learning rate.” (****Page 54 line 1052–1057****)*

- Last, it seems counterintuitive to have M4 models in the methods but M5 models in the SI.

⚫ **Reply:**

We have moved the core description of the M5 model family into the main Methods section, immediately after the description of the M4 model family. In the revised text, we now clarify that the M5 models used the same mathematical architecture, parameter constraints, and sign conventions as the corresponding M4 theta‑power models, but replaced local midfrontal theta power with single‑trial theta*‑*band intersite phase synchrony estimates as the modulation signal. We also explicitly mapped each M5 model to its corresponding equation in Supporting information S5 Text.

The revised text now states:

*“Finally, to assess whether theta‑related modulation of motivational biases depended on long‑range network coordination, we extended the same modeling framework to an intersite phase synchrony modulation family (M5a–M5d). In these models, single‑trial theta‑band phase synchrony estimates replaced local midfrontal theta power (θ_t_) as the modulation signal, while the mathematical architecture, parameter constraints, and sign conventions were inherited from the corresponding M4 power‑based models. Specifically, we tested whether trial‑by‑trial MFC‑lPFC theta phase synchrony (ISPS_lPFC,t_; M5a, S5 Text, Equation 11) and MFC‑motor theta phase synchrony (ISPS_motor‑contra,t_; M5b, S5 Text, Equation 12) modulated the instrumental contribution during choice. We further tested whether MFC‑lPFC theta phase synchrony (ISPS_lPFC,t_; M5c, S5 Text, Equation 13) and MFC‑motor theta phase synchrony (ISPS_motor‑contra,t_; M5d, S5 Text, Equation 14) modulated Pavlovian bias. Detailed equations, parameter constraints, and implementation details for the M5 model family are provided in S5 Text.”* ***(Pages* 54−55 *line 1059–1072)***

In addition, we retained the full mathematical equations, parameter constraints, and implementation details in Supporting information S5 to avoid overloading the main Methods section. These revisions make the presentation of the M4 and M5 model families more consistent and allow readers to understand the synchrony‑based models directly from the main text while still being able to consult the Supporting information for full technical details.

(6) I feel the narrative around M5 models somewhat not straightforward. It is unnatural to first introduce M5c‑d and then M5a‑b. It would be more helpful to have a more streamlined approach here.

⚫ **Reply:**

We have reorganized the M5 model family throughout the manuscript, including the Methods section (**Pages 54−55** **line 1059–1072**), Results section (**Pages 24−26** **line 473–531**), Table 1(**Page 11 line 207–208**), and Figures 5(**Pages 22–23** **line 449–465**) and 6 (**Pages 28−29** **line 574–583**). In the revised version, M5a and M5b test whether MFC‑lPFC and MFC‑motor theta phase synchrony modulate the instrumental contribution during choice, whereas M5c and M5d test whether the same synchrony measures modulate Pavlovian bias.

Accordingly, the revised model ordering is now:

- M5a: MFC‑lPFC synchrony modulation of instrumental contribution;
- M5b: MFC‑motor synchrony modulation of instrumental contribution;
- M5c: MFC‑lPFC synchrony modulation of Pavlovian bias;
- M5d: MFC‑motor synchrony modulation of Pavlovian bias.

These revisions provide a more linear and coherent presentation of the M5 model family and should make it easier for readers to follow how the synchrony‑based models extend the M4 theta‑power models.

(7) More critically, the inclusion of effects of phase synchrony in models seems to be separate for Pavlovian bias (pi) and value (Q), but Fig 6 A**–**B is plotting them in the same space. I find this approach somewhat problematic, as the two parameters being plotted (Eg. F6A**–**B) do not belong to the same model. I think the rationale makes sense, but if the two parameters are estimated in two models, they may capture overlapping variances than if the model is estimated in one go (ie, testing the synchrony effect both for pi and Q in the same model).

⚫ **Reply:**

We thank the reviewer for this important and constructive comment. We agree that plotting subject‑level β parameters estimated from separate synchrony models in a common regulatory‑orientation space raises a potential concern, because separately estimated effects on instrumental contribution and Pavlovian bias may capture overlapping variance. To address this issue, we have clarified the logic of our model family and conducted additional joint‑model analyses in which the two synchrony*‑*related modulation effects were estimated simultaneously within the same model likelihood.

First, we would like to clarify the rationale underlying the original model construction. In the local midfrontal theta‑power model family, we tested four alternative mechanisms by which cue‑related midfrontal theta power could alter motivational choice. Specifically, M4a tested whether midfrontal theta power modulated Pavlovian response tendencies, M4b tested whether it modulated instrumental contribution, M4c tested whether it modulated the balance between Pavlovian and instrumental contributions, and M4d tested whether it modulated instrumental learning bias. Thus, the possibility of a Pavlovian‑instrumental trade‑off was already directly tested in M4c. This model parameterized the relative balance between Pavlovian value and instrumental contribution through a trade‑off parameter and allowed trial‑by‑trial midfrontal theta power to dynamically modulate this balance. However, M4c was not the winning model. Instead, M4b provided the best account of the data, suggesting that, at the level of local midfrontal theta power, motivational‑conflict processing was better explained by modulation of instrumental contribution than by a global Pavlovian‑instrumental trade‑off mechanism.

Based on this result, the subsequent phase‑synchrony model family was designed to examine whether inter‑regional theta phase synchrony between MFC and task*‑*relevant regions provided a network‑level mechanism for trial‑by‑trial modulation of motivational biases. Specifically, M5a and M5b tested whether MFC‑lPFC or MFC‑motor theta phase synchrony modulated instrumental contribution. In parallel, because parameter estimates from the baseline behavioral model indicated stronger Pavlovian bias in OCD patients, M5c and M5d tested whether MFC‑lPFC or MFC‑motor theta phase synchrony modulated Pavlovian bias. Thus, the original synchrony models were constructed to test two computationally distinct regulatory mechanisms: synchrony‑related modulation of instrumental contribution and synchrony‑related modulation of Pavlovian response tendencies.

Nevertheless, we agree with the reviewer that estimating these two synchrony effects in separate models does not rule out the possibility that the corresponding β parameters may capture overlapping variance. To directly address this concern, we conducted additional joint‑model analyses in which synchrony‑related modulation of instrumental contribution and Pavlovian bias was estimated simultaneously within the same model likelihood. For healthy controls, the joint model M6 included MFC‑lPFC phase‑synchrony modulation of instrumental contribution and MFC‑motor phase*‑*synchrony modulation of Pavlovian bias. For OCD patients, the joint model M7 included MFC‑motor phase‑synchrony modulation of both instrumental contribution and Pavlovian bias. We have also added a clarification to the revised main text to explain this additional joint‑model analysis, and the **Supporting information S14 Text** provide a detailed description of these analyses.

*“To address the possibility that separately estimated synchrony effects on instrumental weighting and Pavlovian bias might capture overlapping variance, we further fitted joint theta phase‑synchrony models for both groups, in which the relevant β parameters for instrumental contribution and Pavlovian bias were estimated simultaneously within the same model likelihood. The jointly estimated β parameters were highly consistent with those obtained from the original single‑effect models. Detailed results of these analyses are provided in S14 Text.”* ***(page 28 line 561–568)***

The supplementary analyses provided three converging checks. First, subject‑level β estimates from the original single‑effect synchrony models were strongly correlated with the corresponding estimates from the joint models across all four comparisons (r = 0.80–0.99, all p < 0.001). This indicates that adding the second synchrony‑modulation term into the same likelihood did not substantially distort, attenuate, or reorder the participant‑level estimates. Second, when the β parameters estimated within the same model likelihood were projected into the same two‑dimensional regulatory‑orientation space, the group‑specific spatial pattern was largely preserved. Healthy controls continued to show a complementary regulatory pattern involving stronger instrumental contribution and stronger suppression of Pavlovian bias, whereas OCD patients continued to show a more motor‑dominant and heterogeneous regulatory‑orientation pattern. Third, the clinical association between regulatory orientation and obsessive‑thought severity was also preserved when Φ_reg_ was derived from the jointly estimated synchrony‑modulation parameters, remaining significant after controlling for anxiety, depression, and trait impulsivity.

Together, these findings suggest that the original regulatory‑orientation visualization was unlikely to be driven solely by plotting parameters obtained from separate models. Rather, the same individual‑difference structure remained evident when synchrony‑related modulation of instrumental contribution and Pavlovian bias was estimated simultaneously within a single model likelihood. We therefore interpret these new analyses as supplementary sensitivity checks that support the robustness of the original visualization, while complementing rather than replacing the main model*‑*comparison results.

(8) Regardless, I quite like the approach of computing the orientation angle (S6), and I think this should be included (and visualized) in the main text. In the end, OCD patients are being tested, it would be very valuable to show the clinically meaningful results in a more explicit manner. Relatedly, just for the winning models without the EEG data, I wonder if the Pavlovian Bias and learning rates (the two that show differences between OCD and HC) are also correlated with OCD symptoms?

⚫ **Reply:**

In the revised manuscript, we have made the rationale and interpretation of this analysis more explicit in the main Results section. Specifically, we now introduce the regulatory orientation angle (Φ_reg_) before reporting the symptom‑related analysis and clarify that Φ_reg_ was treated as a continuous measure of regulatory orientation rather than as a categorical subgroup label. The revised text reads:

*“Importantly, Φ_reg_ was treated as a continuous measure of regulatory orientation rather than as a categorical subgroup label.” (****Page 30*** ***line 609–610****)*

Given space constraints and because this analysis was exploratory and supplementary to the primary model‑comparison results, we have retained the detailed derivation of Φ_reg_ in the Supporting information. We believe this maintains the focus of the main text while making the clinical motivation of the analysis clearer to readers.

In response to the reviewer’s additional question, we further examined whether individual differences in the two behavioral model parameters that differed between OCD and HC (learning rate and Pavlovian bias) were associated with clinical symptom severity within the OCD group. We conducted partial Spearman correlations among OCD participants between each parameter and Y‑BOCS scores (obsession subscale, compulsion subscale, and total score), controlling for BDI and BAI scores.

The results showed that learning rate tended to be negatively associated with Y‑BOCS scores, whereas Pavlovian bias tended to be positively associated with Y‑BOCS scores. However, none of these associations reached statistical significance. Therefore, these results should be interpreted cautiously and were not emphasized as main findings. The revised Figure is shown below:

**Fig 1.** Partial correlations between model parameters and Y‑BOCS scores in the OCD group. **(A)** Partial correlations of learning rate with Y‑BOCS total and subscale scores. **(B)** Partial correlations of Pavlovian bias with Y‑BOCS total and subscale scores. Partial Spearman correlations were computed after controlling for BDI and BAI scores. Scatter plots display the raw parameter and symptom scores for visualization, whereas the reported correlation coefficients and p‑values reflect the partial correlations. Blue lines indicate linear fits to the raw data, and shaded areas represent 95% confidence intervals.

(9) As learning rate is also a parameter that differed between OCD and HC, I am curious whether any EEG characteristics can also be examined?

⚫ **Reply:**

We performed an additional analysis of feedback‑locked midfrontal theta power (4–8Hz), given its established relevance to reinforcement learning and feedback processing. Using the same midfrontal electrode cluster and theta frequency range as in the cue‑locked analysis, we first identified feedback‑related theta time windows by contrasting non‑preferred with preferred outcomes separately in the HC and OCD groups. We then extracted theta power within these windows and examined whether feedback‑locked theta activity was related to instrumental learning bias. The full methodological details, statistical results, and corresponding figures have now been added to the **Supporting information S11 Text**.

First, feedback‑locked midfrontal theta power was primarily sensitive to feedback outcome: in both HC and OCD groups, non‑preferred outcomes, especially punishment, elicited stronger theta activity than preferred outcomes. Second, feedback‑locked theta activity did not significantly distinguish bias‑consistent feedback conditions, such as rewarded Go and punished NoGo, from bias‑inconsistent conditions, such as rewarded NoGo and punished Go. It also did not significantly differentiate Go‑related feedback from NoGo‑related feedback, which indexed relative enhanced versus reduced learning. Third, direct group‑level comparisons did not reveal significant differences between HC and OCD groups in feedback‑locked theta power.

We report these findings in the Supplementary Material rather than the main text. This allows us to fully address the reviewer’s suggestion while preserving the main focus of the manuscript, which centers on cue‑locked neural processes related to conflict monitoring, action selection, and the modulation of instrumental action values.

**2. Minor:**

(1) It would be helpful to more explicitly state the modeling is done under the hierarchical Bayesian framework in the main text. It'd also be beneficial to reference modeling practice papers on the workflow (e.g., Palminteri et al., 2017; Zhang et al., 2020)

• Palminteri, S., Wyart, V., & Koechlin, E. (2017). The importance of falsification in computational cognitive modeling. Trends in cognitive sciences, 21(6), 425‑433.

• Zhang, L., Lengersdorff, L., Mikus, N., Gläscher, J., & Lamm, C. (2020). Using reinforcement learning models in social neuroscience: frameworks, pitfalls and suggestions of best practices. Social Cognitive and Affective Neuroscience, 15(6), 695‑707.

⚫ **Reply:**

We agree that the hierarchical Bayesian nature of the computational modeling should be stated more explicitly in the main text. Accordingly, we have revised the Computational Modeling section to clarify that trial‑by‑trial choices in the motivational Go/NoGo learning task were modeled within a hierarchical Bayesian reinforcement‑learning framework.

In the revised text, we now explicitly state that individual‑level parameters characterizing learning, choice stochasticity, and motivational biases were estimated while being constrained by group‑level population distributions. This hierarchical structure allowed partial pooling across participants, thereby improving the stability of subject‑level parameter estimates while preserving inter‑individual variability.

We revised the Computational Modeling section as follows:

*“In this framework, nested reinforcement‑learning models were used to formally test whether Pavlovian response biases and instrumental learning biases contributed to asymmetric effects of valence on behavioral activation. Individual‑level parameters characterizing learning, choice stochasticity, and motivational biases were estimated while being constrained by group‑level population distributions. This hierarchical structure allowed partial pooling across participants, thereby improving the stability of subject‑level parameter estimates while preserving inter‑individual variability. Consistent with recommended practices in computational cognitive modeling [129,130], we specified theory‑driven candidate models, compared competing model families, inspected convergence diagnostics, and evaluated model adequacy using posterior predictive simulations. Detailed descriptions of the computational models and modeling procedures are provided in S5 Text.”* ***(Page 50* line 978–990*)***

Following the reviewer’s suggestion, we have also added references to computational modeling practice papers, including Palminteri et al. (2017) and Zhang et al. (2020). We further clarified that our modeling workflow involved theory‑driven candidate model specification, comparison of competing model families, inspection of convergence diagnostics, and evaluation of model adequacy using posterior predictive simulations. We have also updated the reference list to include these two important modeling practice papers. We believe that these revisions have improved the clarity of our modeling framework and strengthened the methodological rigor of the manuscript.

(2) It would be helpful to cite some EEG work on the similar task (e.g., Algermissen et al., 2024)

• Algermissen, J., Swart, J. C., Scheeringa, R., Cools, R., & den Ouden, H. E. (2024). Prefrontal signals precede striatal signals for biased credit assignment in motivational learning biases. Nat Commun, 15(1), 19.

⚫ **Reply:**

Algermissen et al. (2024) addressed the important question of how motivational learning bias arise during feedback‑based credit assignment. Specifically, using a similar motivational Go/NoGo task combined with simultaneous EEG‑fMRI, they showed that motivational biases are not limited to biased response selection, but also involve biased learning, such that rewards are preferentially attributed to active responses whereas punishments are less readily attributed to withheld actions. This work further showed that biased prediction‑error signals emerged in prefrontal/cingulate regions before striatal regions, highlighting the neural mechanisms underlying biased credit assignment during motivational learning.

We incorporated this citation in the section discussing the model‑based behavioral results. This placement was chosen because the primary focus of Algermissen et al (2024) is the emergence of learning‑stage bias, whereas the primary focus of the present study is how motivational biases are regulated during the choice stage in OCD. Nevertheless, our model‑based behavioral results are consistent with their central behavioral conclusion: motivational biases include dissociable Pavlovian response bias and instrumental learning bias. Therefore, this study provides relevant support for our model‑based behavioral interpretation.

We revised the Discussion section as follows:

*“Behavioral modeling confirmed that motivational biases include dissociable Pavlovian and instrumental learning biases, corroborating previous research [49,65–67]”* ***(Page 32* line 650–652***)*

67. *Algermissen J, Swart JC, Scheeringa R, Cools R, den Ouden HEM. Prefrontal signals precede striatal signals for biased credit assignment in motivational learning biases. Nat Commun. 2024;15(1):19.* [*https://doi.org/10.1038/s41467-023-44632-x*](https://doi.org/10.1038/s41467-023-44632-x) *PMID: 38168089*

**⮚ Reviewer #2**

**1. Major:**

(1) Causal claims vs correlational design

The manuscript frequently makes mechanistic and quasi‑causal claims (e.g., that impaired MFC‑motor synchrony drives maladaptive behaviour and constitutes a treatment target). However, all analyses are correlational.

‑ The data show associations between ISPS and behaviour/model parameters—not causation. The wording “drives maladaptive motivational behaviour” (including in the title) is too strong.

Recommendation:

- Temper claims throughout (title, abstract, discussion). Consider framing as:
- “is associated with”
- “may contribute to”
- “is consistent with a mechanism involving…”

**⚫ Reply:**

We agree that scientific rigor requires highly cautious framing to ensure that our findings are not misinterpreted as making definitive causal claims. Following this excellent recommendation, we have thoroughly audited the entire manuscript to refine our terminology and adopt a more measured tone throughout the Title, Abstract, and Discussion sections. Specifically, we have systematically replaced verbs that carry deterministic connotations with more precise and descriptive alternatives that accurately align with our analytical framework.

First, we modified the Title of the manuscript to ensure a more descriptive and rigorous presentation of our core finding. The updated title now states that impaired midfrontal‑motor theta phase synchronization “characterizes” maladaptive motivational behavior in obsessive‑compulsive disorder rather than asserting that it “drives” this behavior.

Second, within the description of our computational modeling families and research objectives in both the Methods and Results sections, we have carefully replaced the phrase “contributed to” with “was/were associated with” (**Page12 line 219; Page 24 line 471; Page 24 line 483; Page 25 line 507; Page 31 line 636**). This adjustment ensures that our hypothesis testing and results are framed in terms of systematic associations between trial‑by‑trial network synchronization and motivational bias modulation, thereby avoiding premature mechanistic inferences.

Third, in the final paragraph of the Discussion where we touch upon the clinical implications of our findings, we have restructured our conclusion to present a more balanced perspective. Instead of presenting the midfrontal‑motor pathway as an absolute and definitive treatment target, the revised text now states that within the network perspective, this synchrony represents a promising candidate pathway that may contribute to the future development of circuit‑based interventions. **(Page 40 line 820–821)**

We believe that these widespread refinements across the manuscript successfully elevate the precision of our language and keep our conceptual inferences strictly within the bounds of scientific caution as suggested.

(2) Interpretation of delayed theta = impaired control

The key claim is that theta timing overlap with response reflects delayed control in OCD.

Concerns:

- Reaction time differences and variability may confound this interpretation.
- Overlap with response does not necessarily imply functional inefficacy. It could reflect prolonged processing, motor contamination, or different strategy.
- No direct test linking trial‑level theta timing → behavioural success is presented.

Recommendation:

- Provide trial‑level analyses linking pre‑response theta vs performance.
- Control explicitly for RT variability.
- Consider alternative interpretations.

**⚫ Reply:**

Following the reviewer’s suggestion, we further examined whether the observed pre‑response theta effect could be explained by RT differences, RT variability, or practice‑related effects. We also conducted additional analyses to explore the relationship between pre‑response theta activity and behavioral performance. These results have been reported in the **Supporting information S10 Text**, and we have revised the interpretation in the results (**Pages 18−19** **line 346–368**) and discussion (**Pages 36–37** **line 741–749**) accordingly.

Specifically, we added a new supplementary section (S10 Text), including single*‑*trial LMMs controlling for trial‑level RT, subject‑level RT variability, and trial order, as well as single‑trial GLMMs linking pre‑response theta to behavioral accuracy. We also revised the Results and Discussion to avoid equating theta‑response overlap with functional inefficacy and to explicitly consider alternative interpretations, including prolonged processing, motor‑related activity, and different response strategies.

First, to examine whether the conflict‑related pre‑response theta effect differed between HC and OCD participants after accounting for RT‑related factors and practice effects, we conducted additional single‑trial linear mixed‑effects models (LMMs). These models controlled for trial‑level RT, subject‑level RT variability, and trial order. The results showed that trial‑level RT and trial order were significantly associated with pre‑response theta power, indicating that response speed and task progression contributed to trial‑level theta variability. Importantly, however, after controlling for these factors, HC participants still showed significantly stronger pre‑response theta power for Go‑to‑Avoid than Go‑to‑Win trials (estimated difference = 0.16, 95% CI [0.039, 0.279], *t* = 2.60, *p* = 0.009). In contrast, the Go‑to‑Avoid versus Go‑to‑Win difference remained non‑significant in OCD patients (estimated difference = −0.03, 95% CI [−0.158, 0.101], *t* = −0.44, *p* = 0.663). These results indicate that the pre*‑*response conflict‑related theta enhancement observed in HC participants was robust after controlling for RT‑related factors and trial‑order effects, whereas OCD patients still failed to show a reliable conflict‑specific pre‑response theta enhancement.

Second, to directly examine whether pre‑response theta was related to behavioral performance, we conducted single‑trial generalized linear mixed‑effects models (GLMMs) predicting trial‑level accuracy. Pre‑response theta significantly and positively predicted accuracy (β = 0.33, 95% CI [0.272, 0.380], *z* = 11.86, *p* < 0.001, OR = 1.39, 95% CI [1.31, 1.46]), indicating that higher pre‑response theta was associated with a greater probability of correct responding. This positive theta–accuracy relationship was present in both groups, but it was significantly weaker in OCD patients than in HC participants (estimated difference = 0.33, 95% CI [0.222, 0.436], *z* = 6.00, *p* < 0.001, OR = 1.39, 95% CI [1.25, 1.55]). Thus, pre‑response theta activity remained behaviorally relevant in OCD patients, but unlike in HC participants, it was not selectively enhanced by motivational conflict before response execution. Detailed trial‑by‑trial analyses supporting these conclusions are now provided in **Supporting information S10 Text**.

Third, we revised the interpretation of the theta timing effect in the manuscript (**Page 36–37** **line 743–749**). We no longer interpret theta‑response overlap as direct evidence of functional inefficacy. Instead, we now state that single‑trial analyses showed that pre‑response theta significantly predicted accuracy, but OCD patients did not show the conflict‑specific pre‑response theta enhancement observed in HC participants. This pattern suggests that OCD patients retained behaviorally relevant pre‑response theta activity, but this signal was less flexibly scaled to conflict demands before action initiation, consistent with previous accounts of weakened proactive control before responding in OCD. Thus, differing from the strategy observed in HC participants, under motivational conflict, control‑related theta activity in OCD may be less effectively organized before the response and instead become more evident closer to motor execution.

We also revised the summary interpretation in the Discussion section to take into account the reviewer’s alternative explanations **(Page 40 line 808–811)**. Specifically, we now describe the conflict‑related theta response in OCD as appearing more prolonged and extending into the response window. This temporal extension suggests that the expression of conflict modulation in OCD was shifted closer to the motor stage, rather than clearly organized before response execution. We acknowledge that this pattern may reflect prolonged processing, motor‑related overlap, or a different response‑proximal control strategy, and we therefore interpret it more cautiously as altered temporal organization of control‑related theta activity rather than as direct proof of impaired control efficacy.

(3) Interpretation of heterogeneity

The identification of two regulatory subtypes in OCD is interesting but currently underdeveloped.

Concerns:

- Subgroups appear post hoc and descriptive.
- No clustering method or statistical validation is reported.
- The “two orientations” may reflect a continuous distribution.

Recommendation:

- Apply formal clustering or mixture modelling.
- Test whether subgroups are statistically separable.
- Clarify whether the angular metric (Φ_reg_) captures a continuum rather than discrete types.

**⚫ Reply:**

We agree that our original wording could be interpreted as implying discrete OCD regulatory subtypes or statistically separable subgroups, although no formal clustering or mixture modeling was performed. Given the current sample size, we do not consider the present data suitable for reliable subgroup identification using clustering or mixture‑modeling approaches. Our intention was instead to characterize continuous individual differences in regulatory orientation.

Importantly, the angular metric Φ_reg_ was designed to quantify regulatory orientation as a continuous individual‑differences measure rather than as a categorical subgroup label. We recognize, however, that our original wording, including terms such as “subgroups” “two distinct patterns” “phenotypes” may have unintentionally implied a discrete classification. To address this concern, we have revised the manuscript throughout to align the interpretation with a continuous individual*‑*differences framework.

First, we revised the Results section introducing Figure 6 (**Pages 29−30 line 595–601**). We removed categorical language and now describe the quadrants in Figure 6B as descriptive heuristic regions that capture divergent regulatory tendencies within a continuous two‑dimensional regulatory‑orientation space, rather than as statistically defined clusters or discrete subtypes. We revised the Figure 6C legend to clarify that the schematic illustrates representative regulatory tendencies within a continuous orientation space, rather than statistically discrete clusters or categorical subtypes.

Second, we reframed the relevant Discussion paragraphs (**Pages 37−38 line 756–768**). We now state that OCD patients showed continuous individual variability in regulatory orientation, and that Φ_reg_ captured clinically meaningful variation in this space, as reflected by its significant association with obsessive‑thought severity.

Finally, we expanded the limitations and future directions section to explicitly acknowledge that formal clustering or mixture modeling was not performed because of sample size constraints (**Pages 38–39** **line 785–790**). We now state that future studies with larger and more clinically diverse samples should apply formal clustering or mixture‑modeling approaches to multidimensional symptom profiles to test whether OCD patients can be classified into symptom‑based or regulatory subgroups, and whether such potential groups differ in their modulation of Pavlovian bias and instrumental action‑value weighting during motivational conflict.

We believe these revisions provide a more cautious and statistically appropriate interpretation of the heterogeneity observed in OCD, while preserving the main conclusion that regulatory orientation varies continuously across patients and is associated with obsessive‑thought severity.

(4) Sample size and analytical complexity

While the sample size is reasonable, the study includes:

- hierarchical modelling
- time‑frequency analyses
- connectivity analyses
- individual‑difference modelling

This raises risk of overfitting and inflated false positives.

Recommendation:

- Discuss statistical power and robustness.
- Consider cross‑validation or split‑sample validation.

**⚫ Reply:**

We agree that the combination of hierarchical computational modelling, time*‑*frequency analyses, connectivity analyses, and individual‑difference modelling requires a clearer discussion of statistical power, robustness, and the potential risk of overfitting or inflated false positives. We have supplemented the manuscript with the effect size assumptions used in the G*Power sample size calculation and have additionally reported the effect sizes for the relevant statistical results.

First, we added a new subsection entitled “Sample size and power estimation” in the Statistical analyses section, with further details provided in the **Supporting information S4 Text**. In this section, we clarify that the primary analyses were hypothesis‑driven and focused on theoretically motivated contrasts, including the Valence × Required Action interaction and the congruent versus incongruent contrast. Because exact power estimation for mixed‑effects models, permutation‑based EEG analyses, and hierarchical computational models is not directly implemented in G*Power, we conducted approximate a priori power analyses using G*Power 3.1.9.7 based on the corresponding subject‑level repeated‑measures designs.

For the primary within‑subject motivational effect, the Valence × Required Action effect was approximated as a repeated‑measures design with four measurements. Assuming a medium effect size of *f* = 0.25, α = 0.05, 95% power, a correlation of 0.50 among repeated measures, and ε = 1, G*Power estimated a required total sample size of 36 participants. Thus, the sample size in each group was sufficient for detecting medium‑sized within‑subject effects, as the HC group included 37 participants and the OCD group included 36 participants. For group differences in this motivational effect, the Group × Valence × Required Action effect was approximated as a repeated*‑*measures within–between interaction with two groups and four repeated measurements. Under a more stringent criterion, assuming *f* = 0.25, α = 0.01, 99% power, a correlation of 0.50 among repeated measures, and ε = 1, G*Power estimated a required total sample size of 62 participants. The present sample included 73 participants in total, exceeding this requirement for detecting medium‑sized primary group‑by‑condition interaction effects.

We also clarified that statistical power was more limited for secondary individual‑difference analyses. These analyses evaluated the association between regulatory orientation and Y‑BOCS obsessive‑thought severity within the OCD group. A correlation‑based a priori power analysis indicated that, assuming a large correlation effect of ρ = 0.50, α = 0.05, 85% power, and a two‑tailed test, the required total sample size was 32 participants. Since the individual‑difference analysis included 36 OCD patients, the sample was adequately powered for detecting large correlation effects, but less well powered for small‑to‑moderate associations. Accordingly, these analyses were explicitly treated as secondary and interpreted cautiously. Their robustness was further assessed using partial correlations controlling for anxiety, depression, and trait impulsivity.

Second, we carefully considered the reviewer’s suggestion regarding cross*‑*validation or split‑sample validation. We agree that such procedures are valuable for evaluating generalizability, particularly in complex modelling frameworks. However, we concluded that a simple split‑sample validation was not well suited to the present clinical EEG dataset. Given the constraints of clinical EEG data, artifact rejection and trial exclusion procedures inevitably reduce the number of usable epochs. Partitioning the current sample would therefore substantially reduce the effective sample size within each group, especially for EEG connectivity metrics and individual‑difference analyses, and could lead to unstable parameter estimates. We therefore did not implement a simple split‑sample validation procedure. Instead, we addressed robustness using several complementary strategies. For the hierarchical computational modelling analyses, we compared models using WAIC and R². We now clarify in the Results that WAIC provides a complexity‑penalized estimate of model fit, reducing the likelihood that improved fit merely reflects over*‑*parameterization **(Page 10 line 195–198)**. Although WAIC is not equivalent to formal external validation, it provides an approximate out‑of‑sample predictive criterion and is appropriate for comparing hierarchical computational models within the present sample.

Third, we expanded the reporting of effect‑size and uncertainty indices across analyses **(Pages 8‒9; Page 12; Page 15; Pages 17‒18; Page 20; Pages 25‒26)**. For logistic mixed‑effects models, we now report odds ratios in addition to regression coefficients, χ² statistics, and *p* values. For repeated‑measures ANOVAs of theta phase synchrony, we report partial eta squared. For *t*‑tests, we report Cohen’s *d* or Cohen’s *d*_z_, as appropriate. For time‑frequency analyses, we report cluster‑corrected *p* values from permutation tests and supplement these with cluster‑window averaged contrasts, including *t* values and Cohen’s *d*_z_. For computational modelling analyses, we report WAIC, ΔWAIC, R², posterior distributions, 95% HDIs, posterior probabilities, and Bayes factors where appropriate.

Fourth, we clarified that the time‑frequency analyses used permutation‑based inference and that follow‑up tests were corrected for multiple comparisons (**Pages 14–15 line 278–282 Page 15 line 286–288**). We also clarified that the individual‑difference analysis was conducted as a continuous, secondary analysis rather than as a categorical subgroup analysis, and that the association between regulatory orientation and obsessive‑thought severity remained after controlling for anxiety, depression, and trait impulsivity (**Pages 37‒38 line 756–768**).

Finally, we added a limitation in the Discussion noting that formal split‑sample or external validation was not feasible in the present clinical sample because it would substantially reduce the effective sample size and statistical power. We therefore emphasize that future studies with larger, independent, and ideally multi‑site clinical cohorts will be important for validating the individual‑difference and computational modelling findings **(Page 39 line 793–800)**.

These revisions have been incorporated into the statistical analyses section in Supporting information, the Results section, and the Discussion section.

**2. Minor:**

(1) Clarify medication status of OCD participants (potential confound).

**⚫ Reply:**

we have now updated the Methods section (under "Participants") to state that all OCD patients were on stable psychotropic medication regimens. Furthermore, we have provided a detailed, comprehensive breakdown of the specific medication classes and participant counts in the **Supporting information S1 Text**.

1) Revision in the main text (Methods):

*“OCD patients were not medication‑free at the time of testing and remained on stable psychotropic medication during the experiment. Demographic characteristics and clinical scores appear in Table 2 whereas the detailed medication information and inclusion/exclusion criteria are provided in S1 Text.”* ***(Page 41 line 843–847)***

*“Patients in the OCD group remained on stable psychotropic medication during the experiment. Medications included selective serotonin reuptake inhibitors (SSRIs; n = 23), serotonin‑norepinephrine reuptake inhibitors (SNRIs; n = 1), atypical antipsychotics (n = 8), benzodiazepines (BZDs; n = 5), non‑benzodiazepine hypnotics/anxiolytics (n = 2), and antimanic agents (n = 1). Medication categories were not mutually exclusive, and some patients received more than one class of drug.”* ***(Supporting information S1 Text)***

We believe these additions provide full transparency regarding the pharmacological status of our clinical sample.

(2) Report comorbidities more explicitly.

**⚫ Reply:**

We applied highly stringent exclusion criteria during enrollment. We explicitly excluded any OCD patient with any current or lifetime psychiatric diagnosis other than OCD. Consequently, 100% of the OCD patients included in our final analysis had OCD as their primary diagnosis without any psychiatric comorbidities.

We have revised both the main text and S1 Text to state these criteria and the clinical purity of our sample more explicitly:

1) Revision in the main text (Methods):

*“OCD diagnoses were confirmed by licensed psychiatrists according to the Chinese version of DSM‑5. OCD patients with any current or lifetime psychiatric diagnosis other than OCD were excluded, including depressive disorders such as major depressive disorder, anxiety disorders such as generalized anxiety disorder, substance use disorders, bipolar disorder, post‑traumatic stress disorder, and personality disorders. Thus, all patients included in the final OCD sample had OCD as the primary diagnosis and did not meet criteria for current or lifetime psychiatric comorbidities.”* ***(Page 41 line 835–842)***

*“Exclusion criteria were: ... (2) any current or lifetime psychiatric diagnosis other than OCD, including depressive and anxiety disorders (e.g., major depressive disorder, generalized anxiety disorder), as well as substance use disorders, bipolar disorder, post‑traumatic stress disorder, or personality disorders.”* ***(Supporting information S1 Text)***

(3) Some terminology shifts between “instrumental learning bias” and “instrumental contribution”—define clearly and consistently.

**⚫ Reply:**

We agree that “instrumental learning bias”, “instrumental action values”, and “instrumental contribution” are central concepts in the present study and should be clearly distinguished and used consistently. We have therefore revised the manuscript to define these terms more explicitly in the Materials and Methods section and to ensure that each term is used according to its corresponding computational meaning.

Specifically, following the modelling logic of Swart et al. (2018):

**1) Instrumental learning bias** refers strictly to the κ parameter. This term applies to the experience‑dependent learning/update stage and captures outcome‑feedback‑dependent asymmetries in instrumental learning. Specifically, reward outcomes more strongly reinforce specific Go actions than NoGo responses, reflected in an increased learning rate for rewarded Go responses (𝜀_0_+ 𝜅). Conversely, punishment outcomes more strongly promote avoidance of preceding Go actions than NoGo responses, reflected in a reduced learning rate for punished NoGo responses (𝜀_0_− 𝜅).

**2) Instrumental action values** refer to the learned Q(a,s) values for candidate actions given a cue, that is, the internal latent variables that track the expected value of choosing a specific action in a given state.

**3)** **Instrumental contribution** refers to the choice‑stage contribution or weighting of these learned instrumental Q values during action selection. Thus, instrumental contribution is related to Q values.

Following this clarification, we carefully reviewed the manuscript and revised the relevant descriptions **(Page 19 line 376–378; Page 20 line 400; Page 24 line 477; Page 24 line 488; Page 29 line 580; Page 29 line 582; Page 29 line 584; Page 30 line 607–608; Page 32 line 644)** to ensure consistent terminology. In particular, M3b and M3c are now described as estimating the instrumental learning bias 𝜅, whereas M4d tests whether theta modulates this learning bias. In contrast, M4b and the corresponding theta phase‑synchrony models are described as testing whether theta power or theta phase synchrony modulates the instrumental contribution, that is, the weighting of instrumental action values during choice.

We also checked all occurrences of these terms throughout the manuscript to prevent conceptual overlap. Thus, when we refer to instrumental learning bias, we mean the 𝜅‑dependent learning asymmetry; when we refer to instrumental action values, we mean the learned Q values; and when we refer to instrumental contribution, we mean the extent to which those Q values influence choice at the decision stage. These revisions clarify the distinction between learning‑related mechanisms and choice‑stage mechanisms and should prevent ambiguity in the interpretation of our computational models.

(4) The introduction could better distinguish Pavlovian vs instrumental vs habit frameworks.

**⚫ Reply:**

We agree that explicitly distinguishing between Pavlovian, instrumental, and habit frameworks at the outset provides a much stronger theoretical foundation for the study.

We have therefore revised the introduction **(Pages 3‒4 line 57–67)** to define these three frameworks according to their underlying associative structures and computational characteristics. Specifically, we now clarify that **Pavlovian mechanisms** are based on stimulus‑outcome associations and refer to cue‑triggered global approach or avoidance response tendencies elicited by rewarding or aversive cues. In contrast, **instrumental mechanisms** support flexible goal‑directed control through action‑outcome learning, allowing actions to be selected and updated based on their consequences. We further clarify that, with repeated training and reinforcement, initially flexible action‑outcome control can become automatized and shift toward **stimulus–response habits**.

By explicitly distinguishing stimulus‑outcome, action‑outcome, and stimulus‑response frameworks, the revised Introduction now provides a clearer conceptual background for the present task. In particular, it clarifies how Pavlovian response tendencies can become maladaptive when they conflict with instrumental requirements, thereby increasing the need for cognitive control. These revisions also help position our computational models more precisely, as they focus on Pavlovian response bias and instrumental learning bias, while habit formation is discussed as a related but conceptually distinct framework for understanding automatized cue‑driven behavior.

**⮚ Reviewer #3**

**1. Major:**

(1) I suggest the authors describe the EEG preprocessing and extraction in more detail. For example, what were the parameters were used to remove artifacts using ICA (manual rejection or automatic), how many (or what proportion of) ICAs were removed, how many trials were retained/removed, how was the theta extraction window decided, how cue‑locked theta enhancement time was defined, etc. Ideally, authors report enough for someone with a similar data to be able to replicate the study.

**⚫ Reply:**

We agree that a clearer and more quantitative description of EEG preprocessing and feature extraction is important for improving the reproducibility of the study. We have therefore revised the EEG preprocessing **(Pages 46‒47 line 894–928)** and Time‑frequency **(Pages 48‒49 line 934–957)** decomposition subsections in the Methods section to provide more detailed information.

First, we expanded the description of the preprocessing pipeline. We now report the filtering parameters, including the 0.5Hz high‑pass filter and 50Hz low‑pass filter, the offline re‑referencing to the weighted average of the bilateral mastoids, the epoching window from 1.75s before cue onset to 1.5s after feedback offset, and the 200ms pre‑cue baseline correction.

Second, we clarified the artifact rejection and ICA procedures. We now state that trials containing electromyographic activity or other non‑neural artifacts unrelated to brain activity were manually rejected, whereas trials containing eye blinks were retained for subsequent ICA correction. We also clarify that ICA was performed using EEGLAB’s runica algorithm in extended mode, with dimensionality reduced to 64 components using principal component analysis. ICA components related to blinks, eye movements, muscle activity, or other artifacts clearly distinguishable from brain activity were manually identified and removed. Across participants, 4 to 17 components were removed, with an average of 9 components removed per participant. We also report that 2 to 11 channels were interpolated per participant, with an average of 5 interpolated channels.

Third, we added quantitative information about participant and trial rejection. Participants were excluded from EEG analyses if more than 35% of their trials were rejected because of excessive artifacts. Based on this criterion, seven participants were excluded, including three healthy controls and four patients with OCD. Among the retained participants, the proportion of rejected trials ranged from 0.47% to 33.96%, with a mean rejection rate of 6.97%. The final EEG analyses therefore included 37 healthy controls and 36 patients with OCD.

Fourth, we clarified how theta activity was extracted. We now specify that the analysis focused on theta‑band activity, defined as 4–8 Hz, over the midfrontal electrode cluster comprising Fz, FCz, and Cz. Cue‑locked theta enhancement was defined as the time window after cue onset in which midfrontal theta power was stronger for incongruent than congruent trials. To identify this window, we employed a time‑based permutation test with 500 permutations on cue‑locked, trial‑averaged midfrontal theta power, with congruency as the within‑subject factor. Go‑to‑Win and NoGo‑to‑Avoid cues were classified as congruent, whereas NoGo‑to‑Win and Go*‑*to*‑*Avoid cues were classified as incongruent. The significant time window identified by this test was then used as the theta extraction window. Theta power was then extracted from single‑trial data within the corresponding group‑specific window and used as a trial‑level EEG predictor in the computational models.

The relevant methodological details have been added to the revised Methods section and Supporting information S3 Text.

(2) Pages 13 and 14. Could the authors test for the theta enhancement and RT difference statistically? Also, the theta enhancement in Figure 4 seems to be over a narrower window in HC, but more spread out in OCD. Does that reflect more heterogeneity in OCD or do participants with OCD have elongated enhancement (rather than slower enhancement)? This is the finding that the authors base their argument on theta power not influencing choice, so a thorough statistical investigation would help.

**⚫ Reply:**

We implemented formal statistical tests for cue‑locked theta enhancement, between‑group contrasts in overall theta amplitude, and group‑level differences in mean reaction times. Furthermore, we added a rigorous OCD‑focused consistency analysis to determine whether the broader temporal envelope in OCD reflects a stable within‑group temporal pattern rather than a statistical artifact driven by outlying participants or unstable grand‑average blurring.

First, to statistically test cue‑locked theta enhancement, we used time‑based permutation tests on midfrontal theta power, with congruency, incongruent versus congruent, as the within‑subject factor **(Page 17 line 327–330)**. This analysis confirmed significant cue‑locked conflict‑related theta enhancement in both groups. In the HC group, incongruent trials elicited significantly stronger midfrontal theta power than congruent trials from 400 to 650ms after cue onset (cluster‑corrected *p* = 0.004). In the OCD group, a significant theta enhancement (incongruent–congruent) was observed from 424 to 800ms after cue onset (cluster‑corrected *p* = 0.002). To examine whether the magnitude of theta enhancement differed between groups, we extracted the theta contrast (incongruent–congruent) from the significant cue‑locked theta window and compared this contrast between HC and OCD participants. This between‑group comparison showed no significant difference in conflict‑related theta amplitude (*t*(71) = 1.19, *p* = 0.237, Cohen’s *d* = 0.28) **(Page 17 line 330–332)**. Thus, both groups showed significant cue‑locked theta enhancement during motivational conflict, but the magnitude of this enhancement did not significantly differ between groups.

Second, we statistically tested whether mean reaction time differed between HC and OCD participants. Mean reaction time did not differ significantly between groups (*t*(71) = −0.44, *p* = 0.661, Cohen’s *d* = −0.10) **(Page 17 line 340–341)**. This result indicates that the observed difference in the temporal profile of cue‑locked theta activity was unlikely to be explained simply by a significant group difference in overall response speed.

Finally, to determine whether the broader cue‑locked theta enhancement window observed in OCD reflected a reliable within‑group temporal pattern or was driven by a small subset of outlying participants, we conducted an OCD‑focused consistency and robustness analysis. This analysis specifically addressed whether the theta enhancement window (424–800ms) shown in the OCD group was consistently present across individual patients, rather than resulting from grand‑average blurring, extreme individual values, or unstable inter‑individual timing profiles.

For each OCD participant, we calculated the cue‑locked theta contrast (incongruent–congruent), averaged over the midfrontal electrodes and the 4–8 Hz frequency range. We then extracted the mean theta contrast within the OCD group‑level significant window (424–800ms), and tested whether this contrast was significantly greater than zero. To evaluate temporal persistence, the 424–800ms window was further divided into early, middle, and late subwindows. In addition, we quantified the proportion of participants showing positive theta contrast at each time point and performed leave‑one‑subject‑out analyses to test whether the broader temporal profile was driven by any single participant.

Within the full 424–800ms window, the theta contrast (incongruent–congruent) was significantly greater than zero (mean Δtheta = 0.333 ± 0.420, *t*(35) = 4.76, *p* < 0.001, Cohen’s *d_z_* = 0.79). Importantly, 86.11% OCD participants showed a positive theta contrast in this window, which significantly exceeded chance level (binomial *p* < .001). These findings indicated that the broader OCD theta window was not driven by a few participants with extreme positive values, but reflected a positive contrast present in the majority of patients.

**Fig 2. Within‑group consistency of cue‑locked theta enhancement in the OCD group.** Each row in the upper panel represents one OCD participant’s cue‑locked incongruent–congruent theta contrast, averaged over the midfrontal electrodes and the 4–8Hz frequency range. Participants were sorted according to their mean theta contrast within the OCD group‑level significant window (424–800ms), marked by dashed vertical lines. Warm colors indicate positive theta contrast, whereas cool colors indicate negative theta contrast. The lower panel shows the OCD group‑mean theta contrast with SEM. The predominance of positive contrasts within the window indicates that the broader OCD theta enhancement window was not driven by a small subset of participants.

The theta contrast remained significantly positive across all three subwindows: 424–550ms (*t*(35) = 3.53, *p* = 0.001, Cohen’s *d_z_* = 0.59); 550–674ms (*t*(35) = 4.75, *p* < .001, Cohen’s *d_z_* = 0.79); and 674–800ms (*t*(35) = 4.24, *p* < 0.001, Cohen’s *d_z_* = 0.71). Directional consistency was also observed across these subwindows, with 27 of 36 participants (75.00%) showing a positive contrast in the early window, 31 of 36 participants (86.11%) in the middle window, and 26 of 36 participants (72.22%) in the late window. Time‑resolved directional consistency analysis further showed that the proportion of OCD participants with a positive theta contrast remained above 50% throughout the 424–800ms window, with an average positive proportion of 76.91%, range: 61.11%–88.89%. Leave‑one‑subject‑out analyses confirmed the robustness of this effect, after sequentially removing each participant, the mean theta contrast remained positive and significant across all iterations, with leave‑one‑out mean contrasts ranging from 0.299 to 0.360 and all right‑tailed *ps* < 0.001.

Together, these results indicate that the broader cue‑locked theta enhancement window in OCD reflected a reliable and internally consistent within‑group temporal pattern, rather than a statistical artifact caused by outlying participants or unstable grand‑average blurring. Thus, OCD patients showed a robust conflict‑related midfrontal theta enhancement across the broader 424–800ms window. Importantly, this OCD‑focused analysis demonstrates the stability of the broader window within the OCD group, while avoiding the stronger claim that theta enhancement was statistically longer in OCD than in healthy controls.

**2. Minor:**

(1) I suggest the authors report the number of trials per condition that were used for the computational and EEG analyses. Particularly confirming that there were sufficient trials for the variables to stabilize would be helpful.

⚫ **Reply:**

In the revised manuscript, we added the trial counts for each experimental condition at the end of the EEG preprocessing subsection:

*“The task employed a 2×2 factorial design crossing Valence (“win” vs. “avoid” cues) and Required Action (Go vs. NoGo), yielding four experimental conditions. For the retained participants, the analyzed trial counts across the four conditions were Go‑to‑Win (85.40 ± 20.09), NoGo‑to‑Avoid (83.00 ± 17.41), NoGo‑to‑Win (70.22 ± 26.76), and Go‑to‑Avoid (74.10 ± 21.22). These trial counts indicate that EEG and computational variables were estimated from adequate numbers of observations across conditions.”* ***(Page 47 line 922–928)***

These additions allow readers to directly evaluate the number of observations contributing to each condition‑level EEG and computational estimate.

(2) The sample sizes are small as the authors acknowledge, so some results (such as correlations) should be interpreted carefully. The possibility of Type 1 error should be noted in the limitation.

⚫ **Reply:**

We agree that the modest sample size requires a cautious interpretation of some findings, particularly the exploratory correlational analyses. To improve the transparency and interpretability of the results, we supplemented the manuscript with additional effect‑size and uncertainty indices where appropriate, including odds ratios for logistic mixed‑effects models, partial eta squared for repeated‑measures ANOVAs, Cohen’s *d* or Cohen’s *d_z_* for *t*‑tests and cluster‑window averaged contrasts, and posterior uncertainty indices for computational modelling analyses.

Importantly, we also revised the Limitations section of the discussion to explicitly acknowledge this issue **(Page 38 line 783–785)**. In the revised text, we now state that the correlational findings should be considered preliminary, that the possibility of Type I errors or unstable effect estimates cannot be ruled out, and that these findings require validation in larger independent samples.
