# Supplementary material for: Impaired midfrontal‑motor theta phase synchronization characterizes maladaptive motivational behavior in people with obsessive‑compulsive disorder
Source: PLoS Biol. 2026 Sep 1;24(9):e3003979. doi: 10.1371/journal.pbio.3003979 (PMC13568527; doi:10.1371/journal.pbio.3003979)
Supplement: Peer review history

# Editor report: Decision Letter 0

**DOI:** 10.1371/journal.pbio.3003979.r001

**Authors:** Christian Schnell, PhD

27 Mar 2026

Dear Dr Chen,

Thank you for submitting your manuscript entitled "Impaired midfrontal-motor theta phase synchronization drives maladaptive motivational behavior in obsessive-compulsive disorder" for consideration as a Research Article by PLOS Biology.

Your manuscript has now been evaluated by the PLOS Biology editorial staff as well as by an academic editor with relevant expertise and I am writing to let you know that we would like to send your submission out for external peer review.

However, before we can send your manuscript to reviewers, we need you to complete your submission by providing the metadata that is required for full assessment. To this end, please login to Editorial Manager where you will find the paper in the 'Submissions Needing Revisions' folder on your homepage. Please click 'Revise Submission' from the Action Links and complete all additional questions in the submission questionnaire.

Once your full submission is complete, your paper will undergo a series of checks in preparation for peer review. After your manuscript has passed the checks it will be sent out for review. To provide the metadata for your submission, please Login to Editorial Manager (https://www.editorialmanager.com/pbiology) within two working days, i.e. by Mar 31 2026 11:59PM.

If your manuscript has been previously peer-reviewed at another journal, PLOS Biology is willing to work with those reviews in order to avoid re-starting the process. Submission of the previous reviews is entirely optional and our ability to use them effectively will depend on the willingness of the previous journal to confirm the content of the reports and share the reviewer identities. Please note that we reserve the right to invite additional reviewers if we consider that additional/independent reviewers are needed, although we aim to avoid this as far as possible. In our experience, working with previous reviews does save time.

If you would like us to consider previous reviewer reports, please edit your cover letter to let us know and include the name of the journal where the work was previously considered and the manuscript ID it was given. In addition, please upload a response to the reviews as a 'Prior Peer Review' file type, which should include the reports in full and a point-by-point reply detailing how you have or plan to address the reviewers' concerns.

During the process of completing your manuscript submission, you will be invited to opt-in to posting your pre-review manuscript as a bioRxiv preprint. Visit http://journals.plos.org/plosbiology/s/preprints for full details. If you consent to posting your current manuscript as a preprint, please upload a single Preprint PDF.

Feel free to email us at plosbiology@plos.org if you have any queries relating to your submission.

Kind regards,

Christian

Christian Schnell, PhD

Senior Editor

PLOS Biology

cschnell@plos.org

# Peer review: Decision Letter 1

**DOI:** 10.1371/journal.pbio.3003979.r002

**Authors:** Christian Schnell, PhD

18 May 2026

Dear Dr Chen,

Thank you for your patience while your manuscript "Impaired midfrontal-motor theta phase synchronization drives maladaptive motivational behavior in obsessive-compulsive disorder" was peer-reviewed at PLOS Biology. It has now been evaluated by the PLOS Biology editors, an Academic Editor with relevant expertise, and by several independent reviewers.

In light of the reviews, which you will find at the end of this email, we would like to invite you to revise the work to thoroughly address the reviewers' reports.

As you will see below, the reviewers agree that the study is interesting and provides potentially important insights. However, they also raise a number of concerns, asking to be more cautious with the interpretation, provide more methodological details, and provide a number of additional analyses.

Given the extent of revision needed, we cannot make a decision about publication until we have seen the revised manuscript and your response to the reviewers' comments. Your revised manuscript is likely to be sent for further evaluation by all or a subset of the reviewers.

In addition to these revisions, you will need to complete some formatting changes, which you will receive in a follow up email. A member of our team will be in touch with a set of requests shortly.

We expect to receive your revised manuscript within 3 months. Please email us (plosbiology@plos.org) if you have any questions or concerns, or would like to request an extension.

At this stage, your manuscript remains formally under active consideration at our journal; please notify us by email if you do not intend to submit a revision so that we may withdraw it.

**IMPORTANT - SUBMITTING YOUR REVISION**

Your revisions should address the specific points made by each reviewer. Please submit the following files along with your revised manuscript:

1. A 'Response to Reviewers' file - this should detail your responses to the editorial requests, present a point-by-point response to all of the reviewers' comments, and indicate the changes made to the manuscript.

*NOTE: In your point-by-point response to the reviewers, please provide the full context of each review. Do not selectively quote paragraphs or sentences to reply to. The entire set of reviewer comments should be present in full and each specific point should be responded to individually, point by point.

You should also cite any additional relevant literature that has been published since the original submission and mention any additional citations in your response.

2. In addition to a clean copy of the manuscript, please also upload a 'track-changes' version of your manuscript that specifies the edits made. This should be uploaded as a "Revised Article with Changes Highlighted" file type.

*Re-submission Checklist*

When you are ready to resubmit your revised manuscript, please refer to this re-submission checklist: https://plos.io/Biology_Checklist

To submit a revised version of your manuscript, please go to https://www.editorialmanager.com/pbiology/ and log in as an Author. Click the link labelled 'Submissions Needing Revision' where you will find your submission record.

Please make sure to read the following important policies and guidelines while preparing your revision:

*Published Peer Review*

Please note while forming your response, if your article is accepted, you may have the opportunity to make the peer review history publicly available. The record will include editor decision letters (with reviews) and your responses to reviewer comments. If eligible, we will contact you to opt in or out. Please see here for more details:

https://blogs.plos.org/plos/2019/05/plos-journals-now-open-for-published-peer-review/

*PLOS Data Policy*

Please note that as a condition of publication PLOS' data policy (http://journals.plos.org/plosbiology/s/data-availability) requires that you make available all data used to draw the conclusions arrived at in your manuscript. If you have not already done so, you must include any data used in your manuscript either in appropriate repositories, within the body of the manuscript, or as supporting information (N.B. this includes any numerical values that were used to generate graphs, histograms etc.). For an example see here: http://www.plosbiology.org/article/info%3Adoi%2F10.1371%2Fjournal.pbio.1001908#s5

*Blot and Gel Data Policy*

We require the original, uncropped and minimally adjusted images supporting all blot and gel results reported in an article's figures or Supporting Information files. We will require these files before a manuscript can be accepted so please prepare them now, if you have not already uploaded them. Please carefully read our guidelines for how to prepare and upload this data: https://journals.plos.org/plosbiology/s/figures#loc-blot-and-gel-reporting-requirements

*Protocols deposition*

To enhance the reproducibility of your results, we recommend that if applicable you deposit your laboratory protocols in protocols.io, where a protocol can be assigned its own identifier (DOI) such that it can be cited independently in the future. Additionally, PLOS ONE offers an option for publishing peer-reviewed Lab Protocol articles, which describe protocols hosted on protocols.io. Read more information on sharing protocols at https://plos.org/protocols?utm_medium=editorial-email&utm_source=authorletters&utm_campaign=protocols

Thank you again for your submission to our journal. We hope that our editorial process has been constructive thus far, and we welcome your feedback at any time. Please don't hesitate to contact us if you have any questions or comments.

Sincerely,

Christian

Christian Schnell, PhD

Senior Editor

PLOS Biology

cschnell@plos.org

------------------------------------

REVIEWS:

Reviewer #1: The authors used an established motivational go/no-go task and EEG, with computational modeling, to test maladaptive behaviors in OCD patients vs healthy control (HC). Results showed OCD patients had larger Pavlovian bias and lower learning rates. Important, midfrontal theta power its synchrony with task related regions further captured behavioral differences in OCD and HC.

This paper is generally well-written, with concepts/constructs explained and methods justified. The questions below are meant to improve the manuscript.

MAJOR:

(1) It is very helpful to introduce the "insight-action dissociation", but it remains less frequently mentioned in the remaining of the manuscript. It'd be ideal to refer to back to it in the Discussion section to reconcile all the findings on a bigger picture.

(2) I am not entirely sure why the cortico-striato-thalamo-cortical (CSTC) circuits are mentioned in the Intro. The current method is EEG, and the CSTC is not directly examined. Perhaps it could be discussed in the wider literature in the Discussion rather than the Intro. (or, it can be mentioned in the Intro, but it needs to be better motivated/justified.)

(3) Regarding the behavioral analysis, by reading SI S4, it seems the analyses were quite thoroughly done, but it would be helpful to include key information in the text. By reading the Results (from Line 140), it seems that a few separate tests were used, but actually all analyses were linear mixed effect models. It would be helpful to briefly include these details (e.g., Linear mixed effect models with 2 within-subject predictors (action, valence)). This would make sure that readers will not misunderstand the analyses.

(4) Regarding ERP, though it is acknowledged that the N2 component has mixed findings, it would still be helpful to conduct the analysis which can be a valuable addition to the literature (it can be included in the SI).

(5) For model M4(a-d), it is a good idea to see whether midfrontal theta power can further explain the data. For M4b, and the key equation (7), I wonder why it is a minus sign in (1-beta*theta)*Q? The main text did not have the equation, so it is not straightforward to interpret a negative beta parameter with increased Q value. Also Q is not a parameter (Line 327) but a model internal variable (or decision variable). And for M4c, I am not sure if it is needed. It is not included in the M3 model family, and it is not winning anyway. I think it can add more confusion than value. Last, it would be helpful to more explicitly map Equations 6-9 to the models M4a-d. Last, it seems counterintuitive to have M4 models in the methods but M5 models in the SI.

(6) I feel the narrative around M5 models somewhat not straightforward. It is unnatural to first introduce M5c-d and then M5a-b. It would be more helpful to have a more streamlined approach here.

(7) More critically, the inclusion of effects of phase synchrony in models seems to be separate for Pavlovian bias (pi) and value (Q), but Fig 6 A-B is plotting them in the same space. I find this approach somewhat problematic, as the two parameters being plotted (Eg. F6A-B) do not belong to the same model. I think the rationale makes sense, but if the two parameters are estimated in two models, they may capture overlapping variances than if the model is estimated in one go (ie, testing the synchrony effect both for pi and Q in the same model).

(8) Regardless, I quite like the approach of computing the orientation angle (S6), and I think this should be included (and visualized) in the main text. In the end, OCD patients are being tested, it would be very valuable to show the clinically meaningful results in a more explicit manner. Relatedly, just for the winning models without the EEG data, I wonder if the Pavlovian Bias and learning rates (the two that show differences between OCD and HC) are also correlated with OCD symptoms?

(9) As learning rate is also a parameter that differed between OCD and HC, I am curious whether any EEG characteristics can also be examined?

MINOR:

(1) It would be helpful to more explicitly state the modeling is done under the hierarchical Bayesian framework in the main text. It'd also be beneficial to reference modeling practice papers on the workflow (e.g., Palminteri et al., 2017; Zhang et al., 2020)

(2) It would be helpful to cite some EEG work on the similar task (e.g., Algermissen et al., 2024)

References:

Algermissen, J., Swart, J. C., Scheeringa, R., Cools, R., & den Ouden, H. E. (2024). Prefrontal signals precede striatal signals for biased credit assignment in motivational learning biases. Nature communications, 15(1), 19.

Palminteri, S., Wyart, V., & Koechlin, E. (2017). The importance of falsification in computational cognitive modeling. Trends in cognitive sciences, 21(6), 425-433.

Zhang, L., Lengersdorff, L., Mikus, N., Gläscher, J., & Lamm, C. (2020). Using reinforcement learning models in social neuroscience: frameworks, pitfalls and suggestions of best practices. Social Cognitive and Affective Neuroscience, 15(6), 695-707.

Reviewer #2 (M. Prabhavi N. Perera signed her report): Please see attachment

Reviewer #3: This manuscript titled "Impaired midfrontal-motor theta phase synchronization drives maladaptive motivational behavior in obsessive-compulsive disorder" investigated the relationships between EEG and computational model parameters in participants with OCD using a modified Go/NoGo task. In a sample of 73 participants with about equal OCD (36) and healthy control (37) participants, the authors found that midfrontal theta phase synchrony was associated with maladaptive biases in OCD while theta power likely did not influence outcome. This is an interesting project that elucidates the roles of time-frequency EEG activities through linking them to computational modeling. The authors were very thorough in comparing multiple models. Although I was able to generally follow the computational model descriptions, it is not my expertise and my review will focus on EEG.

Major

1. I suggest the authors describe the EEG preprocessing and extraction in more detail. For example, what were the parameters were used to remove artifacts using ICA (manual rejection or automatic), how many (or what proportion of) ICAs were removed, how many trials were retained/removed, how was the theta extraction window decided, how cue-locked theta enhancement time was defined, etc. Ideally, authors report enough for someone with a similar data to be able to replicate the study.

2. Pages 13 and 14. Could the authors test for the theta enhancement and RT difference statistically? Also, the theta enhancement in Figure 4 seems to be over a narrower window in HC, but more spread out in OCD. Does that reflect more heterogeneity in OCD or do participants with OCD have elongated enhancement (rather than slower enhancement)? This is the finding that the authors base their argument on theta power not influencing choice, so a thorough statistical investigation would help.

Minor

1. I suggest the authors report the number of trials per condition that were used for the computational and EEG analyses. Particularly confirming that there were sufficient trials for the variables to stabilize would be helpful.

2. The sample sizes are small as the authors acknowledge, so some results (such as correlations) should be interpreted carefully. The possibility of Type 1 error should be noted in the limitation.

Attachment Submitted filename: Review.docx

# Author comment: Author response to Decision Letter 2

**DOI:** 10.1371/journal.pbio.3003979.r003

6 Jul 2026

Attachment Submitted filename: Response to Reviewers.docx

# Peer review: Decision Letter 2

**DOI:** 10.1371/journal.pbio.3003979.r004

**Authors:** Christian Schnell, PhD

7 Aug 2026

Dear Dr Chen,

Thank you for your patience while we considered your revised manuscript "Impaired midfrontal-motor theta phase synchronization characterizes maladaptive motivational behavior in obsessive-compulsive disorder" for publication as a Research Article at PLOS Biology. This revised version of your manuscript has been evaluated by the PLOS Biology editors, the Academic Editor and two of the original reviewers.

Based on the reviews and on our Academic Editor's assessment of your revision, we are likely to accept this manuscript for publication, provided you satisfactorily address the following data and other policy-related requests:

* Please include information in the Methods section whether the study has been conducted according to the principles expressed in the Declaration of Helsinki.

* Please revise the language in your manuscript from "patients with OCD" to "people with OCD". It is more appropriate to use person-first language when discussing people with OCD unless they are actively patients in a medical setting (which is not the case here).

* Please also move the supplementary text from the supplementary information files to the main manuscript. We do not have a word count limit and want to ensure it is as easy as possible for readers to find the information they are seeking.

*Please also make sure that all references are moved to the main reference list (or are listed there in addition). The literature databases do not pick up references from supplementary information files, so authors do not get credict when their paper is only cited there.

* If you have any text left that should remain in the supplementary information files, please provide it within a single file SI file.

* DATA POLICY:

You may be aware of the PLOS Data Policy, which requires that all data be made available without restriction: http://journals.plos.org/plosbiology/s/data-availability. For more information, please also see this editorial: http://dx.doi.org/10.1371/journal.pbio.1001797

Note that we do not require all raw data. Rather, we ask that all individual quantitative observations that underlie the data summarized in the figures and results of your paper be made available in one of the following forms:

1) Supplementary files (e.g., excel). Please ensure that all data files are uploaded as 'Supporting Information' and are invariably referred to (in the manuscript, figure legends, and the Description field when uploading your files) using the following format verbatim: S1 Data, S2 Data, etc. Multiple panels of a single or even several figures can be included as multiple sheets in one excel file that is saved using exactly the following convention: S1_Data.xlsx (using an underscore).

2) Deposition in a publicly available repository. Please also provide the accession code or a reviewer link so that we may view your data before publication.

Regardless of the method selected, please ensure that you provide the individual numerical values that underlie the summary data displayed in the following figure panels as they are essential for readers to assess your analysis and to reproduce it: 2CF, 4DF and 5ABCD.

NOTE: the numerical data provided should include all replicates AND the way in which the plotted mean and errors were derived (it should not present only the mean/average values).

Please also ensure that figure legends in your manuscript include information on where the underlying data can be found, and ensure your supplemental data file/s has a legend.

Please ensure that your Data Statement in the submission system accurately describes where your data can be found.

* CODE POLICY

Per journal policy, if you have generated any custom code during the course of this investigation, please make it available without restrictions. Please ensure that the code is sufficiently well documented and reusable, and that your Data Statement in the Editorial Manager submission system accurately describes where your code can be found. More information on our Code Policy, what and how to share can be found here: https://journals.plos.org/plosbiology/s/code-availability

Please note that we cannot accept sole deposition of code in GitHub, as this could be changed after publication. However, you can archive this version of your publicly available GitHub code to Zenodo. Once you do this, it will generate a DOI number, which you will need to provide in the Data Accessibility Statement (you are welcome to also provide the GitHub access information). See the process for doing this here: https://docs.github.com/en/repositories/archiving-a-github-repository/referencing-and-citing-content

As you address these items, please take this last chance to review your reference list to ensure that it is complete and correct. If you have cited papers that have been retracted, please include the rationale for doing so in the manuscript text, or remove these references and replace them with relevant current references. Any changes to the reference list should be mentioned in the cover letter that accompanies your revised manuscript.

In addition to these revisions, you may need to complete some formatting changes, which you will receive in a follow up email. A member of our team will be in touch with a set of requests shortly. If you do not receive a separate email within a few days, please assume that checks have been completed, and no additional changes are required.

We expect to receive your revised manuscript within two weeks.

To submit your revision, please go to https://www.editorialmanager.com/pbiology/ and log in as an Author. Click the link labelled 'Submissions Needing Revision' to find your submission record. Your revised submission must include the following:

- a cover letter that should detail your responses to any editorial requests, if applicable, and whether changes have been made to the reference list

- a Response to Reviewers file that provides a detailed response to the reviewers' comments (if applicable, if not applicable please do not delete your existing 'Response to Reviewers' file.)

- a track-changes file indicating any changes that you have made to the manuscript.

NOTE: If Supporting Information files are included with your article, note that these are not copyedited and will be published as they are submitted. Please ensure that these files are legible and of high quality (at least 300 dpi) in an easily accessible file format. For this reason, please be aware that any references listed in an SI file will not be indexed. For more information, see our Supporting Information guidelines:

https://journals.plos.org/plosbiology/s/supporting-information

*Published Peer Review History*

Please note that you may have the opportunity to make the peer review history publicly available. The record will include editor decision letters (with reviews) and your responses to reviewer comments. If eligible, we will contact you to opt in or out. Please see here for more details:

https://plos.org/published-peer-review-history/

*Press*

Should you, your institution's press office or the journal office choose to press release your paper, please ensure you have opted out of Early Article Posting on the submission form. We ask that you notify us as soon as possible if you or your institution is planning to press release the article.

*Protocols deposition*

To enhance the reproducibility of your results, we recommend that if applicable you deposit your laboratory protocols in protocols.io, where a protocol can be assigned its own identifier (DOI) such that it can be cited independently in the future. Additionally, PLOS ONE offers an option for publishing peer-reviewed Lab Protocol articles, which describe protocols hosted on protocols.io. Read more information on sharing protocols at https://plos.org/protocols?utm_medium=editorial-email&utm_source=authorletters&utm_campaign=protocols

Please do not hesitate to contact me should you have any questions.

Sincerely,

Christian

Christian Schnell, PhD

Senior Editor

cschnell@plos.org

PLOS Biology

------------------------------------------------------------------------

Reviewer remarks:

Reviewer #1: The authors have done a very thorough revision that has addressed my previous questions and queries.

I much appreciated the added narrative and theory-driven descriptions, and I believe this manuscript is ready to be accepted.

Reviewer #2 (Prabhavi N. Perera has signed her report): The authors have carefully considered all recommendations, and revised the manuscript accordingly. I believe the current version is ready for publication.

# Author comment: Author response to Decision Letter 3

**DOI:** 10.1371/journal.pbio.3003979.r005

16 Aug 2026

Attachment Submitted filename: Response_to_Reviewers_auresp_3.docx

# Editor report: Decision Letter 3

**DOI:** 10.1371/journal.pbio.3003979.r006

**Authors:** Christian Schnell, PhD

17 Aug 2026

Dear Qi,

Thank you for the submission of your revised Research Article "Impaired midfrontal-motor theta phase synchronization characterizes maladaptive motivational behavior in obsessive-compulsive disorder" for publication in PLOS Biology. On behalf of my colleagues and the Academic Editor, Yiheng Tu, I am pleased to say that we can in principle accept your manuscript for publication, provided you address any remaining formatting and reporting issues. These will be detailed in an email you should receive within 2-3 business days from our colleagues in the journal operations team; no action is required from you until then. Please note that we will not be able to formally accept your manuscript and schedule it for publication until you have completed any requested changes.

Please take a minute to log into Editorial Manager at http://www.editorialmanager.com/pbiology/, click the "Update My Information" link at the top of the page, and update your user information to ensure an efficient production process.

PRESS

We frequently collaborate with press offices. If your institution or institutions have a press office, please notify them about your upcoming paper at this point, to enable them to help maximize its impact. If the press office is planning to promote your findings, we would be grateful if they could coordinate with biologypress@plos.org. If you have previously opted in to the early version process, we ask that you notify us immediately of any press plans so that we may opt out on your behalf.

We also ask that you take this opportunity to read our Embargo Policy regarding the discussion, promotion and media coverage of work that is yet to be published by PLOS. As your manuscript is not yet published, it is bound by the conditions of our Embargo Policy. Please be aware that this policy is in place both to ensure that any press coverage of your article is fully substantiated and to provide a direct link between such coverage and the published work. For full details of our Embargo Policy, please visit http://www.plos.org/about/media-inquiries/embargo-policy/.

Thank you again for choosing PLOS Biology for publication and supporting Open Access publishing. We look forward to publishing your study.

Sincerely,

Christian

Christian Schnell, PhD

Senior Editor

PLOS Biology

cschnell@plos.org
